# Design of aminoanthraquinone-based heterogeneous photocatalysts for visible-light-driven reactions and antibacterial applications†

**DOI:** 10.1039/d5ra03539b

**Published:** 2025-07-11

**Authors:** M. Guadalupe Martin, Lucio R. Clavero, Camila S. Gomez, Virginia Aiassa, Juan M. Lázaro-Martínez, Sandra E. Martín, Paula M. Uberman, María E. Budén

**Affiliations:** a Departamento de Química Orgánica, Facultad de Ciencias Químicas, Universidad Nacional de Córdoba, Haya de la Torre y Medina Allende Córdoba Córdoba X5000HUA Argentina paula.uberman@unc.edu.ar eugebuden@unc.edu.ar; b Instituto de Investigaciones en Fisicoquímica, INFIQC-CONICET-Universidad Nacional de Córdoba, Haya de la Torre y Medina Allende Córdoba Córdoba X5000HUA Argentina; c Departamento de Ciencias Farmacéuticas, Facultad de Ciencias Químicas, Universidad Nacional de Córdoba, Haya de la Torre y Medina Allende Córdoba Córdoba X5000HUA Argentina; d Unidad de Investigación y Desarrollo en Tecnología Farmacéutica, UNITEFA-CONICET, Universidad Nacional de Córdoba, Haya de la Torre y Medina Allende Córdoba Córdoba X5000HUA Argentina; e Universidad de Buenos Aires, Facultad de Farmacia y Bioquímica, Departamento de Ciencias Químicas Junín 956 Ciudad Autónoma de Buenos Aires C1113AAD Argentina; f Instituto Química y Metabolismo del Fármaco, IQUIMEFA-UBA-CONICET Junín 956 Ciudad Autónoma de Buenos Aires C1113AAD Argentina

## Abstract

Photoredox catalysis driven by visible light provides an efficient and environmentally friendly strategy for organic synthesis. In this study, we report a novel heterogeneous photocatalyst developed by immobilizing 1,5-diaminoanthraquinone (DAAQ) onto functionalized silica nanoparticles (SNPs). The photocatalyst was thoroughly characterized using various techniques, including TEM, SEM, UV-vis, FT-IR, ss-NMR, DLS, and XPS. Its photocatalytic performance was assessed in three reaction systems: the dehalogenation of aryl halides, cross-dehydrogenative coupling (CDC) of *N*-aryl-1,2,3,4-tetrahydroisoquinolines, and singlet oxygen generation *via* energy transfer. The SNPs-DAAQ catalyst showed high photocatalytic activity and excellent recyclability, achieving up to 10 cycles. Additionally, the nanomaterial exhibited promising antibacterial properties against *Staphylococcus aureus*. The catalyst's robust performance and potential applications in green chemistry and antibacterial devices highlight its value in designing sustainable synthetic methodologies.

## Abstract 2

The heterogeneous SNPs-DAAQ photocatalyst enables sustainable organic synthesis through visible-light-driven photoredox catalysis, offering recyclability and additional antibacterial properties.

## Graphical abstract

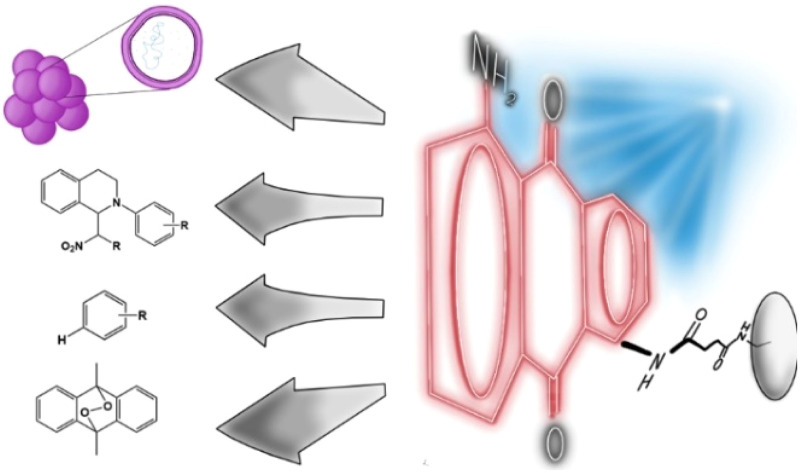

## Introduction

Photoredox catalysis has revolutionized organic synthesis over the past decade, emerging as an efficient and sustainable technique for generating reactive intermediates using visible light as a reagent.^1–6^ Moreover, visible light photocatalysis enables reactions to proceed under mild conditions, reducing energy consumption and waste generation, thereby aligning with the principles of green chemistry. Transition metal complexes, particularly those based on Ru and Ir, are widely used as photocatalysts (PCs) due to their strong visible-light absorption, high oxidation and reduction potentials, and stability under synthetic conditions.^7–9^ However, the high cost and toxicity of these complexes have driven the development of more sustainable alternatives, such as organic dyes.^10^ These dyes, including eosin Y,^11^ acridine,^12–14^ riboflavin,^15–17^ rose bengal,^18,19^ and rhodamine^20^ derivatives, have gained increasing attention due to their low toxicity and their efficient absorption in the visible region, making them ideal for synthetic transformations. This evolution highlights the significance of photoredox catalysis as a versatile tool for developing sustainable and efficient synthetic methodologies.^21^

Among the new classes of organic PCs, anthraquinones (AQs) have proven to be highly effective in electron transfer reactions, presenting applications in the production of pharmaceuticals and advanced materials development.^21^ Additionally, AQ derivatives are versatile natural dyes involved in various biological systems.^22^ The photophysical and photochemical behavior of several AQs photocatalysts has been extensively investigated, and AQs are widely used in the dyestuff industry because their triplet excited states are among the most strongly oxidizing agents available. The presence of different functional groups on AQ scaffold profoundly affects their UV-vis spectra, as do hydrogen bonds formation and other intramolecular interactions. These characteristics significantly impact their photophysical properties and reactivity in photo-electron transfer (PET) processes. Electron-donating groups, such as amino groups in diaminoanthraquinones (DAAQ), shift the absorption band into the visible region of the UV-vis. Particularly, 1,5-diaminoanthraquinones (1,5-DAAQ) exhibits strong absorbance maxima near 400 nm corresponding to a n–π* band, along with an additional π–π* charge transfer (CT) band between 468-490 nm, depending on the nature solvent. Furthermore, its fluorescence emission ranges from 545 to 590 nm.^23^

DAAQs have also been employed in photocatalytic synthetic approaches. Brasholz and colleagues explored the photocatalytic cross-dehydrogenative coupling (CDC) reaction of *N*-aryl-1,2,3,4-tetrahydroisoquinolines under basic aerobic conditions, using 1,5-DAAQ as PC.^24^ Additionally, 1,5-DAAQ demonstrated high efficiency in synthesizing imines from benzylamines and *cis*-1,3-diaryltetrahydroisoquinolines from branched aldimines through aerobic oxidative dehydrogenation reactions.^25^ Furthermore, 1,5-DAAQ was identified as the optimal catalyst for photooxygenation reactions, enabling the synthesis of valuable spirooxindole-1,3-oxazines from parent indole alkaloids.^26^ Moreover, 1,5-DAAQ was combined with an iodonium salt and various aromatic tertiary amines to develop a novel photoinitiator system for the free radical polymerization of (meth)acrylates.^27^

A widely used strategy for developing recyclable catalysts with innovative properties is the application of nanotechnology as a tool for generating new heterogeneous nanocatalysts.^28^ The utilization of novel nanomaterials as catalysts offers the advantage of combining the characteristics of homogeneous catalysts (high efficiency and selectivity) with the ease of recovery and recycling typical of heterogeneous systems. Numerous examples of PCs immobilized on nanomaterials have been reported. Recently, various organic and organometallic PCs have recently been immobilized onto different types of silica materials, leading to the new heterogeneous PCs.^29–32^ A common strategy for preparing heterogeneous PCs involves anchoring homogeneous photocatalysts onto silica-based supports such as silica nanoparticles,^33–37^ glass wool,^38^ glass beads,^39^ and mesoporous silica.^40–45^ Some of these approaches rely on electrostatic interactions between the physis-absorbed catalytic species and the support, frequently resulting in catalyst desorption into the solution during use.^46^ For the development of durable, long-lived heterogeneous PC, the formation of strong covalent bonds between the photocatalyst and support is essential. Following this strategy, we have previously utilized inexpensive and widely available materials, such as silica nanoparticles, as catalysts support for anchoring 2-carboxylic acid anthraquinone^47^ or eosin Y PCs.^48^ In both cases, we demonstrated that these materials are suitable for use in organic synthesis.

Additionally, anthraquinone derivatives (AQs) are potent photosensitizers capable of generating reactive oxygen species (ROS), such as superoxide anion, hydroxyl radical, and singlet oxygen, which contribute to their antibacterial property.^49^ These compounds show great potential for application in photodynamic therapy (PDT), a technique that employs photosensitizers and light (visible or ultraviolet) to treat localized infections such as dental caries, periodontal diseases, oral candidiasis, and infected wounds.^50^ The overuse of antibiotics has led to significant bacterial resistance, giving rise to so-called “super bacteria”. In recent years, antibacterial nanomaterials have emerged as effective solutions to combat bacterial drug resistance,^51^ offering advantages such as high efficiency, low dosages, minimal acute toxicity, and cost-effectiveness.^52^ However, the development of antibacterial nanomaterials modified by anthraquinones capable of achieving high yields of ROS in photo-induced sterilization system, remains relatively unexplored.^53,54^

This work reports the design of a novel heterogeneous PC based on the immobilization of 1,5-DAAQ onto functionalized silica nanoparticles. The photocatalyst was comprehensively characterized using microscopic and spectroscopic techniques. Its performance was evaluated in various organic transformations, with particular emphasis on its recovery and recyclability. Specifically, it was studied in dehalogenation of aryl halides, CDC reactions, and energy transfer reactions for the generation of singlet oxygen. Finally, its antibacterial activity was assessed, revealing promising potential for antimicrobial applications.

## Results and discussion

### Synthesis and characterization of SNPs-DAAQ photocatalyst

The anthraquinone-based heterogeneous photocatalyst was synthesized by the immobilization of 1,5-diaminoanthraquinone (DAAQ) onto the surface of silica nanoparticles functionalized with carboxylic acid groups (SNPs-COOH) through a direct amidation reaction (eqn 1). The SNPs-COOH were obtained by functionalization of SNPs with APTES and succinic acid (see ESI, Sections S2.1 and S2.2†). The amidation reaction was facilitated by *N*,*N*′-dicyclohexylcarbodiimide (DCC) and hydroxybenzotriazole (HOBt) as activating agents for the carboxyl group, enabling the formation of stable amide bonds between the carboxyl groups of the SNPs and the amino groups of the DAAQ. The resulting nanomaterial, named SNPs-DAAQ, was centrifuged, thoroughly washed, dried, and stored under ambient conditions, for subsequent use.
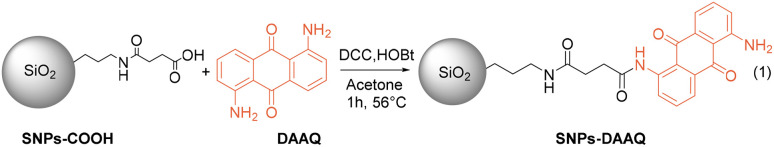


The as-obtained nanomaterials were thoroughly characterized using a range of techniques, including scanning electron microscopy (SEM), transmission electron microscopy (TEM), solid-state fluorescence spectroscopy and fluorescence spectroscopy in solution, UV-visible spectroscopy (UV-vis), Fourier transform infrared spectroscopy (FT-IR), solid-state nuclear magnetic resonance (ss-NMR), X-ray photoelectron spectroscopy (XPS) and Dynamic Light Scattering (DLS).

**Fig. 1:**
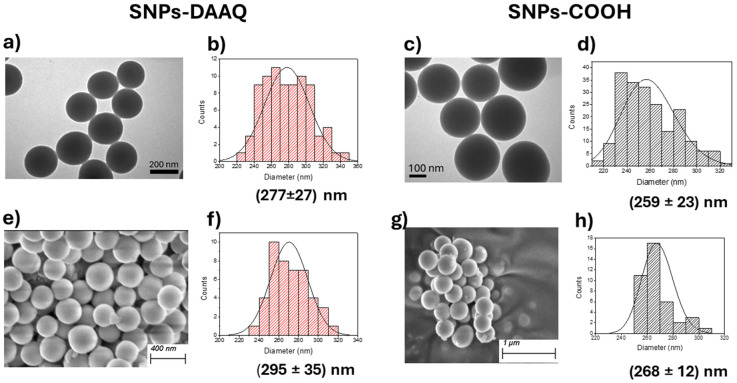
(a) TEM micrograph and (b) size distribution analysis for SNPs-DAAQ. (c) TEM micrograph and (d) size distribution analysis for SNPs-COOH. (e) SEM micrograph and (f) size distribution analysis for SNPs-DAAQ. (g) SEM micrograph and (h) size distribution analysis for SNPs-COOH.

The morphology and size of the nanoparticles (NPs) were examined by TEM and SEM. TEM analysis showed that the NPs were spherical, with mean diameters of (259 ± 23) nm for SNPs-COOH (Fig. 1d), and (277 ± 27) nm for SNPs-DAAQ (Fig. 1b), in agreement with the SEM results. According to SEM analysis, the precursor SNPs-COOH exhibited an average particle size of (268 ± 12) nm (Fig. 1h), while SNPs-DAAQ had an average diameter of (295 ± 35) nm (Fig. 1f). Additionally, SEM micrographs revealed the formation of densely packed particles.

**Fig. 2:**
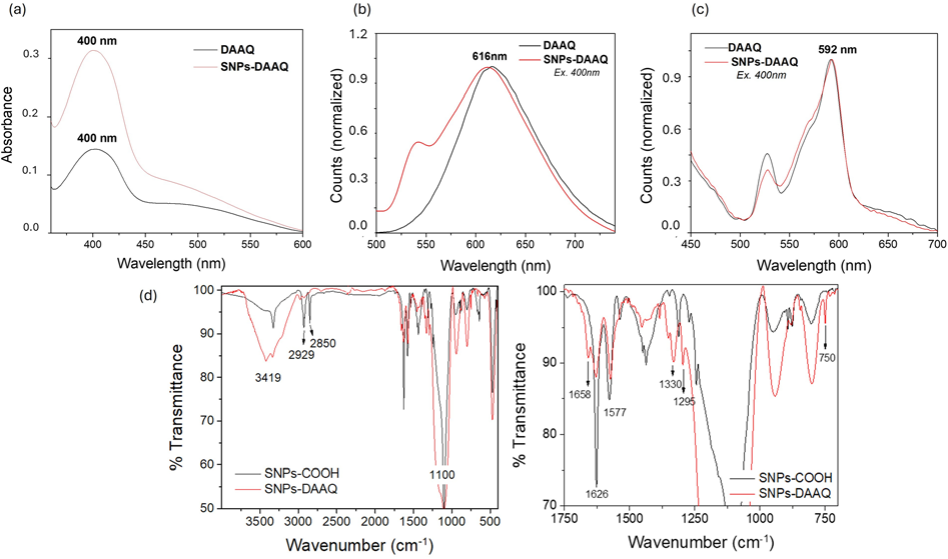
(a) Comparative UV-vis spectrum for SNPs-DAAQ suspension in ethanol (2 mg mL^−1^), and DAAQ in ethanol solution (1 × 10^−4^ M); (b) comparative fluorescence spectroscopy for SNPs-DAAQ suspension in ethanol (2 mg mL^−1^), and DAAQ in ethanol ([DAAQ] = 1 × 10^−4^ M, *λ*_exc_ = 400 nm); (c) comparative solid-state fluorescence of SNPs-DAAQ and DAAQ; (d) comparative FT-IR analysis for SNPs-COOH (black) and SNPs-DAAQ (red).

The UV-visible spectra of an ethanolic solution containing 1,5-DAAQ and an ethanolic suspension of SNPs-DAAQ were analyzed (Fig. 2a), detecting an absorption maximum at 400 nm for both the homogeneous and heterogeneous photocatalysts. For SNPs-DAAQ, the UV-visible spectra were obtained both before and after baseline correction to account for light scattering effects caused by the nanoparticles. The identical absorption maxima in both spectra confirm that the immobilizing of the anthraquinone photocatalyst onto SNPs does not alter its intrinsic absorption properties, in agreement with our previous observations with other organic dyes.^47,48^ The amount of DAAQ covalently bound to the surface of the SNPs was determined using UV-vis spectroscopy (see ESI, Section 4.2†). Based on the measured absorbance values, a DAAQ loading of 3.74% (w/w) was determined, corresponding to 0.157 mmol of DAAQ per gram of nanomaterial. These values were subsequently used to evaluate the photocatalytic activity of SNPs-DAAQ.

Fluorescence measurements were conducted on an ethanolic solution of DAAQ and an ethanolic suspension of SNPs-DAAQ, both exhibiting an emission maximum at 616 nm (Fig. 2b). Additionally, solid-state fluorescence spectra were measured for SNPs-DAAQ and DAAQ, with both spectra showing a maximum at 592 nm under the same excitation wavelength of 400 nm (Fig. 2c). These results indicate that the fluorescence properties of DAAQ are preserved upon immobilization onto the silica nanoparticles, both in solution and in the solid state.

The successful incorporation of DAAQ into the SNPs material was confirmed by FT-IR and ss-NMR. The FT-IR spectra of SNPs-DAAQ and SNPs-COOH (Fig. 2d) revealed characteristic absorption bands of SNPs, including a broad peak around 1100 cm^−1^ corresponding to Si–O–Si stretching vibrations, a broad band at 3419 cm^−1^ associated with the O–H stretching of SiO–H groups, and a shoulder at 950 cm^−1^ corresponding to the Si–OH bending, simultaneously with the presence of physically adsorbed water.^32,37^ Both spectra also exhibited absorption bands at 2929 cm^−1^ and 2850 cm^−1^, corresponding to aliphatic C–H bonds derived from APTES functionalization.

The spectrum of SNPs-COOH exhibited a peak at 1626 cm^−1^, attributed to C=O bond stretching (amide I), along with another at 1577 cm^−1^ (amide II), indicating the formation of an amide bond between SNPs-NH_2_ and succinic acid. In contrast, the spectrum of SNPs-DAAQ displayed new signals at 1658 cm^−1^, corresponding to the carbonyl group of DAAQ; at 1330 cm^−1^ and 1295 cm^−1^, associated with vibration of the anthraquinone scaffold; and at 750 cm^−1^, corresponding to the out-of-plane bending of Csp^2^–H bonds in aromatic anthraquinone rings. These spectral features, which are consistent with those of the organic dye (Fig. SI5†), confirm the successful incorporation of DAAQ into the functionalized SNPs.

**Fig. 3:**
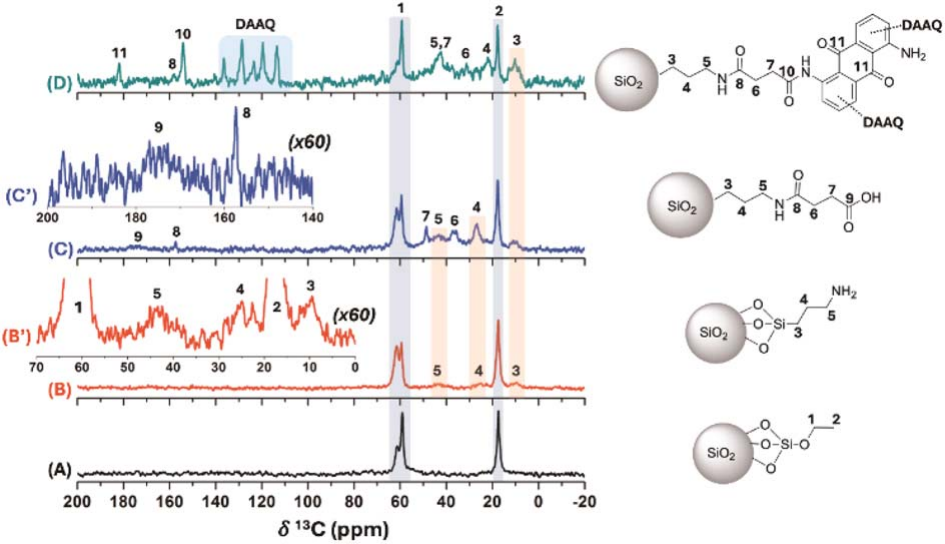
^13^C CP-MAS spectra (MAS rate: 13 kHz) for the SNPs (A), SNPs-NH_2_ (B), SNPs-COOH (C) and SNPs-DAAQ (D) samples. Particularly, the extended regions of the ^13^C CP-MAS spectra are shown for the SNPs-NH_2_ (B′) and SNPs-COOH (C′).

To investigate the organic derivatives present in SiO_2_ nanoparticles at the atomic level, ss*-*^1^H-NMR (Fig. SI6†), ^29^Si-NMR (Fig. SI7†) and ^13^C-NMR (Fig. 3) analyses were performed. The ^1^H ss-NMR spectra off the different SNPs show a significant contribution from silanol groups and physisorbed water molecules to the NMR signal, which hindered the clear identification of chemical modifications on the silica structure at a MAS rate of 30 kHz (Fig. SI6†). Similarly, chemical modification could not be inferred from the ^29^Si direct polarization ss-NMR experiments, as the T^n^ bands were not observed,^55^ likely due to the low degree of modification related to the silica core (Fig. SI7†). On the other hand, the ^13^C CP-MAS spectra provided clear evidence of chemical resonance signals associated with the different modifications performed on the SNPs (Fig. 3), acquired at a MAS rate of 13 kHz. Specifically, the ^13^C CP-MAS spectrum of unmodified SNPs showed signals corresponding to residual oxygenated structures and aliphatic hydrocarbons at ^13^C chemical shift (*δ*^13^C) of 61.6/59.0 ppm and 17.3 ppm, respectively (Fig. 3A). These signals are typically associated to residual ethoxy groups from TEOS used during the synthesis of the SNPs. This was supported by elemental analysis for the SNPs, which showed 0.87% of carbon content associated to the residual ethoxy moieties in this sample (Table SI2†). With the different chemical modifications of the SNPs, both the carbon and nitrogen content increase, as confirmed by organic elemental analysis.

The ^13^C CP-MAS spectrum of SNPs-NH_2_ displayed resonance signals corresponding to the propylamine moieties introduced *via* APTES, along with residual ethoxy groups signals (Fig. 3B). Noteworthy, despite a relatively low signal-to-noise ratio in the ^13^C CP-MAS spectrum and the low nitrogen content, chemical modification with APTES was confirmed by ^13^C ss-NMR and elemental analysis for the SNPs-NH_2_ sample (Fig. 3B′ and Table SI2†). Subsequent modification with succinic acid led to the appearance of characteristic signals in the ^13^C CP-MAS spectrum of SNPs–COOH, with methylene carbon resonance (*δ*^13^C = 48.8 and 36.6 ppm) and an amide carbon groups (*δ*^13^C = 157.3 ppm, Fig. 3C). Additionally, the free carboxylic acid group signal appeared as a broad peak at *δ*^13^C ∼ 175 ppm (Fig. 3C′). Finally, the incorporation of the DAAQ structure into the SNPs-DAAQ was confirmed by the presence of sp^2^-hybridized carbon atoms signals in the aromatic region (110-140 ppm) in the SNPs-DAAQ spectrum (Fig. 3D). Besides, the carbonyl resonances of the amide and ketone groups were observed at *δ*^13^C value of 154.2 and 189.1 ppm, respectively. Notably, the resonance signal of the amide carbon directly bound to the DAAQ structure exhibited a chemical shift at *δ*^13^C = 154.2 ppm, distinct from the adjacent amide group closer to the nanoparticle core (*δ*^13^C = 157.3 ppm), further confirming successful amidation (Fig. 3D). Additionally, modifications in the methylene region (*δ*^13^C ∼ 20–50 ppm) were observed, consistent with structural changes resulting from DAAQ incorporation.

Furthermore, XPS analysis was carried out to investigate the surface composition of the nanomaterial (Table SI3†). The predominant component in the heterogeneous photocatalyst SNPs-DAAQ was identified as SiO_2_, with nitrogen content representing approximately 5% of the relative atomic composition. A comparative analysis of the XPS spectra of SNPs-NH_2_ precursor and SNPs-DAAQ revealed an increase in the atomic percentages of both carbon and nitrogen in SNPs-DAAQ. Specifically, the carbon content in this material was 28%, compared to 13% (coming from APTES) in SNPs-NH_2_, while the nitrogen content increased from 4% in SNPs-NH_2_ to 5% in SNPs-DAAQ. This enhancement in the C 1s and N 1s signal intensities could be associated with the incorporation of DAAQ onto the silica material. Further insights were obtained from the deconvolution of the C 1s spectrum of SNPs–DAAQ, which showed the amide bond formation, with the O=C–N group observed at 287.5 eV (Fig. SI8c†), consistent with reported values in the literature^56,57^ Additionally, the N 1s spectrum exhibited two distinct peaks upon deconvolution (Fig. SI8c†): one at 400.5 eV, corresponding to the N–C=O amide bond, and another at 398.4 eV, associated with the –NH_2_ group.^57,58^ These results confirm the successful grafting of DAAQ onto SNPs through a covalent amide bond formation.

Dynamic Light Scattering (DLS) measurements were performed to evaluate the size distribution, dispersibility, and colloidal stability of SNPs-DAAQ in various solvents, including ethanol, nitromethane, acetonitrile, and water (Table SI4 and Fig. SI9†). All samples exhibited two distribution curves: a predominant one with hydrodynamic diameters (*d*_H_) centered around 250 nm, closely aligning with TEM images, and a minor population with *d*_H_ values exceeding 8000 nm. Notably, samples dispersed in water exhibited an additional peak at 782 nm, indicating greater nanoparticle agglomeration in this solvent. This observation was further supported by a higher polydispersity index (PDI) recorded in water compared to the other solvents tested (Table SI4†), suggesting reduced colloidal stability under aqueous conditions.

### Evaluation of photocatalytic activity of SNPs-DAAQ

The catalytic activity of the heterogeneous photocatalyst SNPs–DAAQ was evaluated in three different reaction systems: (i) the reduction of aryl halides (dehalogenation reaction), (ii) the oxidative cross-dehydrogenative coupling (CDC) of *N*-aryl-1,2,3,4-tetrahydroisoquinolines, and (iii) singlet oxygen generation *via* energy transfer.

Dehalogenation of aryl halides is a key transformation in organic chemistry, allowing for the selective removal of halogen atoms to modify molecules and synthesize specific molecular frameworks and access valuable intermediates.^59–61^ This transformation is particularly relevant in the development of pharmaceuticals and natural products, where it can enhance biological properties, as well as for the manufacturing of materials such as polymers and electronic components. Additionally, it contributes to sustainable chemistry by reducing reliance on halogenated compounds.

In the context of catalysis, particularly under photocatalytic conditions, dehalogenation is typically mediated by radical intermediates.^62,63^ Accordingly, the photocatalytic activity of SNPs-DAAQ was evaluated in the model dehalogenation reaction of 4-bromobenzonitrile (1a) to benzonitrile (2a) (Table 1).

**Table 1:** Optimization reaction conditions for the dehalogenation of 4-bromobenzonitrile (1a) to benzonitrile (2a) with homogeneous anthraquinones and heterogeneous SNPs-DAAQ^a^

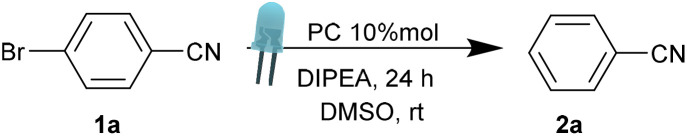					
Reaction condition					
Entry	PC	Catalyst loading (mol%)	Donor	Solvent	Conv. of 1a^b^ (%)
1		10	8 equiv. DIPEA	DMSO	70 (50)
2		10	8 equiv. Et3N	DMSO	61 (33)
3^c^	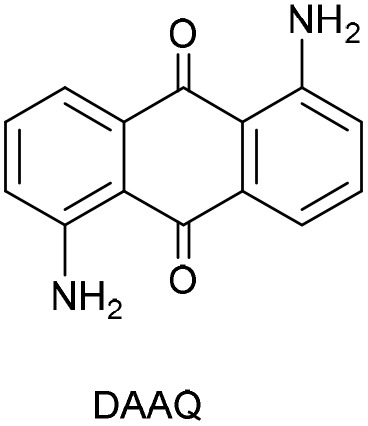	10	8 eq. DIPEA	DMSO	67 (37)
4		20	8 eq. DIPEA	DMSO	73 (39)
5		10	8 eq. DIPEA	DMA	46 (29)
6		10	8 eq. DIPEA	MeCN	9 (<5)
7		10	8 eq. DIPEA	EtOH	0
8^d^		—	8 eq. DIPEA	DMSO	0
9^e^		10	8 eq. DIPEA	DMSO	0
10	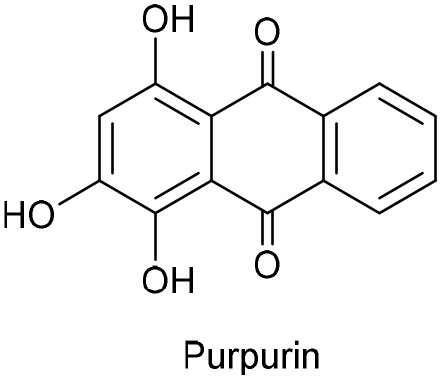	10	8 eq. Et_3_N	DMSO	0
11	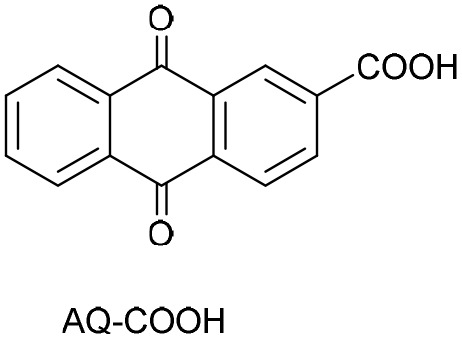	10	8 eq. Et_3_N	DMSO	27 (11)
12	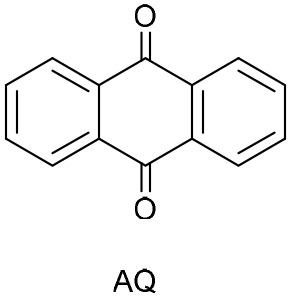	10	8 eq. Et_3_N	DMSO	14 (<5)
13	SNPs-DAAQ	10 (64 mg)	8 eq. DIPEA	DMSO	77 (53)
14^f^			—	DMSO	0
15^e^			8 eq. DIPEA	DMSO	0

a Reaction conditions: 0.1 mmol substrate 1a, the proper amount of PC, 5 mL of solvent, and N_2_ atmosphere, were irradiated for 24 h, employing blue LED 3W lamps.

b Conversion of 1a and yields of 2a were quantified by GC analysis using acetophenone as internal standard. In bracket the yield of 2a was informed.

c 48 h.

d Without PC.

e Without light (dark condition).

f Without DIPEA or electron donor.

As the photocatalytic behavior of DAAQ in this transformation had not been previously reported, preliminary optimization was carried out using the homogeneous catalyst DAAQ. The reaction was carried out with 1a as the substrate, DIPEA as the sacrificial donor, in DMSO under a nitrogen atmosphere, and irradiated with a 3W blue LED light source. After 24 h of irradiation, a 70% conversion of 1a was achieved (entry 1, Table 1).

When Et_3_N was used as the sacrificial electron donor in place of DIPEA, a noticeably lower conversion of 1a was observed (entry 2, Table 1). Extending the irradiation time or increasing the catalyst concentration did not significantly improve the conversion (entries 3 and 4, Table 1). A solvent screening revealed that alternative media such as *N*,*N*-dimethylacetamide (DMA), acetonitrile (MeCN), and ethanol (EtOH) resulted in lower conversion rates of 46%, 9%, and 0%, respectively (entries 5–7, Table 1). Control experiments conducted in the absence of either the catalyst or light showed no conversion of 1a (entries 8 and 9, Table 1).

To further evaluate the structure–activity relationship, other anthraquinones-based photocatalysts bearing electron-withdrawing or electro-donating groups, namely purpurin, 2-carboxylic acid anthraquinone (AQ-COOH), and unsubstituted anthraquinone (AQ) were employed as PC. In all cases, lower conversions were obtained compared to DAAQ (entries 10–12, Table 1), highlighting the enhanced reactivity conferred by the 1,5-diamino substitution. Previous work of König group, evidence the superior catalytic performance of disubstituted anthraquinones with hydroxyl groups on both aromatic rings of anthraquinone backbone.^64^

Under the optimized reaction conditions (entry 1, Table 1), the heterogeneous photocatalyst SNPs-DAAQ achieved a slightly higher conversion of 1a (77%, entry 13, Table 1), demonstrating its effectiveness and stability. Consistent with earlier observations, no conversion was detected in the absence of either DIPEA or light (entries 14 and 15, Table 1), confirming that the transformation proceeds *via* a light-driven photoredox mechanism.

**Fig. 4:**
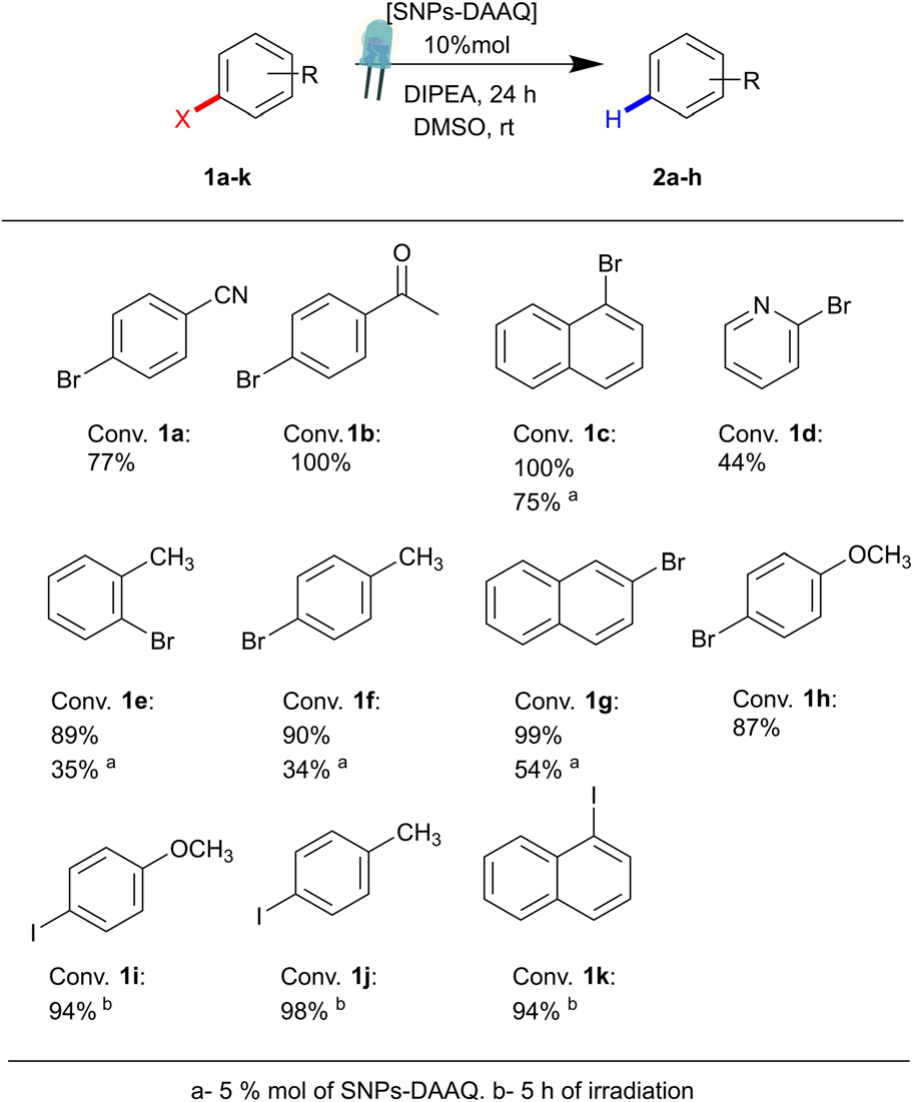
Photocatalytic dehalogenation of aryl bromides and iodides (1a–k) in the presence of SNPs-DAAQ as PC.

Additionally, the scope of the heterogeneous photocatalyst SNPs-DAAQ was evaluated using a series of aryl bromides (1b–1h) and aryl iodides (1i–1k) under optimized reaction conditions. As shown in Fig. 4, the heterogeneous photocatalyst successfully mediated the dehalogenation reactions across structurally diverse substrates, achieving conversions ranging from moderate to excellent (44–100%) after 24 h of irradiation with a 3W blue LED.

**Fig. 5:**
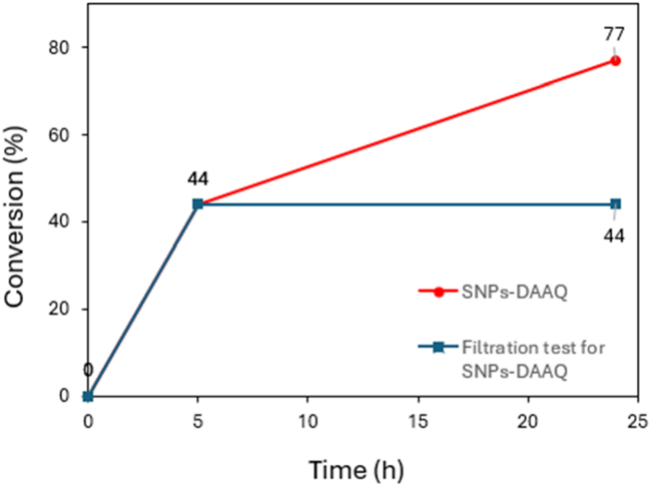
Filtration test in the dehalogenation of 4-bromobenzonitrile (1a) to benzonitrile (2a) in the presence of SNPs-DAAQ.

To further verify the heterogeneous nature of the SNPs-DAAQ catalyst, a filtration test was performed during the debromination of bromobenzonitrile 1a under optimized reaction conditions (Fig. 5). After 5 h of irradiation, the reaction mixture was centrifuged to remove the heterogeneous catalyst, and the supernatant was subsequently allowed to react under continued irradiation for an additional 19 h. No significant product formation was observed following catalyst removal, indicating that the reaction was effectively halted (Fig. 5). This result confirms that the catalytic active species were primarily removed by centrifugation and filtration, supporting the conclusion that SNPs-DAAQ catalyst operates through a heterogeneous pathway.

Based on literature reports, the proposed mechanism for the photocatalytic dehalogenation reaction is illustrated in Scheme SI1 in the ESI.†^21,64^ To determine whether radical intermediates are involved, the reaction was carried out in the presence of 1,1-diphenylethene. The corresponding adduct, 4-(2,2-diphenylvinyl)benzonitrile, was identified by GC-MS (see Section 2.8 of the SI†).

This heterogeneous photocatalyst exhibits a catalytic activity comparable to that of other heterogeneous materials, such us Zr-MOF-OH, QD-CdZn, and Phen-CTF (see Table SI5†).^65–67^ It is important to highlight that this challenging reaction has been scarcely studied using heterogeneous photocatalysst in general.

Since the heterogeneous SNPs-DAAQ photocatalyst exhibited similar catalytic activity to its homogeneous counterpart (DAAQ) in the reductive dehalogenation, we next evaluated its performance in oxidative transformations. The cross-dehydrogenative coupling (CDC) of *N*-aryl-1,2,3,4-tetrahydroisoquinolines is a valuable transformation in organic chemistry due to its ability to form C–C bonds in complex molecules.^68^ This reaction plays a crucial role in the synthesis of N-heterocyclic compounds with unique properties, which is relevant to drug discovery and materials science.^69^ The mild conditions and reagent efficiency of CDC reactions also support sustainable synthetic practices.

In this study, the photocatalytic activity of SNPs-DAAQ was tested in CDC reaction of *N*-phenyl-1,2,3,4-tetrahydroisoquinoline (3a) as the substrate and nitromethane serving as both nucleophile and solvent (Table 2).

**Table 2:** CDC reaction using SNPs-DAAQ as heterogenous PC^a^

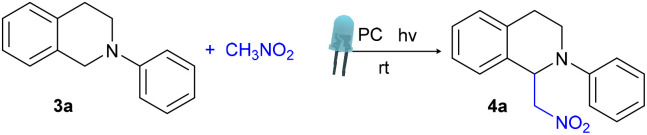				
Entry	Reaction conditions			Yield 4a^b^ [%]
	PC (mol%)	Time (h)	Blue LED	
1	DAAQ (4 mol%)	1.5	48W	75
2	25 mg SNPs-DAAQ (4 mol% DAAQ)	1.5	48W	75 (55)^c^
3	25 mg SNPs-DAAQ (4 mol% DAAQ)	1	48W	60
4	25 mg SNPs-DAAQ (4 mol% DAAQ)	0.5	48W	30
5^d^	—	1	48W	11
6^e^	25 mg SNPs-DAAQ (4 mol% DAAQ)	1.5	—	4

a Reaction conditions: 0.1 mmol substrate 3a, 25 mg SNPs-DAAQ (4 mol% with respect to DAAQ), 1 mL of CH_3_NO_2_ (as the nucleophile and solvent), air atmosphere, were irradiated for the indicated time using 48W blue led lamps.

b Yield of 4a quantified by ^1^H NMR using 4-nitroacetophenone as an internal standard.

c Isolated yield.

d Without PC.

e Without light.

Initially, the homogeneous photocatalyst DAAQ was evaluated. After 1.5 h of irradiation with a 48 W blue LED irradiation, the product 4a was obtained in 75% yield (entry 1, Table 2). Under comparable conditions, the heterogeneous photocatalyst SNPs-DAAQ (25 mg, 4 mol% DAAQ) afforded 4a in 75% yield (55% isolated yield, entry 2, Table 2). When the irradiation time was reduced to 1 and 0.5 h, the yield of 4a decreased to 60% and 30%, respectively (entries 3 and 4, Table 2). Control reactions performed in the absence of either photocatalyst and light afforded 4a in 11% and 4% yields, respectively (entries 5 and 6, Table 2), confirming the necessity of both components for effective catalysis.

Following this initial success, we evaluated the substrate scope of the CDC reaction. A variety of substituted 1,2,3,4-tetrahydroisoquinoline derivatives were effectively coupled with nitromethane, affording the corresponding products 4a–g in good to excellent yields (Table 3). The reaction exhibited broad functional group tolerance. Additionally, the use of an alternative nucleophile, nitropropane, led to the formation of the desired product 4h with a 65% yield.

**Table 3:** Substrate scope evaluation in CDC reaction in the presence of heterogeneous SNPs-DAAQ^a^^,^^b^^,^^c^

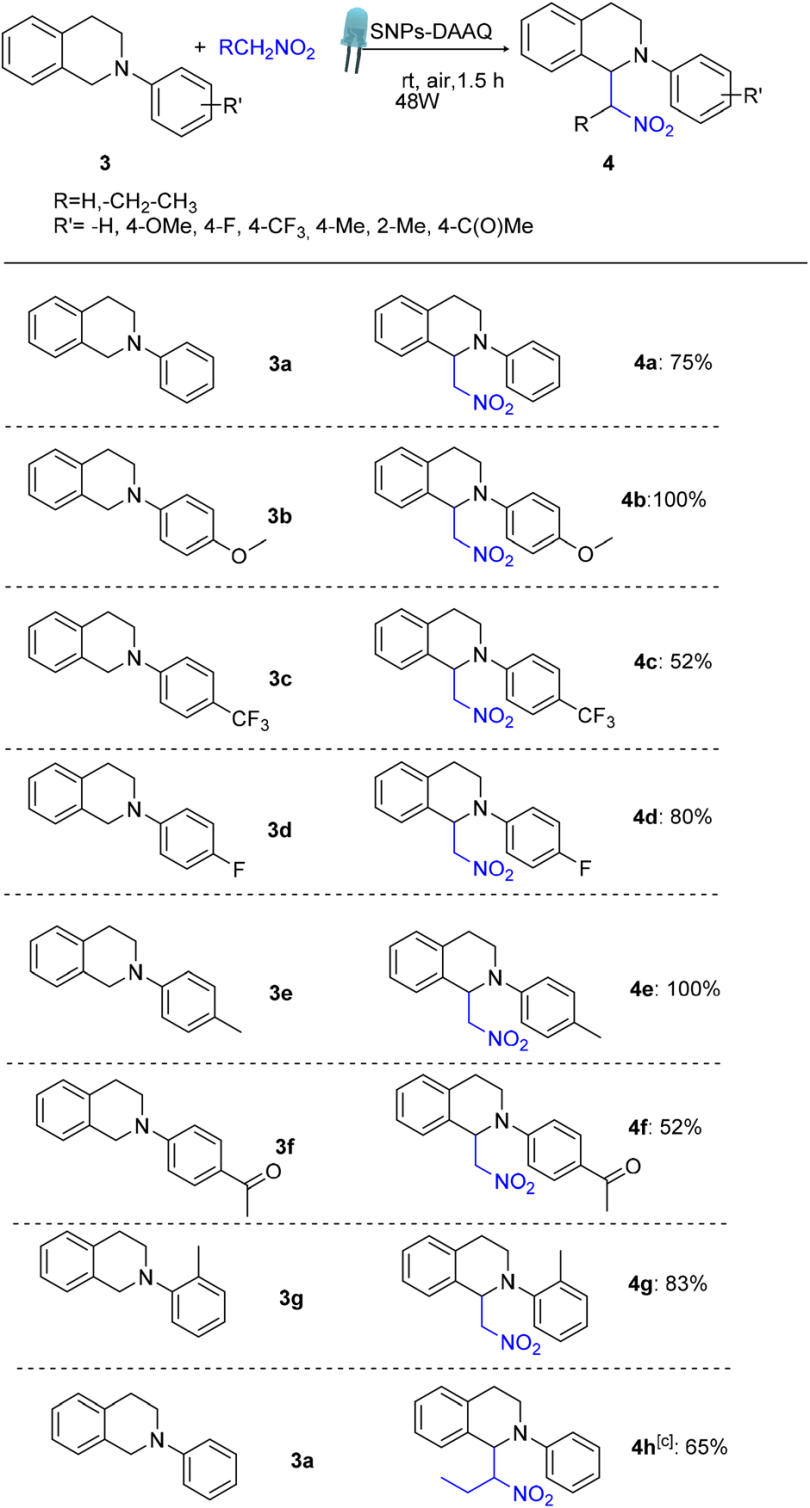

a Reaction conditions: 0.1 mmol substrate 3, 25 mg SNPs-DAAQ (4 mol% with respect to DAAQ), 1 mL of CH_3_NO_2_ (as the nucleophile and solvent), air atmosphere, were irradiated for the indicated time using 48W blue led lamps.

b Yield of 4a–h quantified by ^1^H NMR using 4-nitroacetophenone as an internal standard.

c Reaction done using nitropropane as the nucleophile and solvent.

**Fig. 6:**
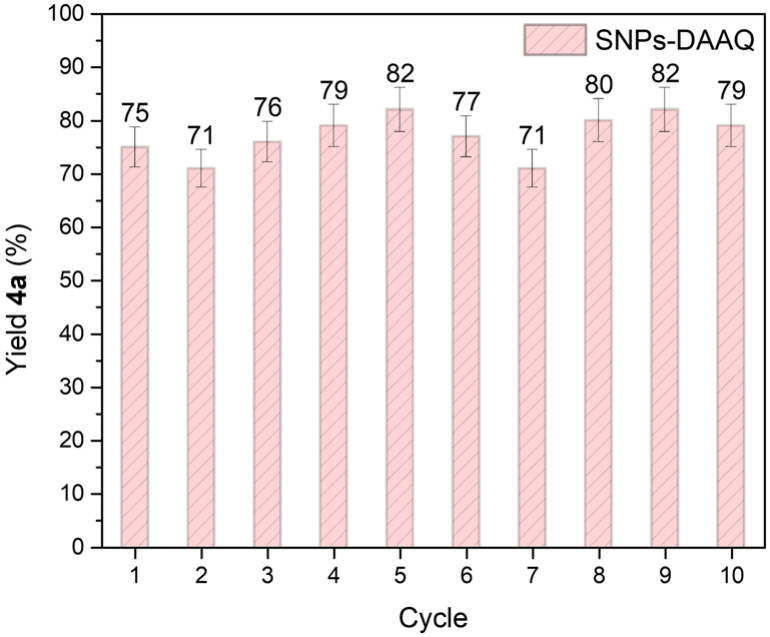
Recycling experiment in CDC reaction of 3a and nitromethane in presence of SNPs-DAAQ. The values correspond to the average yield from two independent catalytic runs.

To assess the reusability of the heterogeneous photocatalyst SNPs-DAAQ, recycling experiments were conducted for the oxidation of tetrahydroisoquinoline 3a in the CDC coupling reaction as a model system (Fig. 6). The results demonstrate that the photocatalyst remained effective over at least 10 consecutive reaction cycles, without any noticeable loss in activity. Moreover, photostability tests of both DAAQ and SNPs-DAAQ were performed by irradiating photocatalyst dispersion in DMSO for 24 h (Fig. SI15 and Table SI6†). Notably, the incorporation of DAAQ onto the SNPs significantly enhanced the photostability of the heterogeneous catalyst.

Post-reaction characterization by TEM after ten reaction cycles, showed that the recycled SNPs-DAAQ maintained its original size and morphology (Fig. SI10a and b†). Furthermore, the FT-IR spectrum of the recycled material (Fig. SI10c†) exhibited the characteristic absorption bands of DAAQ at 1658 cm^−1^, 1330 cm^−1^, 1295 cm^−1^ and 750 cm^−1^; confirming the presence of the photocatalyst in the nanomaterial after repeated use.

Based on reported literature, a mechanistic proposal is depicted in Scheme SI2 (Section SI 6.3).†^68,70^ A plausible mechanism for the CDC reaction involves the generation O_2_˙^−^, which subsequently leads to the formation of hydrogen peroxide (H_2_O_2_) and the iminium intermediate (5, Scheme SI2†) in the reaction medium. The iminium 5 is a key intermediate in CDC reaction. To experimentally confirm the formation of these species, control experiments were performed. First, the PC (SNPs-DAAQ), substrate 3a, and nitromethane were irradiated under an O_2_ atmosphere for 1.5 h. Following this, KI/AcOH solution and starch were added, resulting in the formation of the blue [starch-I_2_] complex, confirming the presence of H_2_O_2_ in the reaction mixture (see ESI, Section SI6.1†).^48^ Additionally, to confirm the formation of intermediate cation 5, a reaction was carried out between 3a and SNPs-DAAQ in isopropanol, in the absence of nitromethane. After 1.5 h of irradiation, ^1^H NMR analysis revealed the presence of iminium 5 in the reaction medium (Section SI6.2†).^70–72^

**Fig. 7:**
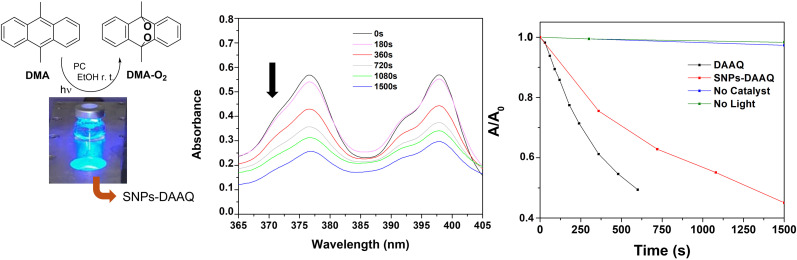
Photocatalytic oxidation of DMA (6 × 10^−5^ M) to 9,10-endoperoxide. Decrease in absorbance of DMA *vs.* irradiation time using SNPs-DAAQ (10 mol%). Irradiation conditions: Blue LED 3W lamp and EtOH as the solvent.

Furthermore, the catalytic activity of SNPs-DAAQ was evaluated in the catalytic photooxidation of 9,10-dimethylanthracene (DMA), as illustrated in Fig. 7. DMA is a typical singlet oxygen quencher and serves as a model substrate to assess the catalytic activity of PCs through energy transfer reactions.^73^ Under photooxidation conditions, DMA reacts with singlet oxygen *via* a [4 + 2] cycloaddition, forming a 9,10-endoperoxide product. The reaction progress was monitored by UV-visible spectroscopy tracking the absorbance of DMA over time. Employing SNPs-DAAQ (10 mol% DAAQ) and 3W blue LED light source, 85% of substrate conversion was achieved after 1500 seconds, observing a decrease in DMA absorbance (Fig. 7). Homogeneous DAAQ (10 mol%) achieved a 92% conversion of DMA after 600 seconds (see Fig. SI13†). Furthermore, the formation of the DMA-O_2_ endoperoxide was confirmed by both ^1^H and ^13^C NMR spectroscopy. Quantitative analysis revealed 85% conversion of DMA and an 80% yield of DMA-O_2_ (see Fig. SI14 and Section 10 of the ESI†). Control experiment performed in the absence of the PC showed negligible substrate conversion, confirming that the SNPs-DAAQ material effectively catalyzed the generation of singlet oxygen under light irradiation (Fig. 7).

### Evaluation of antimicrobial activity of SNPs-DAAQ

Having established that both DAAQ and SNPs-DAAQ can generate reactive oxygen species (ROS) with suitable photochemical properties for antimicrobial photodynamic therapy (PDT), their antimicrobial activity was evaluated against a Gram-positive (G+) bacterial strain, *Staphylococcus aureu*s ATCC 29213.

**Fig. 8:**
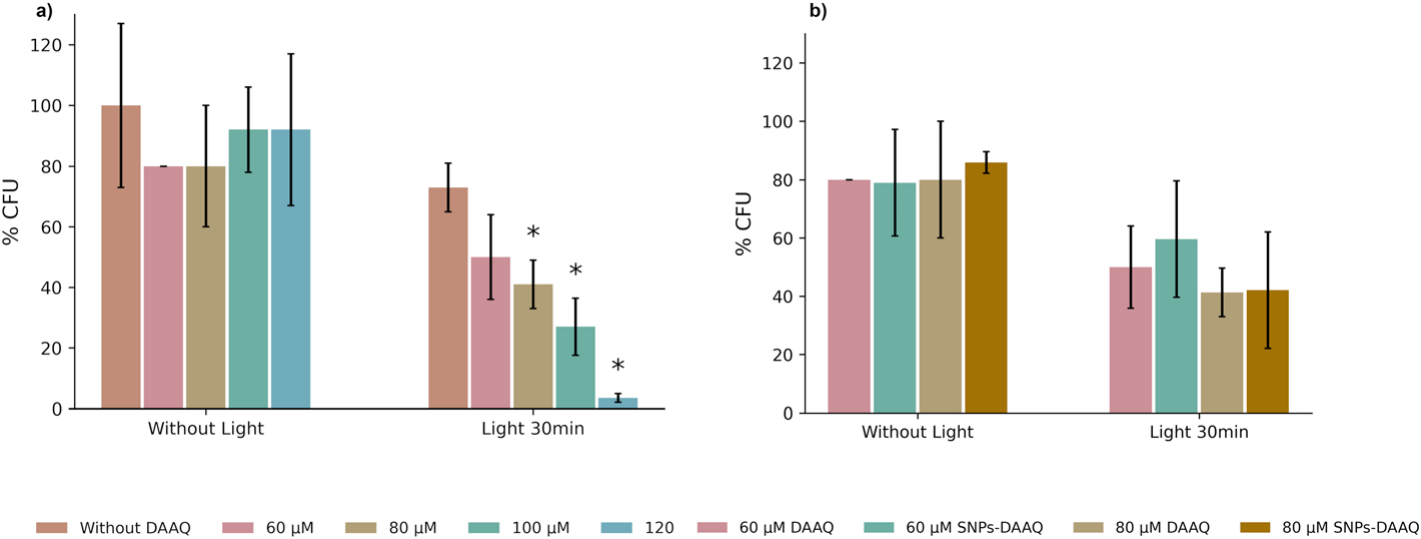
(a) Inactivation of *S. aureus* ATCC 29213 mediated by DAAQ without light (left) and with blue LED (right) in PBS. DAAQ concentrations tested were 60 μM, 80 μM, 100 μM, and 120 μM. The bar graphs show the percentage of CFU mL^−1^ (average of triplicate experiments) surviving PDT, with respect to the initial inoculum that was not exposed to blue LED light and without DAAQ (control). (b) Comparative Inactivation of *S. aureus* ATCC 29213 mediated by DAAQ 60 μM and 80 μM and SNPs-DAAQ 60 μM and 80 μM without light (left) and with blue LED (right) in PBS. The bars represented DSE- *p < 0.05.

The first step was to determine whether DAAQ exhibited intrinsically toxicity in the absence of light irradiation. Antibacterial efficacy was quantified by measuring the percentage of Colony-Forming Unit inhibition (%CFU), which reflects a compound's ability to inhibit bacterial growth by comparing CFU counts in treated samples to those of untreated controls. In this case, the %CFU was evaluated for solutions containing the inoculum and DAAQ at concentrations of 60 μM, 80 μM, 100 μM, and 120 μM, respectively. The results indicated that, under dark conditions, DAAQ induced only slight bacterial inhibition, with %CFU values ranging from 8% to 20%, depending on the concentration (Fig. 8a), indicating low inherent toxicity in the absence of light.

Subsequently, the PDT assay was performed, in which the bacteria were incubated in sterile flasks with different concentrations of DAAQ, followed by irradiation for 30 minutes using a 3W blue LED lamp. As shown in Fig. 8a, a clear inverse correlation was observed between DAAQ concentration and %CFU, indicating increased microbial inactivation with higher photosensitizer doses. This enhanced antibacterial effect is attributed to the greater amounts of ROS at higher DAAQ concentrations, which intensifies oxidative stress on G+ bacteria cells. In this context, %CFU values of 50, 41, 27, and 3 were obtained for DAAQ-concentrations of 60 μM, 80 μM, 100 μM, and 120 μM, respectively. The 26% bacterial inhibition growth observed without the presence of a photosensitizer can be attributed to the photolytic effect of blue LED light, consistent with previous studies that report microbial inactivation induced by UV and visible radiation, resulting from direct damage to key biomolecules and the generation of endogenous reactive oxygen species.^74^

Finally, the antibacterial activity of SNP–DAAQ was evaluated by determining the %CFU at concentrations of 60 μM and 80 μM. The observed inhibition levels were comparable to those obtaining with of DAAQ in homogeneous solution at the same concentration (50% *vs.* 58% CFU for 60 μM and 41% *vs.* 42% CFU for 80 μM; Fig. 8b). These results suggest that the immobilization of DAAQ onto silica does not affect its ability to inhibit bacterial growth, further supporting the potential of SNPs-DAAQ as an effective heterogeneous photosensitizer for antimicrobial applications. We are currently studying the incorporation of SNPs-DAAQ into liposomes and gels to increase their bioavailability. Moreover, further studies are being carried out on the use of heterogeneous systems for the disinfection of water contaminated with pathogenic bacteria, aiming at the development of efficient decontamination devices.

## Experimental

### Materials and methods

All chemicals were reagent grade and were used as received from the manufacturer. 1,5-Diaminoanthraquinone (DAAQ); ammonium hydroxide (NH_4_OH, 28% w/w); tetraethyl orthosilicate (TEOS), (3-aminopropyl)-triethoxysilane (APTES), *N*,*N*′-dicyclohexylcarbodiimide (DCC), 1-hydroxibenzotriazole (HOBt), succinic acid, 1,2,3,4-tetrahydroisoquinoline, iodobenzene, 4-iodoacetophenone, 1-fluoro-4-iodobenzene, 1-iodo-4-methoxybenzene, 1-iodo-4-(trifluoromethyl)benzene, 1-iodo-2-methylbenzene, 1-iodo-4-methylbenzene, palladium(ii) acetate, potassium-*t*-butoxide, 2,2′-bis(diphenylphosphino)-1,1′-binaphthyl, nitromethane, 1-nitropropane, NaOH, dimethylanthracene (DMA), bromobenzonitrile, benzonitrile, bromoacetophenone, acetophenone, 1-bromonaphtalene, naphthalene, 2-bromopyridine, pyridine, 1-bromo-2-methylbenzene, 1-bromo-4-methylbenzene, 2-bromonaphtalene, 1-bromo-4-methoxybenzene, 1-iodo-4-methoxybenzene, anisole, 1-iodo-4-methylbenzene, 1-iodonaphtalene, 1,1-diphenylethene, *N*,*N*-diisopropylethylamine (DIPEA), benzophenone, *p*-nitroacetophenone. Ethanol 98% (EtOH), acetonitrile (MeCN), tetrahydrofurane (THF), dimethyl sulfoxide (DMSO), *N*,*N*-dimethylacetamide (DMA), and anhydrous Na_2_SO_4_ were used as received. Hexane, acetone and ethyl acetate solvents were analytical grade and distilled before use and toluene was distilled and dried under molecular sieves (3 Å). Milli-Q-Millipore water was employed in all the experiments. Silica gel (0.063–0.200 mm) was used in column chromatography.

The nanomaterials were characterized by Transmission Electron Microscopy (TEM) using a JEM-Jeol 1120 microscope operating at 80 kV. SEM microscopy was performed in a SEM – Zeiss Sigma 360. The UV-vis measurements were carried out in a Shimadzu UV-2101 PC. Solid state fluorescence, and fluorescence in solutions were performed in Horiba Nanolog. FT-IR spectra were collected on an infrared microscope Nicolet iN10, Thermo Scientifics, USA. DLS measurements were performed by using a Delsa Nano C instrument, Beckman Coulter, Osaka, Jp. A commercial Thermo Scientific K-Alpha X-ray photoelectron spectrometer (XPS) system, equipped with a hemispherical energy analyzer and a monochromated X-ray source was used for surveying the photoemission spectra. Solid-state nuclear magnetic resonance (ss-NMR) experimental data were acquired with a Bruker Avance-III HD spectrometer equipped with a 14.1 T narrow bore magnet operating at Larmor frequencies of 600.09, 150.91 and 119.21 MHz for ^1^H, ^13^C and ^29^Si, respectively.

^1^H NMR and ^13^C NMR were conducted on a High-Resolution Spectrometer Bruker Advance 400, in CDCl_3_ as solvent. Gas chromatographic analyses were performed on a gas chromatograph with a flame ionization detector and equipped with the following column: VF-5 30 m × 0.20 mm × 0.25 μm column. Gas chromatographic/mass spectrometer analyses were carried out on a GC/MS QP 5050 spectrometer equipped with a VF-5ms, 30 m × 0.25 mm × 0.25 μm column.

### Immobilization of 1,5-diaminoanthraquinone (DAAQ) onto SNPs-COOH^75^

In a 10 mL Schlenk tube equipped with a magnetic stir bar and under a nitrogen atmosphere, 200 mg of SNPs-COOH, 65 mg of HOBt (5 eq.), 87 mg of DCC (5 eq.), and 4 mL of acetone were added. The reaction mixture was heated under reflux for 1 h. After cooling to room temperature, 20 mg of DAAQ (1 eq.) were added under a nitrogen atmosphere, and the mixture was stirred for 24 h. The resulting SNPs-DAAQ were centrifuged at 3500 rpm for 8 min, then suspended in THF, sonicated for 5 min, and centrifuged again for 8 min. The SNPs-DAAQ were washed with acetone, acetonitrile (MeCN), and ethanol three times, following the same procedure each time. Finally, the SNPs-DAAQ were dried in air at room temperature for 24 h. After this procedure, 180 mg of SNPs-DAAQ were recovered.

### Procedure for the photocatalytic dehalogenation of aryl halides^64^

In a 10 mL scintillation vial equipped with a magnetic stir bar, 0.1 mmol of the substrate (1a–k), SNPs-DAAQ (10 mol% DAAQ), and 5 mL of DMSO were added. The vial was sealed with a Teflon plug, and nitrogen was passed through for 5 min to saturate the solution. Next, using a syringe, DIPEA (8 equiv) was added. The vial was then irradiated with a 3 W blue LED under vigorous magnetic stirring for 24 h. After this time, 1 mL of a standard solution (2 mg mL^−1^) was added to the reaction mixture. The mixture was then centrifuged at 3500 rpm for 8 min, and the supernatant was analyzed by GC using acetophenone or benzophenone as the internal standard.

### Photocatalyzed CDC reaction with SNPs-DAAQ catalysts

*N*-aryl-1,2,3,4-tetrahydroisoquinoline (0.1 mmol), SNPs-DAAQ (4 mol% with respect to DAAQ) and 1 mL of nitromethane were charged in a reaction glass tube with magnetic stirring bar. The reaction mixture was stirred under blue LED (465 nm, 48W, Fig. SI2†) irradiation at room temperature for 1.5 h under air atmosphere. After 1.5 h, the solvent was removed under vacuum, and the product was quantified by ^1^H-NMR, with *p*-nitroacetophenone as internal standard. After quantification, the crude product was purified by column chromatography (Hexane:EtOAc).

### Recycling test in CDC reaction

*N*-phenyl-1,2,3,4-tetrahydroisoquinoline (3a, 0.1 mmol), SNPs-DAAQ (4 mol% with respect to DAAQ) and 1 mL of nitromethane were placed in a reaction glass tube equipped with a magnetic stirring bar. The reaction mixture was stirred under blue LED irradiation (465 nm, 48W) at room temperature for 1.5 h under air atmosphere. After the reaction time, the crude was centrifugated and the solid catalyst was separated and dry under vacuum (this solid was stored for reuse in the next reaction cycle). The supernatant was evaporated, and the product was quantified by ^1^H-NMR, using *p*-nitroacetophenone as internal standard.

### Photooxidation of DMA

5 mL of a 9,10-dimethylanthracene (DMA, 6 × 10^−5^ M) solution in EtOH and 10 mol% of the catalyst were added to a vial containing a stir bar. The reaction mixture was then irradiated with a blue LED (3W) lamp while stirring at room temperature until the DMA was fully consumed. UV-vis spectra were recorded at various irradiation times. The decrease in absorbance at *λ* = 379 nm, corresponding to DMA, was used to monitor the oxidation process.

### Photodynamic inactivation: *in vitro* phototoxicity assays

An aliquot of the bacterial suspension (1 mL) was placed in a test tube together with 1 mL of different concentrations of the PC solutions. The solutions of the dyes were prepared from a stock solution of DAAQ in DMSO and SNPs-DAAQ and then diluted with PBS to obtain 60–120 μM solutions.

*S. aureus* ATCC 29213 was pre-incubated for 15 min at different concentrations of each PC in a sterility for this assay. To perform the *in vitro* phototoxicity assays, the test tubes were illuminated with a blue LED lamp (3W) for 30 min. After that, the aliquots were removed, 10-fold serially diluted in PBS, cultured in Petri dishes containing TSA and incubated overnight at 37 °C. Colony Forming Units (CFU) were counted, and survival fractions were determined in triplicate by the drop-plate technique for bacterial enumeration according to Naghili *et al.*^76^ The values were expressed as the mean. For the control samples, the same set-up was used in the absence of LED light to evaluate the toxicity of DAAQ and SNPs-DAAQ.

## Conclusions

In this study, we successfully developed a new heterogeneous photocatalyst by covalently immobilizing 1,5-diaminoanthraquinone (DAAQ) onto functionalized silica nanoparticles (SNPs). The photocatalyst was comprehensively characterized using a variety of microscopic, spectroscopic, and surface analysis techniques, confirming the successful anchoring of DAAQ onto the silica support. The resulting photocatalyst, SNPs-DAAQ, exhibited significant potential in organic synthesis, and exhibited excellent performance across a range of photocatalytic applications, including cross-dehydrogenative coupling (CDC) reaction, dehalogenation of aryl halides, and singlet oxygen generation *via* energy transfer mechanisms. Notably, the SNPs-DAAQ catalyst exhibited excellent recyclability, maintaining its structural integrity and catalytic activity over multiple reaction cycles. Additionally, SNPs-DAAQ displayed promising antimicrobial activity under visible light irradiation, highlighting its potential for use in photodynamic antimicrobial therapy. These findings position SNPs-DAAQ as a versatile and sustainable photocatalyst with broad utility in synthetic organic chemistry and biomedical fields.

## Author contributions

The manuscript was written through contributions of all authors. All authors have given approval to the final version of the manuscript.

## Conflicts of interest

There are no conflicts to declare.

## Supplementary Material

RA-015-D5RA03539B-s001

## Data Availability

The data supporting this article have been included as part of the ESI.†

## References

[cit1] Shaw M. H., Twilton J., MacMillan D. W. C. (2016). J. Org. Chem..

[cit2] Xi Y., Yi H., Lei A. (2013). Org. Biomol. Chem..

[cit3] Narayanam J. M. R., Stephenson C. R. J. (2011). Chem. Soc. Rev..

[cit4] Xuan J., Xiao W.-J. (2012). Angew. Chem., Int. Ed..

[cit5] Grudzień K., Zlobin A., Zadworny J., Rybicka-Jasińska K., Sadowski B. (2024). Org. Chem. Front..

[cit6] Mohamadpour F., Amani A. M. (2024). RSC Adv..

[cit7] Bell J. D., Murphy J. A. (2021). Chem. Soc. Rev..

[cit8] Koike T., Akita M. (2014). Inorg. Chem. Front..

[cit9] Shon J. H., Kim D., Rathnayake M. D., Sittel S., Weaver J., Teets T. S. (2021). Chem. Sci..

[cit10] Romero N. A., Nicewicz D. A. (2016). Chem. Rev..

[cit11] Hari D. P., König B. (2014). Chem. Commun..

[cit12] Sui X., Dang H. T., Porey A., Trevino R., Das A., Fremin S. O., Hughes W. B., Thompson W. T., Dhakal S. K., Arman H. D., Larionov O. V. (2024). Chem. Sci..

[cit13] Singh P. P., Singh J., Srivastava V. (2023). RSC Adv..

[cit14] Tlili A., Lakhdar S. (2021). Angew. Chem., Int. Ed..

[cit15] Srivastava V., Singh P. K., Srivastava A., Singh P. P. (2021). RSC Adv..

[cit16] Insińska-Rak M., Sikorski M., Wolnicka-Glubisz A. (2023). Cells.

[cit17] Chen W., Chen J. J., Lu R., Qian C., Li W. W., Yu H. Q. (2014). Bioelectrochemistry.

[cit18] Sharma S., Sharma A. (2019). Org. Biomol. Chem..

[cit19] Srivastava A., Singh P. K., Ali A., Singh P. P., Srivastava V. (2020). RSC Adv..

[cit20] Yoshioka E., Kohtani S., Jichu T., Fukazawa T., Nagai T., Kawashima A., Takemoto Y., Miyabe H. (2016). J. Org. Chem..

[cit21] Chen C. X., Yang S. S., Pang J. W., He L., Zang Y. N., Ding L., Ren N. Q., Ding J. (2024). Environ. Sci. Ecotechnology.

[cit22] Cervantes-González J., Vosburg D. A., Mora-Rodriguez S. E., Vázquez M. A., Zepeda L. G., Villegas Gómez C., Lagunas-Rivera S. (2020). ChemCatChem.

[cit23] Srivatsavoy V. J. P., Venkataraman B., Periasamy N. (1992). Proc.–Indian Acad. Sci., Chem. Sci..

[cit24] Rusch F., Unkel L. N., Alpers D., Hoffmann F., Brasholz M. (2015). Chem.–Eur. J..

[cit25] Unkel L. N., Malcherek S., Schendera E., Hoffmann F., Rehbein J., Brasholz M. (2019). Adv. Synth. Catal..

[cit26] von Drathen T., Hoffmann F., Brasholz M. (2018). Chem.–Eur. J..

[cit27] Wang G., Hill N. S., Zhu D., Xiao P., Coote M. L., Stenzel M. H. (2019). ACS Appl. Polym. Mater..

[cit28] Savateev A., Antonietti M. (2018). ACS Catal..

[cit29] Mora-Rodríguez S. E., Camacho-Ramírez A., Cervantes-González J., Vázquez M. A., Cervantes-Jauregui J. A., Feliciano A., Guerra-Contreras A., Lagunas-Rivera S. (2022). Org. Chem. Front..

[cit30] Martínez-Aguirre M., Serrano E., Ezquerro C., Lalinde E., Berenguer J. R., García-Martínez J., Rodríguez M. A. (2023). Catal. Today.

[cit31] Caminos D. A., Rimondino G. N., Gatica E., Massad W. A., Argüello J. E. (2023). ACS Omega.

[cit32] Albiter E., Alfaro S., Valenzuela M. A. (2015). Photochem. Photobiol. Sci..

[cit33] Lancel M., Gomez C., Port M., Amara Z. (2021). Front. Chem. Eng..

[cit34] Epelde-Elezcano N., Prieto-Montero R., Martínez-Martínez V., Ortiz M. J., Prieto-Castañeda A., Peña-Cabrera E., Belmonte-Vázquez J. L., López-Arbeloa I., Brown R., Lacombe S. (2017). Phys. Chem. Chem. Phys..

[cit35] Pourshiani O., Romero Z., Ganji N., Herrendorf T., Seifert A., Demchenko A., Prosenc M. H., Kleist W., Kopnarski M., Karimi B., Thiel W. R. (2024). ChemCatChem.

[cit36] Terra J. C. S., Desgranges A., Amara Z., Moores A. (2023). Catal. Today.

[cit37] de Souza Oliveira R. C., Corrêa R. J., Teixeira R. S. P., Queiroz D. D., da Silva Souza R., Garden S. J., de Lucas N. C., Pereira M. D., Bello Forero J. S., Romani E. C., Ribeiro E. S. (2016). J. Photochem. Photobiol., B.

[cit38] Teixeira R. I., De Lucas N. C., Garden S. J., Lanterna A. E., Scaiano J. C. (2020). Catal. Sci. Technol..

[cit39] Ali H., Ahmed I., Robertson K., Lanterna A. E. (2024). Org. Process Res. Dev..

[cit40] Špačková J., Svobodová E., Hartman T., Stibor I., Kopecká J., Cibulková J., Chudoba J., Cibulka R. (2017). ChemCatChem.

[cit41] Mendoza C., Emmanuel N., Páez C. A., Dreesen L., Monbaliu J. C. M., Heinrichs B. (2018). ChemPhotoChem.

[cit42] Kurfiřt M., Špačková J., Svobodová E., Cibulka R. (2018). Monatsh. Chem..

[cit43] Mahmoud N., Awassa J., Toufaily J., Lebeau B., Daou T. J., Cormier M., Goddard J. P. (2023). Molecules.

[cit44] Guo S., Zhang H., Huang L., Guo Z., Xiong G., Zhao J. (2013). Chem. Commun..

[cit45] Soria-Castro S. M., Lebeau B., Cormier M., Neunlist S., Daou T. J., Goddard J. P. (2020). Eur. J. Org Chem..

[cit46] Tambosco B., Segura K., Seyrig C., Cabrera D., Port M., Ferroud C., Amara Z. (2018). ACS Catal..

[cit47] Martin M. G., Lázaro-Martínez J. M., Martín S. E., Uberman P. M., Budén M. E. (2024). Chem.–Eur. J..

[cit48] Martin M. G., Clavero L. R., Martín S. E., Uberman P. M., Budén M. E. (2025). Eur. J. Org. Chem..

[cit49] Qun T., Zhou T., Hao J., Wang C., Zhang K., Xu J., Wang X., Zhou W. (2023). RSC Med. Chem..

[cit50] Comini L. R., Núñez Montoya S. C., Páez P. L., Argüello G. A., Albesa I., Cabrera J. L. (2011). J. Photochem. Photobiol., B.

[cit51] Raghuveer D., Pai V. V., Murali T. S., Nayak R. (2023). ChemistrySelect.

[cit52] Zhang L., Chen P., Xu Y., Nie W., Zhou Y. (2020). Appl. Catal., B.

[cit53] Liu N., Sun G., Zhu J. (2011). J. Mater. Chem..

[cit54] Cardoso V., Rittmeyer T., Correa R. J., Brêda G. C., Almeida R. V., Simões G., de França B. M., de Azevedo P. N., Bello Forero J. S. (2019). Dyes Pigm..

[cit55] Peixoto A. F., Silva S. M., Costa P., Santos A. C., Valentim B., Lázaro-Martínez J. M., Freire C. (2020). Catal. Today.

[cit56] Yao M., Sano H., Ando H. (2023). Polymers.

[cit57] Karan S., Jiang Z., Livingston A. G. (2015). Science.

[cit58] Jansen R. J. J., van Bekkum H. (1995). Carbon.

[cit59] Lan J., Chen R., Duo F., Hu M., Lu X. (2022). Molecules.

[cit60] Shirakawa E., Hayashi T. (2012). Chem. Lett..

[cit61] Ghosh I., Ghosh T., Bardagi J. I., König B. (2014). Science.

[cit62] Fagnoni M., Dondi D., Ravelli D., Albini A. (2007). Chem. Rev..

[cit63] Ravelli D., Protti S., Fagnoni M. (2016). Chem. Rev..

[cit64] Bardagi J. I., Ghosh I., Schmalzbauer M., Ghosh T., König B. (2018). Eur. J. Org Chem..

[cit65] Zhao Z., Liu M., Zhou K., Shen Y., Hong L., Bao Z., Yang Q., Ren Q., Zhang Z. (2023). Catal. Sci. Technol..

[cit66] Pal A., Ghosh I., Sapra S., König B. (2017). Chem. Mater..

[cit67] López-Magano A., Salaverri N., Marzo L., Mas-Ballesté R., Alemán J. (2022). Appl. Catal., B.

[cit68] Bagdi A. K., Rahman M., Bhattacherjee D., Zyryanov G. V., Ghosh S., Chupakhin O. N., Hajra A. (2020). Green Chem..

[cit69] Tian T., Li Z., Li C. J. (2021). Green Chem..

[cit70] Bartling H., Eisenhofer A., Konig B., Gschwind R. M. (2016). J. Am. Chem. Soc..

[cit71] Freeman D. B., Furst L., Condie A. G., Stephenson C. R. J. (2012). Org. Lett..

[cit72] Liu Q., Li Y., Zhang H., Chen B., Tung C., Wu L. (2012). Chem.–Eur. J..

[cit73] Schweitzer C., Schmidt R. (2003). Chem. Rev..

[cit74] Niu L., Wu Z., Yang L., Wang Y., Xiang Q., Bai Y. (2021). J. Food. Prot..

[cit75] Manne S. R., Luna O., Acosta G. A., Royo M., El-Faham A., Orosz G., De La Torre B. G., Albericio F. (2021). Org. Lett..

[cit76] Naghili H., Tajik H., Mardani K., Razavi Rouhani S. M., Ehsani A., Zare P. (2013). Vet. Res. Forum..

