# Supplementary material for: Design of aminoanthraquinone-based heterogeneous photocatalysts for visible-light-driven reactions and antibacterial applications
Source: RSC Adv. 2025 Jul 11;15(30):24393–405. doi: 10.1039/d5ra03539b (PMC12246944; doi:10.1039/d5ra03539b)
Supplement: RA-015-D5RA03539B-s001 [file RA-015-D5RA03539B-s001.pdf]

This journal is © The Royal Society of Chemistry 2025

Table of contents

**1. Materials and equipment**	S2
**2. General experimental methods**	S4
**2.1. Synthesis of SiO_2 nanoparticles functionalized with amino group ($SNPs-NH_2$)**	S4
**2.2. Immobilization of Succinic Acid onto $SNPs-NH_2$**	S4
**2.3. Immobilization of 1,5-Diaminoanthraquinone (DAAQ) onto SNPs-COOH**	S4
**2.4. Synthesis of *N*-Aryl-1,2,3,4-tetrahydroisoquinolines (3a-g)**	S5
**2.5. Photocatalyzed Aza-Henry reaction with SNPs-DAAQ catalysts**	S5
**2.6. Recycling Test**	S5
**2.7. Procedure for the photocatalytic dehalogenation of aryl halides**	S6
**2.8. Photocatalytic dehalogenation of 1a in the presence of 1,1-diphenylethene: Procedure for radical trapping.**	S6
**2.9. Photooxidation of DMA**	S6
**3. Photochemical reactors**	S7
**4. Characterization of SNPs materials**	S8
**4.1. Transmission Electron Microscopy (TEM) and Scanning electron microscopy (SEM)**	S8
**4.2. UV-vis**	S8
**4.3. FT-IR analyses**	S9
**4.4. *ss*-NMR analysis**	S10
**4.5. XPS measurements**	S12
**4.6. DLS measurements**	S13
**4.7. Characterization of the Recycled Nanomaterial**	S15
**5. Dehalogenation of aryl halides**	S16
**5.1. Plausible reaction mechanism for dehalogenation reaction of aryl halides**	S16
**5.2. Comparison of heterogeneous photocatalyst in dehalogenation reaction of aryl halides**	S17
**6. Detection of intermediates in the Aza-Henry reaction (CDC reaction)**	S18
**6.1. H_2O_2 determination**	S18
**6.2. Iminium intermediate identification**	S19
**6.3. Plausible reaction mechanism for CDC Reaction**	S20
**7. Photocatalytic oxidation of dimethylanthracene (DMA)**	S21
**8. Photodynamic inactivation**	S22
**8.1. Bacterial culture**	S22
**8.2. *In vitro* phototoxicity assays**	S23

**9. Photostability of homogeneous and heterogeneous photocatalysts**	S23
**10. Characterization Data of Compounds 2a-h, 3a-g, 4a-h and DMA-O_2**	S25
**11. NMR spectra of compounds 2a-h, 3a-g, 4a-h and DMA-O_2**	S34
**12. References**	S73

1. Materials and equipment

All chemicals were reagent grade and were used as received from the manufacturer.1,5-Diaminoanthraquinone (DAAQ); ammonium hydroxide (NH_4OH, 28 % w/w); tetraethyl orthosilicate (TEOS), (3-aminopropyl)-triethoxysilane (APTES), *N,N´*-dicyclohexylcarbodiimide (DCC), 1-hydroxibenzotriazole (HOBt), succinic acid, 1,2,3,4-tetrahydroisoquinoline, iodobenzene, 4-iodoacetophenone, 1-fluoro-4-iodobenzene, 1-iodo-4-methoxybenzene, 1-iodo-4-(trifluoromethyl)benzene, 1-iodo-2-methylbenzene, 1-iodo-4-methylbenzene, Palladium(II) acetate, potassium-*t*-butoxide, 2,2'-bis(diphenylphosphino)-1,1'-binaphthyl, nitromethane, 1-nitropropane, NaOH, dimethylanthracene (DMA), bromobenzonitrile, benzonitrile, bromoacetophenone, acetophenone, 1-bromonaphtalene, naphthalene, 2-bromopyridine, pyridine, 1-bromo-2-methylbenzene, 1-bromo-4-methylbenzene, 2-bromonaphtalene, 1-bromo-4-methoxybenzene, 1-iodo-4-methoxybenzene, anisole, 1-iodo-4-methylbenzene, 1-iodonaphtalene, 1,1-diphenylethene, *N*,*N*-Diisopropylethylamine (DIPEA), benzophenone, *p*-nitroacetophenone. Ethanol 98 % (EtOH), acetonitrile (MeCN), tetrahydrofurane (THF), dimethyl sulfoxide (DMSO), *N*,*N*-Dimethylacetamide (DMA), and anhydrous Na_2SO_4 were used as received. Hexane, acetone and ethyl acetate solvents were analytical grade and distilled before use and toluene was distilled and dried under molecular sieves (3 Å). Milli-Q-Millipore water was employed in all the experiments. Silica gel (0.063–0.200 mm) was used in column chromatography.

The SNPs were characterized by Transmission Electron Microscopy (TEM) using a JEM-Jeol 1120 microscope operating at 80 kV, available at the Research Institute IPAVE- INTA-CIAP in Córdoba, Argentina. SEM microscopy was performed in a SEM - Zeiss Sigma 360 (Lamarx-UNC). The UV-vis measurements were carried out in a Shimadzu UV-2101 PC. Solid state fluorescence, and fluorescence in solutions were performed in Horiba Nanolog. FT-IR spectra were collected on an infrared microscope (Nicolet iN10, Thermo Scientifics, USA). Samples contained in KBr discs were scanned from 4000 to 400 cm^{-1} and the recording conditions were: normal resolution, sample scan, 64 s^{-1}. The spectra were recorded, processed and analyzed using the EZ OMNIC ESP 8.3.103 program. DLS measurements were performed by using a

Delsa Nano C instrument (Beckman Coulter, Osaka, Jp.). The hydrodynamic apparent diameter (d_H) was determined by Photon Correlation Spectroscopy at a controlled temperature of 25 °C in triplicate, using glass cuvettes. Intensity autocorrelation functions were analyzed with the Delsa Nano 2.20 software (Beckman Coulter, Osaka, Jp). Number-weighted particle size distribution was calculated assuming spherical morphology with constant refractive index.

A commercial Thermo Scientific K-Alpha X-ray photoelectron spectrometer (XPS) system (LAMARX, FaMAF-UNC), equipped with a hemispherical energy analyzer and a monochromated X-ray source was used for surveying the photoemission spectra. The base pressure measured in the main chamber was in the low 10-9 mbar range. The photoionization of the samples was induced by monochromatized Al Kα photons at 1486 eV. All the spectra were adjusted to the main spurious C 1s peak at 284.8 eV. To avoid any charging effects during measurement (typically observed in semiconductor-isolated systems), a flood gun to compensate the charge was used. The overcompensation effects by adjusting the spectra during measurement were also tested.

NMR spectra were conducted on a High-Resolution Spectrometer Bruker Advance 400, in $CDCl_3$ as solvent. Solid-state nuclear magnetic resonance (*ss*-NMR) experimental data were acquired with a Bruker Avance-III HD spectrometer equipped with a 14.1 T narrow bore magnet operating at Larmor frequencies of 600.09, 150.91 and 119.21 MHz for ^{1}H, ^{13}C and ^{29}Si, respectively. Powdered samples were packed into ZrO_2 rotors and rotated at different magic angle spinning (MAS) rates at room temperature. ^{29}Si *ss*-NMR spectra were recorded using high powered decoupling experiments at a MAS rate of 13 kHz in a 4-mm MAS probe with a recycling time of 30 seconds. ^{1}H-MAS *ss*-NMR spectra were recorded in a 2.5-mm MAS probe using single pulse excitation experiments at a MAS of 30 kHz. Chemical shifts for ^{1}H and ^{29}Si (in ppm) are relative to TMS. The ^{13}C cross-polarization and magic angle spinning (^{13}C CP-MAS) *ss*-NMR experiments were done in a 3.2-mm MAS probe at a MAS rate of 13 kHz. Glycine was used as external reference compound for the recording of the ^{13}C spectra and to set the Hartmann−Hahn matching condition in the CP-MAS experiments.[1] A contact time during CP of 2 ms and a recycling time of 5 seconds were used. The SPINAL64 sequence was used for heteronuclear decoupling during acquisition.[2] The number of scans for ^{13}C and ^{29}Si experiments was 10000 and 4000 for all the samples, respectively.

Gas chromatographic analyses were performed on a gas chromatograph with a flame ionization detector and equipped with the following columns: VF-5 30 m × 0.20 mm × 0.25 μm column.

Gas Chromatographic/Mass Spectrometer analyses were carried out on a GC/MS QP 5050 spectrometer equipped with a VF-5ms, 30 m × 0.25 mm × 0.25 μm column. UV-vis determinations were performed using a SHIMATZU UV-Vis UV- 1800 series. NMR were conducted on a High-Resolution Spectrometer Bruker Advance 400, in $CDCl_3$ as solvent.

2. General experimental methods

2.1. Synthesis of SiO_2 nanoparticles functionalized with amino group (SNPs-NH_2)

In a 100 mL round bottom flask equipped with a magnetic stir bar, were placed 60 mL of ethanol and 4.8 mL of NH_4OH aqueous solution (28 % w/w). After vigorously stirring of the mixture for 5 min, 2.5 mL of TEOS were added. This mixture was stirred for 24 h at room temperature.[3] Then, 200 μL of APTES were added, and the mixture was stirred for another 24 h. For purification of SNPs-NH_2, the reaction mixture was centrifuged for 8 min at 3500 rpm, the supernatant was discarded, and the white solid was washed with ethanol, sonicated by 5 min and centrifuged again. This procedure was repeated 3 times. Finally, the SNPs-NH_2 were dried under the air and at room temperature for 24 h. After this procedure, 670 mg of SNPs-NH_2 were recovered.

2.2. Immobilization of Succinic Acid onto SNPs-NH_2[4]

In a 10 mL Schlenk tube equipped with a magnetic stir bar and under a nitrogen atmosphere, 100 mg of succinic acid, 130 mg of HOBt (1 eq.), 175 mg of DCC (1 eq.), and 4 mL of acetone were added. The reaction mixture was heated under reflux for 1 hour. After cooling to room temperature, 200 mg of SNPs-NH_2 were added under a nitrogen atmosphere, and the mixture was stirred for 24 h. The resulting SNPs-COOH were centrifuged at 3500 rpm for 8 minutes, then suspended in ethanol, sonicated for 5 minutes, and centrifuged again for 8 minutes. The SNPs-COOH were subsequently washed with ethanol three times, following the same procedure each time. Finally, the SNPs-COOH were dried in air at room temperature for 24 h.

2.3. Immobilization of 1,5-Diaminoanthraquinone (DAAQ) onto SNPs-COOH[4]

In a 10 mL Schlenk tube equipped with a magnetic stir bar and under a nitrogen atmosphere, 200 mg of SNPs-COOH, 65 mg of HOBt (5 eq.), 87 mg of DCC (5 eq.), and 4 mL of acetone were added. The reaction mixture was heated under reflux for 1 hour. After cooling to room temperature, 20 mg of DAAQ (1 eq.) were added under a nitrogen atmosphere, and the mixture

was stirred for 24 h. The resulting SNPs-DAAQ were centrifuged at 3500 rpm for 8 minutes, then suspended in THF, sonicated for 5 minutes, and centrifuged again for 8 minutes. The SNPs-DAAQ were washed with acetone, acetonitrile (MeCN), and ethanol three times, following the same procedure each time. Finally, the SNPs-DAAQ were dried in air at room temperature for 24 h. After this procedure, 180 mg of SNPs-DAAQ were recovered.

2.4. Synthesis of *N*-Aryl-1,2,3,4-tetrahydroisoquinolines (3a-g)[5]

1,2,3,4-Tetrahydroisoquinoline (1.2 mmol, 1.2 equiv) and the corresponding aryl iodide (1.0 mmol, 1.0 equiv) were added to a suspension of $Pd(OAc)_2$ (0.05 mmol, 0.05 equiv), (±)-BINAP (0.055 mmol, 0.055 equiv), and *t*-BuOK (1.4 mmol, 1.4 equiv) in dry toluene (2 mL, 0.5 M). The reaction mixture was heated in an oil bath at 100 °C for 24 h under a nitrogen atmosphere. The reaction was quenched by adding water (10 mL) and extracted with EtOAc (3x10 mL). The combined organic phases were dried over anhydrous Na_2SO_4 and concentrated under vacuum. The crude product was purified by column chromatography (Hexane:EtOAc).

2.5. Photocatalyzed Aza-Henry reaction with SNPs-DAAQ catalysts

N-aryl-1,2,3,4-tetrahydroisoquinoline (0.1 mmol), SNPs-DAAQ (4 mol% with respect to DAAQ) and 1 mL of nitromethane were charged in a reaction glass tube with magnetic stirring bar. The reaction mixture was stirred under blue LED (465 nm, 48W, Figure SI2) irradiation at room temperature for 1.5 h under air atmosphere. After 1.5 h, the solvent was removed under vacuum, and the product was quantified by ^1H-NMR, with *p*-nitroacetophenone as internal standard. After quantification, the crude product was purified by column chromatography (Hexane:EtOAc).

2.6. Recycling Test

N-phenyl-1,2,3,4-tetrahydroisoquinoline (**3a**, 0.1 mmol), SNPs-DAAQ (4 mol% with respect to DAAQ) and 1 mL of nitromethane were placed in a reaction glass tube equipped with a magnetic stirring bar. The reaction mixture was stirred under blue LED irradiation (465 nm, 48W) at room temperature for 1.5 h under air atmosphere. After the reaction time, the crude was centrifugated and the solid catalyst was separated and dry under vacuo (this solid was stored for reuse in the next reaction cycle). The supernatant was evaporated, and the product was quantified by ^1H-NMR, using *p*-nitroacetophenone as internal standard.

2.7. Procedure for the photocatalytic dehalogenation of aryl halides[6]

In a 10 mL scintillation vial equipped with a magnetic stir bar, 0.1 mmol of the substrate (**1a**-**g**), SNPs-DAAQ (10 mol% DAAQ), and 5 mL of DMSO were added. The vial was sealed with a Teflon plug, and nitrogen was passed through for 5 minutes to saturate the solution. Next, using a syringe, *N*,*N*-Diisopropylethylamine (DIPEA, 8 equiv, as a sacrificial electron and hydrogen atom donor) was added. The vial was then irradiated with a 3 W blue LED under vigorous magnetic stirring for 24 h. After this time, 1 mL of a standard solution (2 mg/mL) was added to the reaction mixture. The mixture was then centrifuged at 3500 rpm for 8 minutes, and the supernatant was analyzed by GC using acetophenone or benzophenone as the internal standard.

2.8. Photocatalytic dehalogenation of 1a in the presence of 1,1-diphenylethene: Procedure for radical trapping. In a 10 mL scintillation vial equipped with a magnetic stir bar, 0.1 mmol of 4-bromobenzonitrile (**1a**), SNPs-DAAQ (10 mol% DAAQ), 0.1 mmol of 1,1-diphenylethene, and 5 mL of DMSO were added. The vial was sealed with a Teflon plug, and nitrogen was passed through for 5 minutes to saturate the solution. Next, using a syringe, *N*,*N*-Diisopropylethylamine (DIPEA, 8 equiv, as a sacrificial electron and hydrogen atom donor) was added. The vial was then irradiated with a 3 W blue LED under vigorous magnetic stirring for 24 h. After this time, 1 mL of a standard solution (2 mg/mL) was added to the reaction mixture. The mixture was then centrifuged at 3500 rpm for 8 minutes, and the supernatant was analyzed by GC-MS. **4-(2,2-diphenylvinyl)benzonitrile** GC-MS (EI): *m/z* 282 (M^+ +1, 23), 281 (M^+, 83), 280 (25), 278 (13), 266 (12), 253 (15), 208 (10), 207 (28), 204 (13), 203 (23), 167 (31), 165 (21), 152 (13), 151 (11), 133 (13), 126 (14), 125 (13), 96 (15), 91 (34), 89 (12), 87 (10), 79 (11), 78 (100), 77 (32), 76 (11), 75 (15), 74 (12), 73 (30), 65 (11), 63 (81), 62 (18), 61 (23), 51 (27).

2.9. Photooxidation of DMA

5 mL of a 9,10-dimethylanthracene (DMA, 6×10^{-5} M) solution in EtOH and 10 mol% of the catalyst were added to a vial containing a stir bar. The reaction mixture was then irradiated with a blue LED (3W) lamp while stirring at room temperature until the DMA was fully consumed. UV-Visible spectra were recorded at various irradiation times. The decrease in absorbance at λ = 379 nm, corresponding to DMA, was used to monitor the oxidation process.

3. Photochemical reactors

Photoinduced reactions were conducted with blue LED (λ= (465 ± 20) nm) lights performing at 3W of potency and 700 mV of current emission spectra (Figure S1) and a LED photochemical reactor, equipped with 16 (3W) blue LEDs lamps (48W blue LED). Apparatus and irradiation setup is shown in Figure SI2.

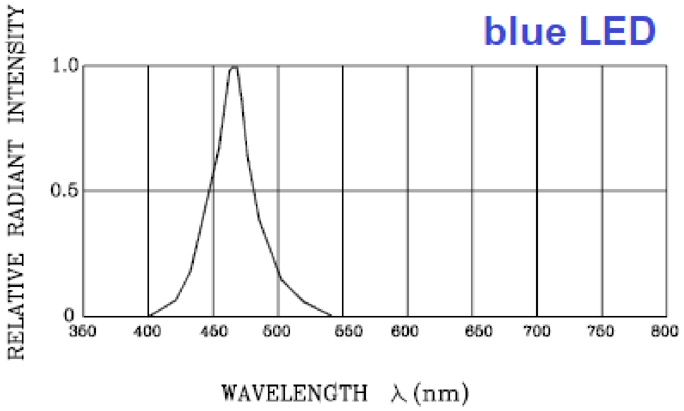

Figure SI1. Emission Spectra for blue LEDs as provided by the supplier.

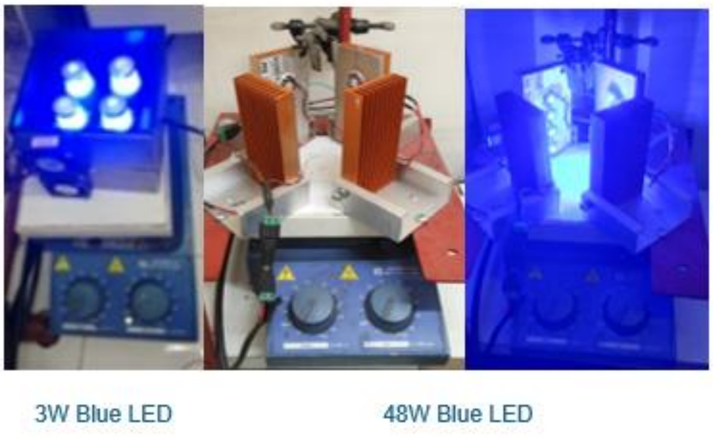

Figure SI2. Apparatus and irradiation setup used.

Table SI1. Details of light sources employed

	Blue LEDs
**Manufacture and model**	OSRAM Oslon
**Potency**	3 W
**Intensity**	0.127 (W/cm^2) at 1 cm of distance
**Wavelength of peak intensity**	(465 ± 20) nm
**Spectral distribution and intensity**	See Figure S1
**Distance from the light source**	1 cm
**Material of the irradiation vessel**	borosilicate glass

4. Characterization of SNPs materials

4.1. Transmission Electron Microscopy (TEM) and Scanning electron microscopy (SEM)

The SNPs were characterized by Transmission Electron Microscopy (TEM) using a JEM-Jeol 1120 microscope operating at 80 kV, available at the Research Institute IPAVE- INTA-CIAP in Córdoba, Argentina. Average dimensions and shape of all SNPs were determined by Transmission Electron Microscope (TEM) images (Figure SI3). Samples for morphological characterization were prepared by depositing a drop of the colloidal suspension on a 300 mesh formvar-carbon coated copper grid and dried at room temperature. Size distribution of SNPs was established by the average over 100 NPs from different places at each image of all samples. Distribution plots of the size were resolved by fitting data with a Gaussian behavior.
Scanning electron microscopy (SEM) was performed in a SEM - Zeiss Sigma 360 (Lamarx-UNC). For SEM microscopy the sample were deposited in a carbon substrate and covered with Au.

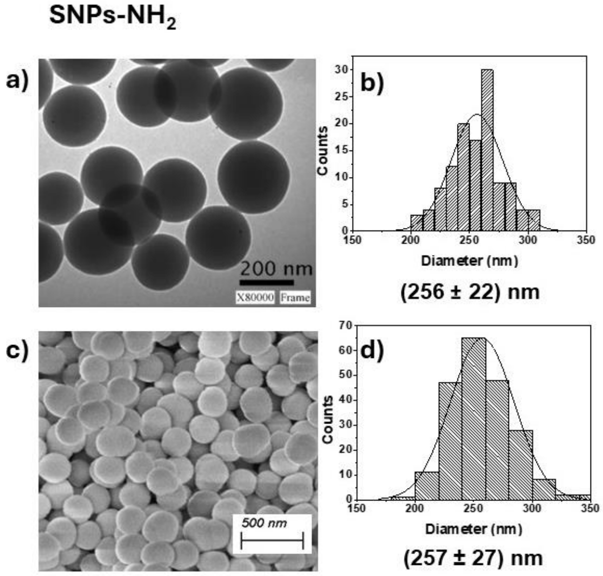

Figure SI3. a) TEM micrograph and b) size distribution analysis for SNPs-NH_2 c) SEM micrograph and d) size distribution analysis for SNPs-NH_2.

4.2. UV-vis

The UV-vis measurements were performed using a Shimadzu UV-2101 PC spectrophotometer. All measurements were conducted under air atmosphere, in quartz cuvettes, at room

temperature. Suspensions of SNPs-DAAQ in DMSO were prepared at time of use. UV-vis spectra were recorded (Figure SI4).

A suspension of SNPs-DAAQ (0.3 mg/mL) was prepared, and the UV-Vis spectrum was recorded. Due to the low solubility of DAAQ in ethanol, quantitative UV-vis analyses were performed in DMSO as solvent. In those cases, absorption spectra present two maximum shifts at 400 nm and 415 nm (Figure SI4). The absorbance at the maximum wavelength at 400 nm was compared to a calibration curve of homogeneous DAAQ in DMSO. Based on the absorbance obtained, a loading of 0.157 mmol of DAAQ per gram of nanomaterial was calculated.

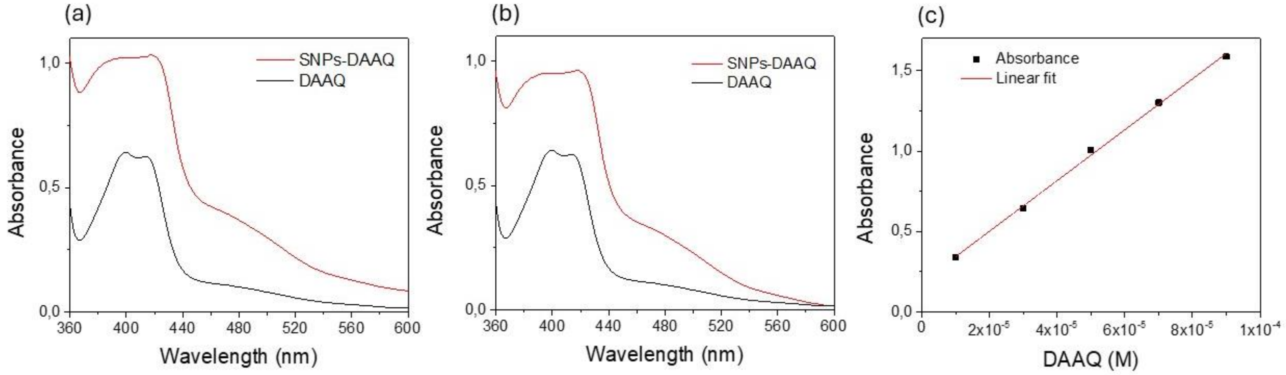

Figure SI4. UV-vis spectrum DAAQ in DMSO solution (black, conc. 3 x 10^{-5} M) and DMSO suspension of SNPs-DAAQ (red, 0.3 mg/mL) before baseline correction (a) and after baseline correction (b). Calibration curve of DAAQ in DMSO solution (c).

4.3. FT-IR analyses

Samples contained in KBr discs were scanned from 4000 to 400 cm^{-1} and the recording conditions were: normal resolution, sample scan, 64 s^{-1}. The spectra were recorded, processed and analyzed using the EZ OMNIC ESP 8.3.103 program (Figure SI5).

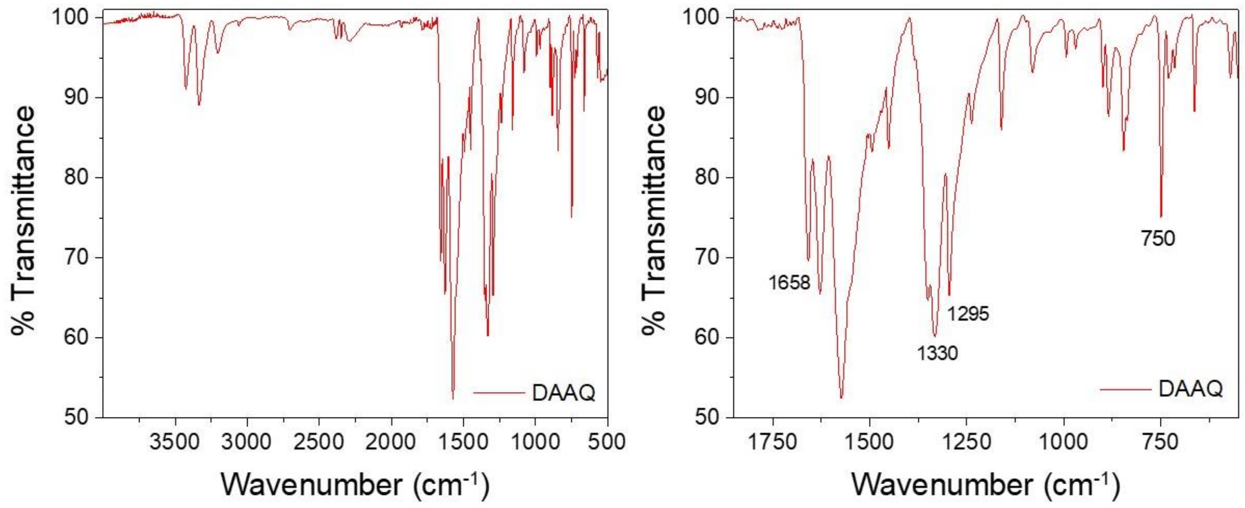

Figure SI5. FT-IR analysis for DAAQ.

4.4. *ss*-NMR analysis

Solid-state nuclear magnetic resonance (*ss*-NMR) experimental data were acquired with a Bruker Avance-III HD spectrometer equipped with a 14.1 T narrow bore magnet operating at Larmor frequencies of 600.09, 150.91 and 119.21 MHz for ^{1}H, ^{13}C and ^{29}Si, respectively. Figure SI6 shows ^{1}H-MAS *ss*-NMR spectra of SNPs, SNPs-NH_2, SNPs-COOH and SNPs-DAAQ. In the different SNPs, the main ^{1}H NMR signal at 4.6 ppm was assigned to bulk physiosorbed water molecules that involve weakly hydrogen bond interactions. The broad ^{1}H resonance signal at 6–7 ppm and the narrow signal at 0.9 ppm may be associated with water molecules that are strongly hydrogen-bonded to the silanol protons of silica core and isolated water molecules, respectively.[7,8] Additionally, isolated silanol groups can be shown at 0.9 ppm.[9] Besides, the ^{1}H signals at 1.1 and 3.3-3.6 ppm were assigned to the residual ethoxy groups present in the silica structure. Among the different treatments performed on the SNPs, it was not possible to infer the different chemical modifications by ^{1}H *ss*-NMR. Particularly, the ^{1}H *ss*-NMR spectra for the SNPs-COOH and SNPs-DAAQ showed a new signal at 2.1 ppm associated to the methylene segments of the incorporated succinic acid.

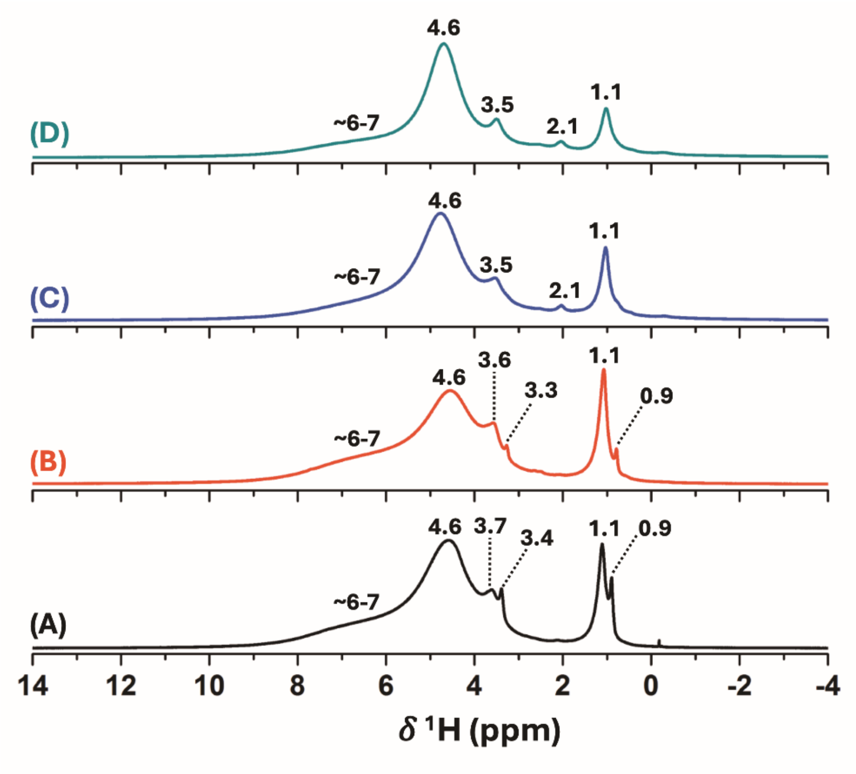

Figure SI6. ^{1}H MAS spectra (MAS rate: 30 kHz) for the SNPs (A), SNPs-NH_2 (B), SNPs-COOH (C) and SNPs-DAAQ (D) samples.

From the ^{29}Si *ss*-NMR spectra only the Q^4 (***Si***$(OSi)_4$), Q^3 (***Si***$(OSi)_3OH$) and Q^2 (***Si***$(OSi)_2(OH)_2$) sites were present in the different SNPs at a δ ^{29}Si of -110 (50%), -101 (45%) and -92 ppm (5%), respectively (Figure SI7). The area of the Q^n sites was similar among the different SNPs. It is important to remark that the chemical modification was not possible to infer from the ^{29}Si direct polarization *ss*-NMR experiments (the T^n bands were not observed)[10] due to the low degree of modification related to the silica core.

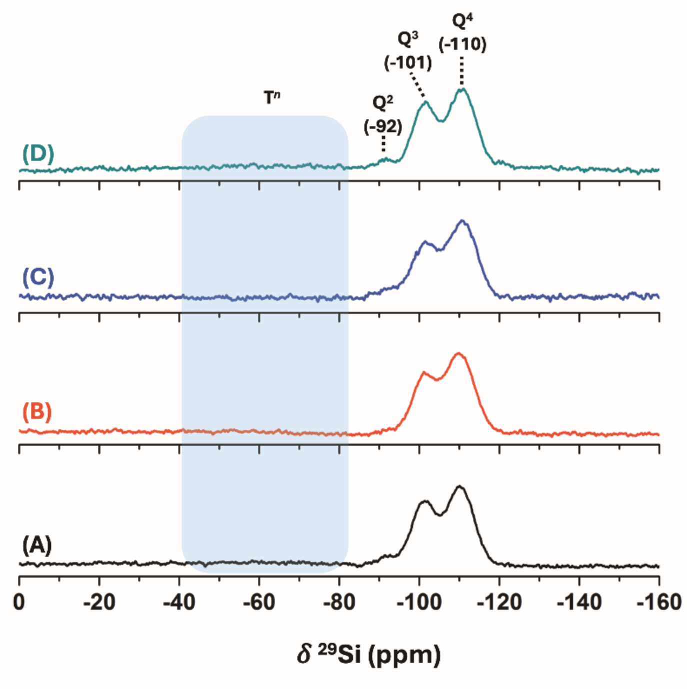

Figure SI7. ^{29}Si DP-MAS spectra (MAS rate: 13 kHz) for the SNPs (A), SNPs-NH_2 (B), SNPs-COOH (C) and SNPs-DAAQ (D) samples.

Table SI2. Organic elemental analysis of the different SNPs.

Material	% C	% H	% N
**SNPs**	0.87	1.49	0
**SNPs-NH_2**	1.85	1.63	0.08
**SNPs-DAAQ**	11.15	1.95	1.76

4.5. XPS measurements

A commercial Thermo Scientific K-Alpha X-ray photoelectron spectrometer (XPS) system (LAMARX, FaMAF-UNC), equipped with a hemispherical energy analyzer and a monochromated X-ray source was used for surveying the photoemission spectra. The base pressure measured in the main chamber was in the low 10^{-9} mbar range. The photoionization of the samples was induced by monochromatized Al Kα photons at 1486 eV. All the spectra were adjusted to the main spurious C *1s* peak at 284.8 eV. To avoid any charging effects during measurement (typically observed in semiconductor-isolated systems), a flood gun to compensate the charge was used. The overcompensation effects by adjusting the spectra during measurement were also tested.

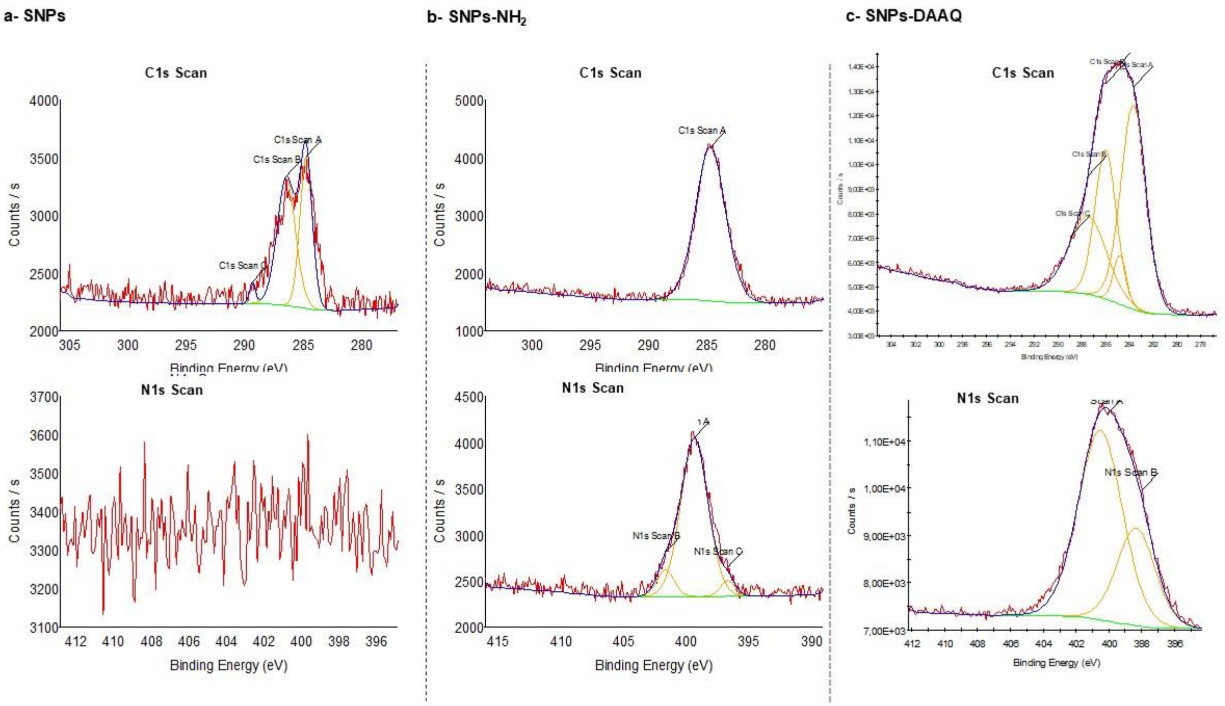

Figure SI8. High-resolution XP spectra for (a) C 1s and N 1s of SNPs (b) C 1s and N 1s of SNPs-NH_2 (c) C 1s, and N 1s of SNPs-DAAQ.

Table SI3. Percentage atomic concentration on SiO_2, SNPs-NH_2 and SNPs-DAAQ surface from XPS results

Sample	% atomic			
	Si	O	C	N
SiO_2	31	66	3	
SNPs-NH_2	29	54	13	4
SNPs-DAAQ	23	44	28	5

4.6. DLS measurements

DLS measurements were performed by using a Delsa Nano C instrument (Beckman Coulter, Osaka, Jp.). The hydrodynamic apparent diameter (d_H) was determined by photon correlation spectroscopy at a controlled temperature of 25 °C in triplicate, using glass cuvettes. Intensity autocorrelation functions were analyzed with the Delsa Nano 2.20 software (Beckman Coulter, Osaka, Jp). Number-weighted particle size distribution was calculated assuming spherical morphology with constant refractive index. Colloidal suspensions of SNPs-DAAQ in different solvents (ethanol (EtOH), water, nitromethane and MeCN) were analyzed. All NPs suspension

of concentration ~1 mg/mL were sonicated for 30 min before performing the DLS measurements.

Colloidal suspensions of SNPs-DAAQ in different solvents were analyzed by Dynamic Light Scattering (DLS) technique (Figure SI9), to control the stability and dispersion of this nanomaterial in different media. All the samples analyzed showed two distribution curves: a predominant one with hydrodynamic diameters (d_H) centered around 250 nm and small populations with hydrodynamic diameters (d_H) above 8000 nm, except for the SNPs-DAAQ suspended in water, where a third distribution curve centered at 782 nm was observed, suggesting greater nanoparticle agglomeration in this solvent. This phenomenon is also reflected in an increase in the PDI value compared to the other solvents analyzed. The predominant distribution curves show similar hydrodynamic diameters of (210 ± 57) nm, (302 ± 32) nm, (292 ± 54) nm, and (252 ± 28) nm for EtOH, nitromethane, water, and MeCN, respectively (Table SI4), indicating a good correlation with TEM images (277 ± 27 nm).

Table SI4: SNPs-DAAQ hydrodynamics diameter in different solvent.

**Solvent**	**d_H (nm)**	**d_H (nm)**	**d_H (nm)**	**PDI**
EtOH	210 ± 57	-	8623 ± 1780	0.397
Nitromethane	302 ± 32	-	20737 ± 3157	0.460
Water	292 ± 54	782 ± 122	35060 ± 4406	0.602
MeCN	252.4 ± 27.9	-	21081 ± 3250	0.368

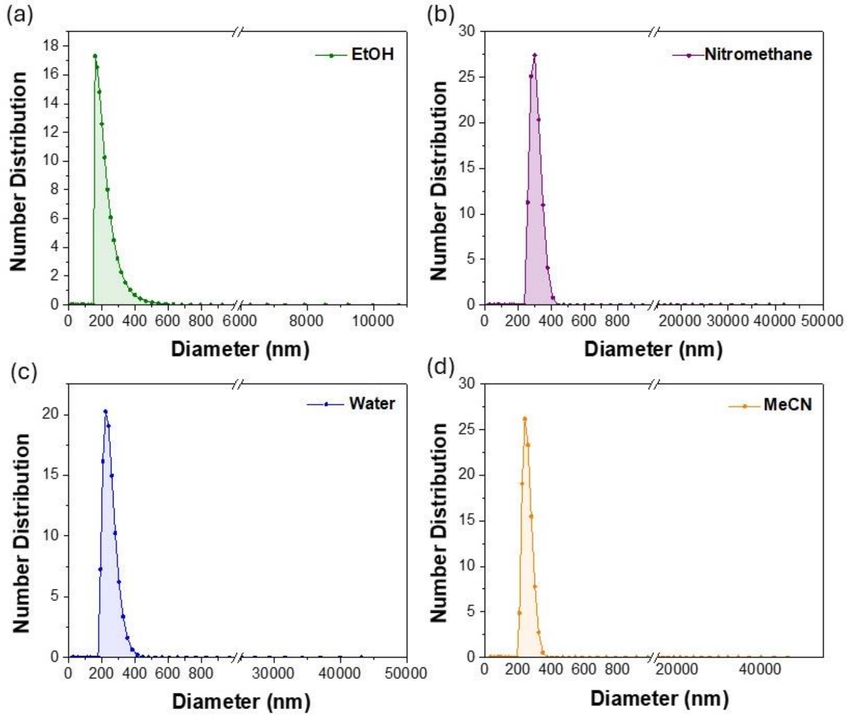

Figure SI9. Hydrodynamic diameter d_H (nm) of SNPs-DAAQ and number distribution suspended in EtOH (a), Nitromethane (b) Water (c) and MeCN (d).

4.7. Characterization of the Recycled Nanomaterial

The recycled SNPs-DAAQ were characterized by TEM and FT-IR (Figure SI10). TEM analysis of the recycled SNPs-DAAQ was performed using a JEM-Jeol 1120 microscope operating at 80 kV, available at the Research Institute IPAVE-INTA-CIAP in Córdoba, Argentina. The FT-IR spectrum of the recycled SNPs-DAAQ was obtained using an infrared microscope (Nicolet iN10, Thermo Scientific, USA). Samples in KBr discs were scanned from 4000 to 400 cm^{-1} under the following conditions: normal resolution, 64 scans per second. The spectra were recorded, processed, and analyzed using the EZ OMNIC ESP 8.3.103 software.

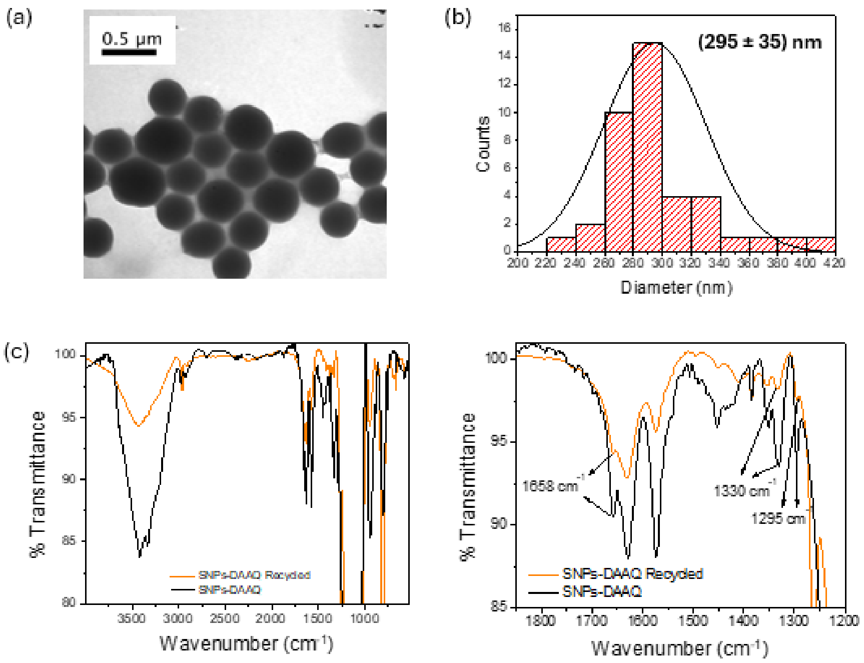

Figure SI10. a) TEM micrograph and b) size distribution analysis for recycled SNPs-DAAQ c) Comparative FT-IR analysis for SNPs-DAAQ (black) and recycled SNPs-DAAQ (orange).

5. Dehalogenation of aryl halides

5.1. Plausible reaction mechanism for dehalogenation reaction of aryl halides

Initially, the photocatalyst is excited by the absorption of visible light generating SNPs-DAAQ* (Scheme SI1). In the next step, an electron transfer occurs between the donor DIPEA and the excited state of the photocatalyst, generating the radical cation DIPEA$^{\bullet +}$ and the radical anion of photocatalysts (SNPs-DAAQ$^{\bullet -}$). The SNPs-DAAQ$^{\bullet -}$ radical anion is further excited by visible light, forming the excited radical anion, which transfers an extra electron to **1a**, returning the photocatalyst to its ground state while simultaneously generating a radical aryl (**Ar**$^{\bullet}$, after C-Br bond fragmentation), which, finally abstracts a hydrogen atom, leading to the formation of product **2a.**

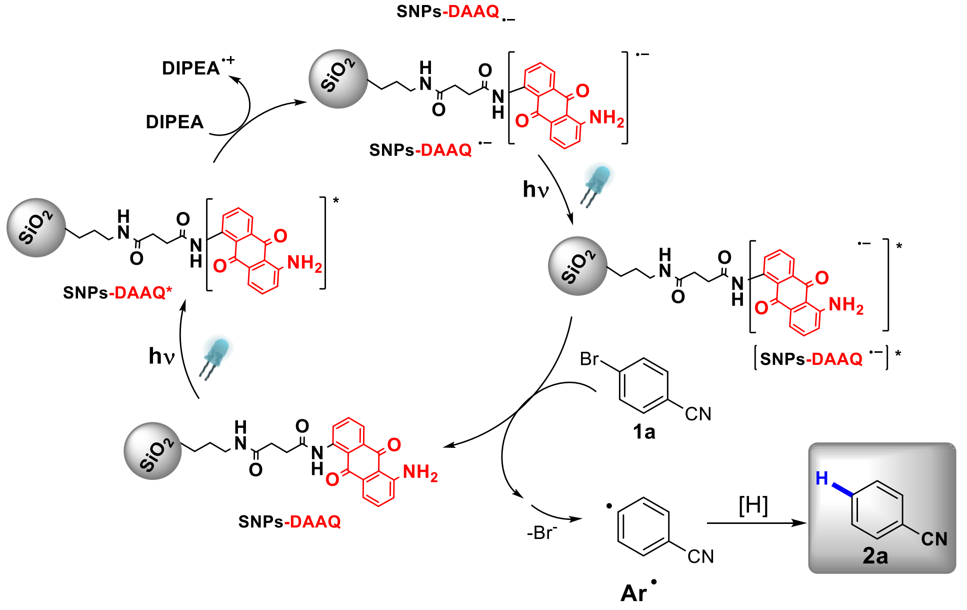

Scheme SI1. Proposed Mechanism for the dehalogenation of aryl halides in presence of SNPs-DAAQ.

5.2. Comparison of heterogeneous photocatalyst in dehalogenation reaction of aryl halides

Table SI5. Comparison of reported heterogeneous photocatalysts in the dehalogenation reaction of aryl halides with heterogeneous SNPs-DAAQ.

Entry	Substrate	PC	Catalyst loading	Lamp	Donor	Solvent	Time (h)	Conversion of aryl halide (%)	Ref
1	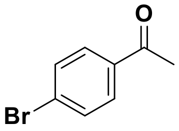 **1b**	SNPs-DAAQ	10 %	Blue LED 3 W	DIPEA	DMSO	24	100	This work
2[a]	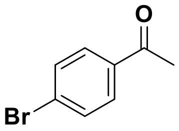 **1b**	Zr-MOF-OH	4 mg	White LED 5W	DIPEA/H_2O	MeCN/MeOH	10	98	[11]
3	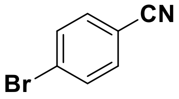 **1a**	SNPs-DAAQ	10 %	Blue LED 3 W	DIPEA	DMSO	24	77	This work

4	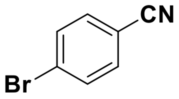 **1a**	QD-CdZn	0.6 mol%	Blue LED (3.5 V, 700 mA)	DIPEA	Hexane	24	72	[12]
5	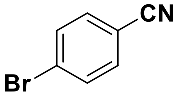 **1a**	Phen-CTF	1 mg	Purple LED 15 W	NBu_3/ HCOOH	ACN	17	78	[13]

[a] Extra additives: Cs_2CO_3, triethylene glycol dimethyl ether.

6. Detection of intermediates in CDC reaction

6.1. H_2O_2 determination

The detection of the H_2O_2 in the reaction mixture of photoredox catalytic CDC reaction was performed by the following procedure: Substrate **3a** (0.1 mmol), nitromethane (1 mL) and SNPs-DAAQ (25 mg) were charged in a 10 mL scintillation vial equipped with a magnetic stir vial. Next, the vial was placed into the photochemical reactor and irradiated under vigorous magnetic stirring for 1.5 h. Finally, the reaction mixture was centrifugated and crude was dropped to a solution containing 1 mL of KI (0.1 M) and 1 mL of aqueous acetic acid (0.1M). This solution is show is Figure SI9b. Then, we added starch and observed the characteristic change of color to a dark blue mixture, indicated the formation of I_2/starch complex generated by H_2O_2 formation (Figure SI11c).

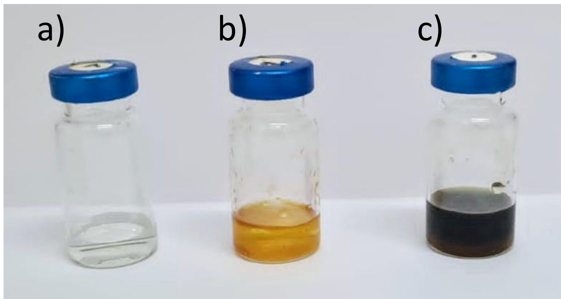

Figure SI11. a) Picture of solution of 1 mL of KI (0.1 M) and 1 mL of aqueous acetic acid (0.1M). b) The picture of KI, aqueous acetic acid, and 1 mL of crude of aza-Henry reaction (after 1.5 h of irradiation). c) The picture of KI, aqueous acetic acid, starch, and 1 mL of crude of aza-Henry reaction (after 1.5 h of irradiation).

6.2. Iminium intermediate identification

The iminium cation **5** was detected performing a reaction in isopropanol as solvent, irradiating a mixture of **3a**, SNPs-DAAQ under air for 1.5 h. Then, the mixture was centrifugated, and the supernatant was analysed by ^{1}H NMR analyses (Figure SI12).

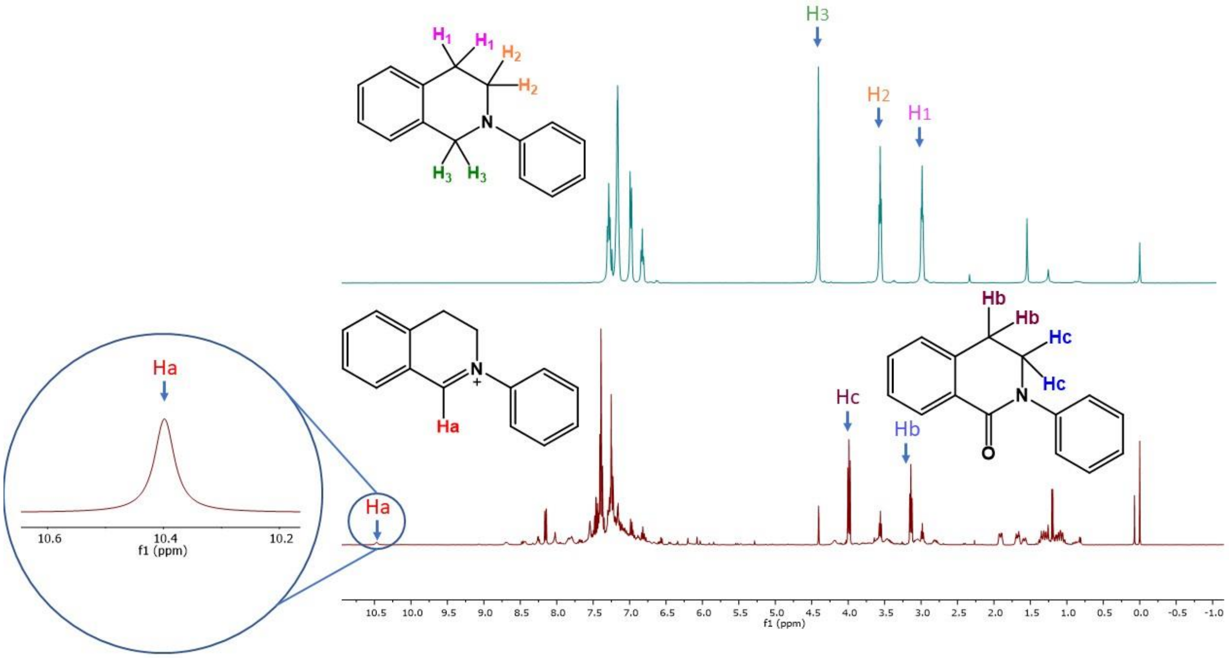

Figure SI12. ^{1}H NMR analyses of a) reaction mixture in absence of nitromethane and b) substrate **3a**.

The signals of iminium **5** and isoquinolone in the ^{1}H NMR spectra were identified from those already reported elsewhere.[5],[14]

6.3. Plausible reaction mechanism for CDC Reaction

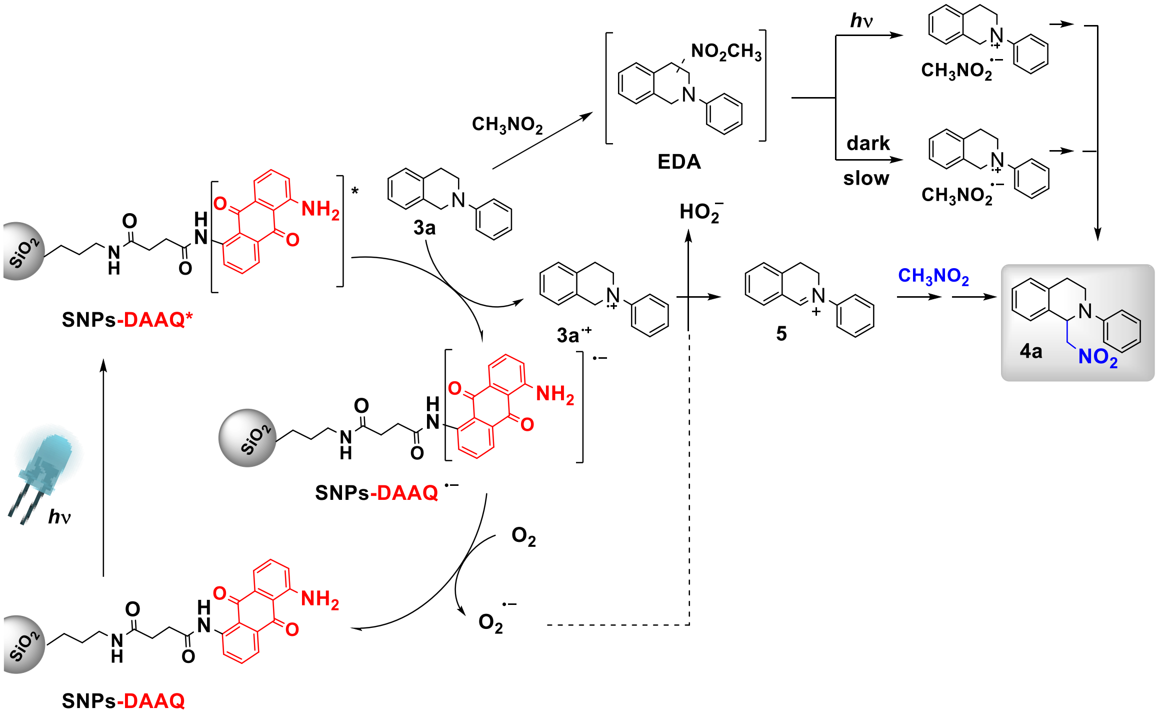

Scheme SI2. Proposed mechanism for CDC reaction of **3a** and nitromethane in presence of SNPs-DAAQ

Initially, the photocatalyst SNPs-DAAQ is excited to SNPs-DAAQ* upon absorbing visible light. In the next step, *N*-phenyltetrahydroisoquinoline **3a** undergoes reductive quenching, generating the radical cation intermediate **3a**$^{\bullet+}$ along with the formation of the radical anion (SNPs-DAAQ$^{\bullet-}$). This radical anion transfers an electron to oxygen, returning the photocatalyst to its ground state while simultaneously generating $O_2^{\bullet-}$. Meanwhile, the radical cation intermediate **3a**$^{\bullet+}$ is converted into the iminium intermediate **5** through hydrogen abstraction by $O_2^{\bullet-}$. Finally, the CDC product **4a** is formed via nucleophilic attack by nitromethane to iminium intermediate **5**. Moreover, the literature indicates that **3a** and nitromethane form an electron donor-acceptor (**EDA**) complex. In the absence of light, a slow electron transfer reaction takes place, producing the radical cation **3a**$^{\bullet+}$ and the radical anion of nitromethane ($CH_3NO_2^{\bullet-}$), which ultimately yields product **4a** (4% yield after 1.5 h, entry 6, Table 2). Conversely, in the absence of the photocatalyst, light excitation facilitates charge transfer in the **EDA** complex between nitromethane and **3a**, generating the corresponding

radical ion pair, which further evolves to produce **4a** with an 11% yield after 1 h (entry 5, Table 2).

7. Photocatalytic oxidation of dimethylanthracene (DMA)

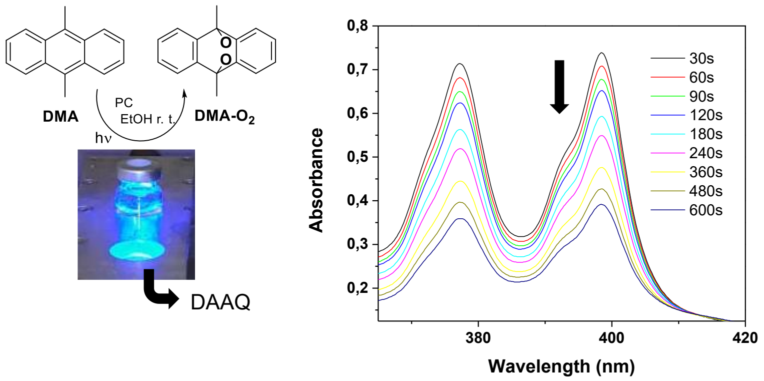

Figure SI13. Photocatalytic oxidation of **DMA** (6×10^{-5} M) to 9,10-endoperoxide. Decrease in absorbance of **DMA** *vs.* irradiation time using DAAQ (6×10^{-6} M). Irradiation conditions: Blue LED 3W lamp and EtOH as the solvent.

9,10-dimethylanthracene (DMA, 6×10^{-5} moles) was dissolved in EtOH (5 mL) and 10 mol% of the catalyst (SNPs-DAAQ) were added to a vial containing a stir bar. The reaction mixture was then irradiated with a blue LED (3W) lamp while stirring at room temperature for 1500 seconds. The solvent was removed, the 4-nitroacetophenone and $CDCl_3$ were added. ^{1}H NMR spectrum was recorded (Figure SI14). Moreover, the spectrum of the DMA-O_2 endoperoxide, was obtained by irradiating the DMA (6×10^{-5} moles) and SNPs-DAAQ (10 mol%) reaction in ethanol with a blue LED for 1500 seconds, followed by solvent evaporation and addition of $CDCl_3$

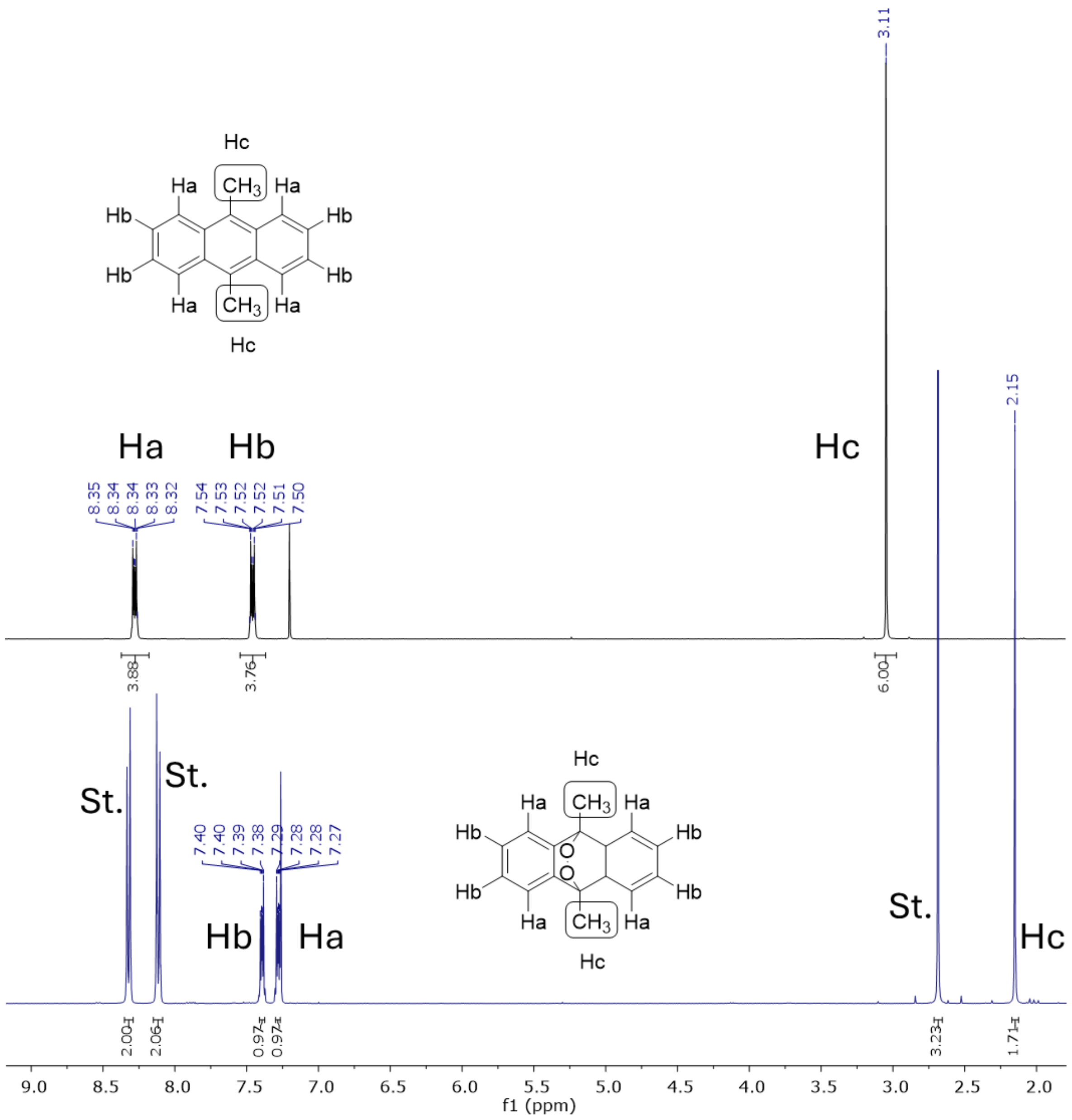

Figure SI14. a) ^{1}H NMR of **DMA** in $CDCl_3$. b) ^{1}H NMR of **DMA-O$_2$** and 4-nitroacetophenone (St.) in $CDCl_3$.

8. Photodynamic inactivation

8.1. Bacterial culture

Methicillin-sensitive *S. aureus* ATCC 29213 (MSSA) was used in this study. This strain was grown overnight at 37 ºC in TSA. After reaching the stationary phase of growth, the cultures were resuspended in PBS and measured at 600 nm by a spectrophotometer, yielding an optical density (OD) of about 0.08–0.10 (corresponding to 0.5 McFarland scale). Finally, a 1/ 100

dilution of McFarland (cellular density: 10^6 CFU/mL) was prepared and used for the photodynamic inactivation experiments.

8.2. *In vitro* phototoxicity assays

An aliquot of the bacterial suspension (1 mL) was placed in a test tube together with 1 mL of different concentrations of the PS solutions. The solutions of the dyes were prepared from a stock solution of DAAQ in DMSO and SNPs-DAAQ and then diluted with PBS to obtain 120–240 μM solutions.

S. aureus ATCC 29213 was pre-incubated for 15 min at different concentrations of each PS in a sterility for this assay. In order to perform the *in vitro* phototoxicity assays the test tubes were illuminated with a blue LED lamp (3 W) for 30 min. After that, the aliquots were removed, 10-fold serially diluted in PBS, cultured in Petri dishes containing TSA and incubated overnight at 37 ºC. Colony Forming Units (CFU) were counted and survival fractions were determined in triplicate by the drop-plate technique for bacterial enumeration according to Naghili *et. al.*. [15] The values were expressed as the mean.

For the control samples, the same set-up was used in the absence of the 3 W blue LED light to evaluate the toxicity of DAAQ and SNPs-DAAQ.

9. Photostability of homogeneous and heterogeneous photocatalysts

The photostability test was performed by irradiating a DAAQ solution in DMSO (DAAQ, 5×10^{-5} M) employing a blue LED (3W) lamp while stirring at room temperature for 5h and 24 h. The same experiment was for a DMSO dispersion of SNPs-DAAQ (0.4 mg/mL). After these times the UV-visible spectra were recorded.

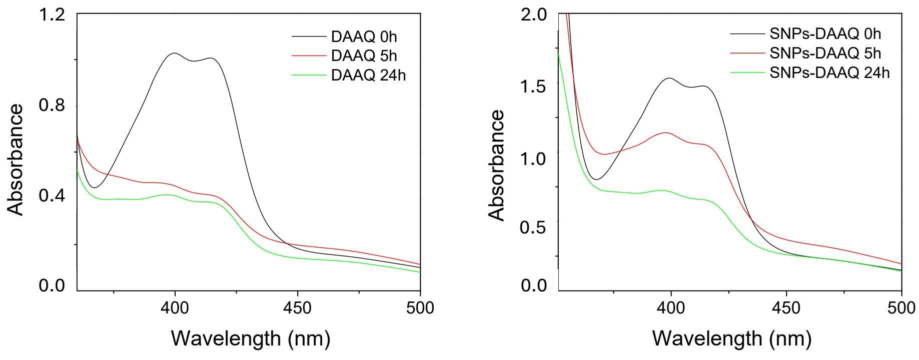

Figure SI15. Photostability test of DAAQ and SNPs-DAAQ in DMSO, irradiated for 24 h with a 3 W blue LED light.

Table SI 6: Photostability of DAAQ and SNPs-DAAQ over time

Time (h)	Concentration (M)	Photodegradation percentage %	Concentration (M)	Photodegradation percentage %
	DAAQ		SNPs-DAAQ	
0	5.30×10^{-5}	0	8.53×10^{-5}	0
5	1.70×10^{-5}	68	5.99×10^{-5}	30
24	1.40×10^{-5}	74	3.36×10^{-5}	61

10. Characterization Data of Compounds 2a-h, 3a-g, 4a-h and DMA-O_2

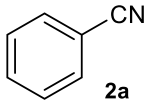

2a

Benzonitrile (**2a**). **^{1}H NMR** (400 MHz, $CDCl_3$) δ 7.66 (dd, *J* = 8.3, 1.4 Hz, 2H), 7.61 (ddt, *J* = 9.0, 7.2, 1.4 Hz, 1H), 7.51 – 7.43 (m, 2H). **^{13}C NMR {^{1}H}** (101 MHz, $CDCl_3$) δ 132.9, 132.3, 129.3, 119.0, 112.6. **GC-MS (EI):** *m*/*z* 103 (M^+, 100), 76 (52), 75 (11), 51 (17), 50 (28).

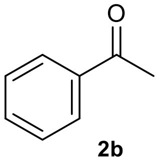

2b

Acetophenone (**2b**). **^{1}H NMR** (400 MHz, $CDCl_3$) δ 8.00 – 7.92 (m, 2H), 7.60 – 7.53 (m, 1H), 7.50 – 7.42 (m, 2H), 2.61 (s, 3H). **^{13}C NMR {^{1}H}** (101 MHz, $CDCl_3$) δ 198.3, 137.3, 133.2, 128.7, 128.4, 26.7. **GC-MS (EI):** *m*/*z* 120 (M^+, 27), 105 (91), 78 (10), 77 (100), 51 (48), 50 (16).

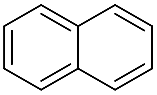

2c

Naphthalene (**2c**). **^{1}H NMR** (400 MHz, $CDCl_3$) δ 7.86 (dd, *J* = 6.3, 3.4 Hz, 4H), 7.50 (dd, *J* = 6.4, 3.3 Hz, 4H). **^{13}C NMR {^{1}H}** (101 MHz, $CDCl_3$) δ 133.6, 128.0, 126.0. **GC-MS (EI):** *m*/*z* 129 (M^+ +1, 10), 128 (M^+, 100), 127 (13), 102 (12), 64 (14), 63 (11), 51 (22).

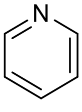

2d

Pyridine (**2d**). **^{1}H NMR** (400 MHz, $CDCl_3$) δ 8.84 – 8.34 (m, 2H), 7.67 (tt, *J* = 7.6, 1.8 Hz, 1H), 7.28 (ddd, *J* = 7.6, 4.3, 1.5 Hz, 2H). **^{13}C NMR {^{1}H}** (101 MHz, $CDCl_3$) δ 150.0, 136.0, 123.8. **GC-MS (EI):** *m*/*z* 79 (M^+, 100), 78 (11), 52 (76), 51 (32) 50 (23).

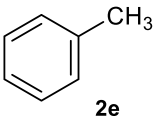

2e

Toluene (2e). ^{1}H NMR (400 MHz, $CDCl_3$) δ 7.34 – 7.22 (m, 2H), 7.21 – 7.12 (m, 3H), 2.35 (s, 3H). **^{13}C NMR {^{1}H}** (101 MHz, $CDCl_3$) δ 138.0, 129.2, 128.4, 125.4, 21.6. **GC-MS (EI):** *m*/*z* 92 (M^+, 77), 91 (100), 65 (12).

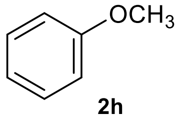

2h

Anisole (2h). **^{1}H NMR** (400 MHz, $CDCl_3$) δ 7.33 (dd, *J* = 8.7, 7.4 Hz, 2H), 7.06 – 6.88 (m, 3H), 3.84 (s, 3H). **^{13}C NMR {^{1}H}** (101 MHz, $CDCl_3$) δ 159.6, 129.6, 120.8, 114.0, 55.2. **GC-MS (EI):** *m*/*z* 108 (M^+, 55), 93 (12), 79 (24), 78 (100), 77 (25), 65 (58), 51 (22), 50 (11).

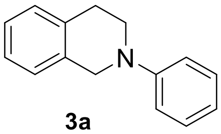

3a

2-phenyl-1,2,3,4-tetrahydroisoquinoline (3a).[16] Starting from 1 mmol of iodobenzene (section 2.4). The product was purified by column chromatography on silica gel eluting with hexane/EtOAc (90:10). Pale-yellow solid was obtained in 52% yield (110 mg, 0.52 mmol). **Melting point**: 40.8-42.4°C. **IR (KBr)**: ν_{max} 3056, 3022, 2909, 2824, 1599, 1575, 1504, 1448,1388, 1337, 1292, 1227, 1156, 1111, 1031, 990, 872, 803, 149, 691, 514. **^{1}H NMR** (400 MHz, $CDCl_3$) δ 7.36 – 7.11 (m, 6H), 6.98 (d, *J* = 8.1 Hz, 2H), 6.82 (t, *J* = 7.3 Hz, 1H), 4.41 (s, 2H), 3.56 (t, *J* = 5.8 Hz, 2H), 2.99 (t, *J* = 5.8 Hz, 2H). **^{13}C NMR {^{1}H}** (101 MHz, $CDCl_3$) δ 150.7, 135.0, 134.6, 129.3, 128.6, 126.7, 126.5, 126.2, 118.8, 115.3, 50.9, 46.7, 29.3. **GC-MS (EI):** m/z 210 (M^+ +1, 15), 209 (M^+, 100), 208 (92), 105 (16), 104 (88), 103 (26), 91 (11), 78 (33), 77 (52), 51(22), 50 (5).

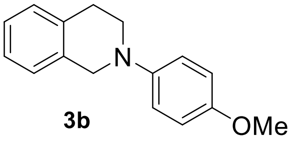

3b

2-(4-methoxyphenyl)-1,2,3,4-tetrahydroisoquinoline (3b).[16] Starting from 1 mmol of 1-iodo-4-methoxybenzene (section 2.4). The product was purified by column chromatography on silica gel eluting with hexane/EtOAc (90:10). White solid was obtained in 30% yield (71.8 mg, 0.30 mmol). **Melting point:** 87.7-89.3°C. **IR (KBr):** ν_{max} 3038, 2996, 2958, 2897, 2807, 1583, 1510, 1459, 1442, 1384, 1349, 1331, 1300, 1273, 1243, 1207, 1189, 1151, 1112, 1037, 945, 930, 867, 824, 756, 721, 702, 543, 526. **^{1}H NMR** (400 MHz, $CDCl_3$) δ 7.20 – 7.11 (m, 4H), 7.01 – 6.95 (m, 2H), 6.90 – 6.84 (m, 2H), 4.30 (s, 2H), 3.78 (s, 3H), 3.45 (t, *J* = 5.9 Hz, 2H), 2.99 (t, *J*= 5.8 Hz, 2H). **^{13}C NMR {^{1}H}** (101 MHz, $CDCl_3$) δ 153.6, 145.5, 134.8, 134.7, 128.8, 126.6, 126.4,

126.1, 118.2, 114.8, 55.8, 52.8, 48.6, 29.3. **GC-MS (EI):** m/z 240 (M^+ +1, 15), 239 (M^+ ,100), 238 (80), 224 (19), 135 (20), 120 (26), 115 (10), 104 (32), 78 (21), 77 (17).

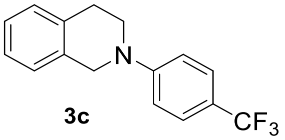

3c

2-(4-(trifluoromethyl) phenyl)-1,2,3,4-tetrahydroisoquinoline (3c).[5] Starting from 1 mmol of 1-iodo-4-(trifluoromethyl)benzene (section 2.4). The product was purified by column chromatography on silica gel eluting with hexane/EtOAc (90:10). White solid was obtained in 35% yield (97 mg, 0.35 mmol). **Melting point**: 76.6-77.7 °C. **IR (KBr):** ν_{max} 3069, 3007, 2929, 2849, 1611, 1526, 1469, 1449, 1396, 1330, 1296, 1230, 1162, 1102, 1071, 934, 820, 755, 655, 591, 512. **^{1}H NMR** (400 MHz, $CDCl_3$) δ 7.50 (d, *J* = 8.5 Hz, 2H), 7.23 – 7.16 (m, 4H), 6.93 (d, *J* = 8.5 Hz, 2H), 4.48 (s, 2H), 3.63 (t, *J* = 5.9 Hz, 2H), 2.99 (t, *J* = 5.9 Hz, 2H). **^{13}C NMR {1H}** (101 MHz, CDCl3) δ 152.2, 134.9, 133.8, 128.3, 126.7, 126.5, 126.48 (q, *J*= 4.0 Hz), 126.3, 122.3 (q, *J*= 271.7 Hz), 119.1 (q, *J*= 32.0 Hz),113.0, 49.5, 45.2, 29.0. **GC-MS (EI):** m/z 278 (M^+ +1, 12), 277 (M^+, 68), 276 (72), 145 (17), 115 (10), 104 (100), 103 (20), 78 (22), 77 (14).

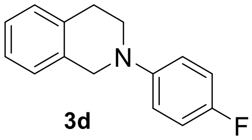

3d

2-(4-fluorophenyl)-1,2,3,4-tetrahydroisoquinoline (3d).[16] Starting from 1 mmol of 1-fluoro-4-iodobenzene (section 2.4). The product was purified by column chromatography on silica gel eluting with hexane/EtOAc (90:10). White solid was obtained in 34% yield (77 mg, 0.34 mmol). **Melting point**: 79.8-80.4°C. **IR (KBr):** ν_{max} 2925, 1508, 1455, 1385, 1267, 1227, 1207, 1154,1106, 1052, 932, 813, 754, 531. **^{1}H NMR** (400 MHz, $CDCl_3$) δ 7.23 – 7.11 (m, 4H), 7.03 – 6.9 (m, 4H), 4.33 (s, 2H), 3.48 (t, *J* = 5.86 Hz, 2H), 2.98 (t, *J* = 5.86 Hz, 2H). **^{13}C NMR {^{1}H}** (101 MHz, $CDCl_3$) δ 156.6 (d, *J* = 238.0 Hz), 147.2 (d, *J* = 2.0 Hz), 134.4, 134.1, 128.5, 126.3, 126.2, 125.9, 117.0 (d, *J* = 7.5 Hz), 115.4 (d, *J* = 22.0 Hz), 51.7, 47.6, 28.9. **GC-MS (EI):** *m/z* 228 (M^+ + 1,14), 227 (M^+, 89), 226 (87), 122 (14), 115 (12), 105 (14), 104 (100), 103 (27), 95 (27), 78 (23), 77 (17), 75 (13).

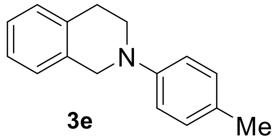

3e

2-(*p*-tolyl)-1,2,3,4-tetrahydroisoquinoline (3e).[16] Starting from 1 mmol of 1-iodo-4-methybenzene (section 2.4). The product was purified by column chromatography on silica gel eluting with hexane/EtOAc (90:10). Yellow oil was obtained in 36% yield (80.0 mg, 0.36 mmol). **IR (KBr):** ν_{max} 2920, 1515, 1464, 1385, 1331, 1288, 1267, 1233, 1210, 1150, 1111, 1053, 1037,1020, 931, 804, 749, 529. **^{1}H NMR** (400 MHz, $CDCl_3$) δ 7.20 – 7.07 (m, 6H), 6.94 – 6.89 (m, 2H), 4.35 (s, 2H), 3.51 (t, *J* = 5.9 Hz, 2H), 2.98 (t, *J* = 5.8 Hz, 2H), 2.28 (s, 3H). **^{13}C NMR {^{1}H}** (101 MHz, $CDCl_3$) δ 148.7, 134.8, 134.6, 129.7, 128.6, 128.4, 126.5, 126.3, 125.9, 115.9, 51.5, 47.3, 29.1, 20.4. **GC-MS (EI):** *m/z* 224 (M^+ + 1, 9), 223 (M^+, 71), 222 (79), 119 (16), 118 (19), 115 (12), 105 (17), 104 (100), 103 (34), 91 (57), 89 (14), 78 (48), 77 (31), 65 (40), 63 (15), 51 (17).

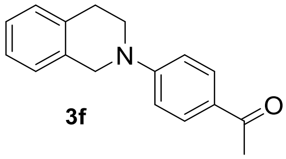

3f

1-(4-(3,4-dihydroisoquinolin-2(1H)-yl)phenyl)ethan-1-one (3f).[14] Starting from 1 mmol of 4-iodoacetophenone (section 2.4). The product was purified by column chromatography on silica gel eluting with hexane/EtOAc (70:30). Yellow solid was obtained in 40% yield (100 mg, 0.40 mmol). **Melting point:** 97.5-96.9°C. **IR (KBr):** ν_{max} 2995, 2911, 2857, 2823, 1655, 1600, 1584,1518, 1426, 1388, 1360, 1266, 1230, 1186, 1155, 985, 814, 750, 617, 590, 508. **^{1}H NMR** (400 MHz, $CDCl_3$) δ 7.91 (d, *J* = 9.1 Hz, 2H), 7.25 – 7.16 (m, 4H), 6.88 (d, *J* = 9.1 Hz, 2H), 4.54 (s, 2H), 3.67 (t, *J* = 5.9 Hz, 2H), 3.00 (t, *J* = 5.9 Hz, 2H), 2.53 (s, 3H). **^{13}C NMR {^{1}H}** (101 MHz,$CDCl_3$) δ 196.4, 153.1, 135.0, 133.7, 130.6, 128.2, 126.8, 126.5, 126.4, 111.9, 49.0, 44.7, 29.0, 26.1. **GC-MS (EI):** m/z 252 (M^+ + 1, 8) 251 (M^+, 36), 250 (33), 118 (15), 115 (12), 105 (11), 104 (100), 103 (19), 102 (12), 91 (13), 78 (33), 77 (35), 51 (13).

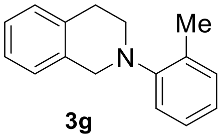

3g

2-(*o*-tolyl)-1,2,3,4-tetrahydroisoquinoline (3g).[17] Starting from 1 mmol of 1-iodo-2-methylbenzene (section 2.4). The product was purified by column chromatography on silica gel

eluting with hexane/EtOAc (90:10). White solid was obtained in 32% yield (72 mg, 0.32 mmol). **Melting point:** 97.5-96.9°C. **IR (KBr):** v_{max} 2995, 2911, 2857, 2823, 1655, 1600, 1584,1518, 1426, 1388, 1360, 1266, 1230, 1186, 1155, 985, 814, 750, 617, 590, 508. **^{1}H NMR** (400 MHz, $CDCl_3$) δ 7.23 – 7.13 (m, 5H), 7.09 (ddd, *J* = 9.8, 7.9, 2.3 Hz, 2H), 7.01 (td, *J* = 7.4, 1.4 Hz, 1H), 4.13 (d, *J* = 1.2 Hz, 2H), 3.22 (t, *J* = 5.8 Hz, 2H), 3.01 (t, *J* = 5.8 Hz, 2H), 2.35 (s, 3H). **^{13}C NMR {1H}** (101 MHz, $CDCl_3$) δ 151.7, 135.6, 134.7, 133.0, 131.3, 129.1,126.7, 126.5, 126.3, 125.8, 123.3, 119.4, 54.3, 50.4, 29.8, 18.2. **GC-MS (EI):** *m/z* 224 (M^+ +1, 9), 223 (M^+, 54), 222 (70), 118 (45), 117 (12), 115 (12), 105 (20), 104 (100), 103 (29), 91 (56), 89 (17), 78 (39), 77 (26), 65 (42), 63 (12), 51 (21).

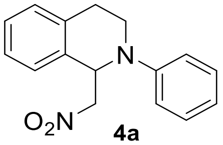

4a

1-(nitromethyl)-2-phenyl-1,2,3,4-tetrahydroisoquinoline (4a).[16] Starting from 0.25 mmol of **3a** (section 2.5). The product was purified by column chromatography on silica gel eluting with hexane/EtOAc (95:5). Yellow oil. **IR (KBr):** v_{max} 3059, 2977, 2923, 1630, 1598, 1549, 1503,1381, 1327, 1260, 1217, 1114, 1031, 875, 804, 751, 696, 639. **^{1}H NMR** (400 MHz, $CDCl_3$) δ 7.30 – 7.16 (m, 5H), 7.14 – 7.11 (m, 1H), 6.97 (dt, *J* = 7.8, 1.1 Hz, 2H), 6.84 (tt, *J* = 7.3, 1.1 Hz, 1H), 5.54 (t, *J* = 7.2 Hz, 1H), 4.86 (dd, *J* = 11.8, 7.8 Hz, 1H), 4.55 (dd, *J* = 11.8, 6.6 Hz, 1H), 3.71 – 3.55 (m, 2H), 3.08 (ddd, *J* = 16.4, 8.7, 5.7 Hz, 1H), 2.79 (dt, *J* = 16.3, 4.9 Hz, 1H). **^{13}C NMR** (101 MHz, $CDCl_3$) δ 148.6, 135.4, 133.1, 129.6, 129.3, 128.3, 127.1, 126.8, 119.6, 115.3, 78.9, 58.3, 42.2, 26.6. **GC-MS (EI):** *m/z* 269 (M^+ +1,1), 268 (M^+, 8), 209 (18), 208 (100), 115 (14), 104 (15).

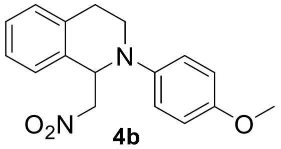

4b

2-(4-methoxyphenyl)-1-(nitromethyl)-1,2,3,4-tetrahydroisoquinoline (4b).[5] Starting from 0.25 mmol of **3b** (section 2.5). The product was purified by column chromatography on silica gel eluting with hexane/EtOAc (70:30). Yellow oil. **IR (KBr):** v_{max} 3061, 2961, 2923, 2833, 1634, 1552, 1510, 1464, 1452, 1427, 1379, 1330, 1286, 1245, 1216, 1184, 1114, 1035, 965, 940, 824, 777, 725, 629, 528. **^{1}H NMR** (400 MHz, $CDCl_3$) δ 7.28 – 7.07 (m, 4H), 6.95 – 6.89 (dt, 2H), 6.85 – 6.79 (dt, 2H), 5.39 (dd, *J* = 8.5, 5.9 Hz, 1H), 4.83 (dd, *J* = 11.9, 8.6 Hz, 1H), 4.56 (dd, *J* = 11.9, 5.8 Hz, 1H), 3.75 (s, 3H), 3.60 – 3.51 (m, 2H), 3.02 (ddd, *J* = 16.2, 9.0, 6.9 Hz,

1H), 2.70 (dt, *J* = 16.6, 4.0 Hz, 1H). **^{13}C NMR** (101 MHz, $CDCl_3$) δ 154.0, 143.1, 135.4, 132.9, 129.5, 127.9, 126.9, 126.6, 118.9, 114.7, 79.0, 58.9, 55.6, 43.2, 25.9. **GC-MS (EI):** *m/z* 299 (M^+ +1, 6), 298 (M^+,5), 239 (32), 238 (100), 237 (21), 236 (18), 118 (13).

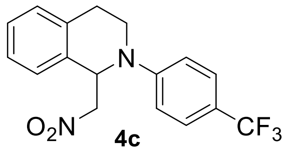

4c

1-(nitromethyl)-2-(4-(trifluoromethyl)phenyl)-1,2,3,4-tetrahydroisoquinoline (4c).[5] Starting from 0.25 mmol of **3c** (section 2.5). The product was purified by column chromatography on silica gel eluting with hexane/EtOAc (80:20). Yellow oil. **IR (KBr):** ν_{max} 2966, 2922, 2854, 1616, 1553, 1524, 1378, 1328, 1240, 1164, 1112, 1072, 1002, 940, 826, 754, 529. **^{1}H NMR** (400 MHz, $CDCl_3$) δ 7.50 (d, *J* = 9,02 Hz, 2H), 7.31-7.18 (m, 3H), 7.16-7.11 (m, 1H), 7.0 (d, *J* = 8.78 Hz, 2H), 5.61 (t, *J* = 7.2 Hz, 1H), 4.86 (dd, *J* = 11.96, 7.64 Hz, 1H), 4.58 (dd, *J* = 11.98, 6.75 Hz, 1H), 3.74-3.6 (m, 2H), 3.16-3.05 (m, 1H), 2.86 (dt, *J* = 16.3, 5.45 Hz, 1H). **^{13}C NMR** (101 MHz, $CDCl_3$) δ 150.5, 134.9, 132.4, 129.2, 128.5, 127.0, 127.0, 126.9 (q, *J* = 3.8 Hz), 120.5 (q, *J* = 32.8 Hz), 113.4 (x 2), 78.5, 57.8, 41.9, 26.6. **^{19}F NMR** (376 MHz, $CDCl_3$) δ -61.35. **GC-MS (EI):** *m/z* 337 (M^+ +1, 2), 336 (M^+, 6), 277 (16), 276 (100), 274 (12), 145 (13), 115 (14).

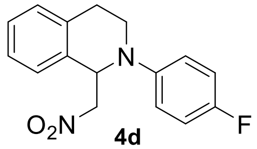

4d

2-(4-fluorophenyl)-1-(nitromethyl)-1,2,3,4-tetrahydroisoquinoline (4d).[16] Starting from 0.25 mmol of **3d** (section 2.5). The product was purified by column chromatography on silica gel eluting with hexane/EtOAc (90:10). Yellow oil. **IR (KBr):** ν_{max} 3002, 2922, 1633, 1550, 1507, 1377, 1240, 1056, 812, 752, 669. **^{1}H NMR** (400 MHz, $CDCl_3$) δ 7.28 – 7.12 (m, 4H), 6.98 – 6.87 (m, 4H), 5.42 (dd, *J* = 8.61, 5,87 Hz, 1H), 4.84 (dd, *J* = 12.01, 8.60 Hz, 1H), 4.57 (dd, *J* = 11,98, 5.87 Hz, 1H), 3.63 – 3.57 (m, 2H), 3.08 – 2.97 (m, 1H), 2.73 (dt, *J* = 16.49, 4.25 Hz, 1H). **^{13}C NMR** (100 MHz, $CDCl_3$) δ 157.4 (d, *J* = 240.4 Hz), 145.5, 135.4, 132.7, 129.6, 128.2, 127.1, 126.9, 118.1 (d, *J* = 8.1 Hz), 116.0 (d, *J* = 22.2 Hz), 79.0, 58.9, 43.0, 26.0. **^{19}F NMR** (376 MHz, $CDCl_3$) δ -124.28. **GC-MS (EI):** *m/z* 287 (M^+ +1, 2), 286 (M^+, 10), 227 (37), 226 (100), 225 (18), 224 (24), 115 (11), 95 (21), 77 (11), 75 (19).

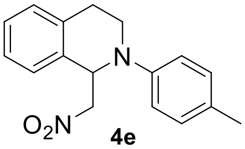

4e

1-(nitromethyl)-2-(*p*-tolyl)-1,2,3,4-tetrahydroisoquinoline (4e).[16] Starting from 0.25 mmol of **3e** (section 2.5). The product was purified by column chromatography on silica gel eluting with hexane/EtOAc (95:5). Yellow oil. **IR (KBr):** ν_{max} 3005, 2996, 2958, 2920, 2851, 1635, 1550, 1513, 1383, 1246, 1098, 1057, 800, 668. **^{1}H NMR** (400 MHz, $CDCl_3$) δ 7.27 – 7.10 (m, 4H), 7.09 – 7.04 (d, 2H), 6.90 – 6.85 (d, 2H), 5.49 (t, *J* = 8.0, 6.4 Hz, 1H), 4.84 (dd, *J* = 11.9, 8.0 Hz, 1H), 4.55 (dd, *J* = 11.8, 6.3 Hz, 1H), 3.68 – 3.52 (m, 2H), 3.06 (ddd, *J* = 15.5, 9.3, 5.8 Hz, 1H), 2.75 (dt, *J* = 16.4, 4.6 Hz, 1H), 2.25 (s, 3H). **^{13}C NMR** (101 MHz, $CDCl_3$) δ 146.4, 135.4, 133.0, 130.0, 129.3, 129.1, 128.2, 127.0, 126.6, 115.9, 78.9, 58.4, 42.3, 26.3, 20.4. **GC-MS (EI):** *m/z* 283 (M^+ +1, 3), 282 (M^+, 6), 223 (21), 222 (100), 115 (12), 91 (25), 65 (10).

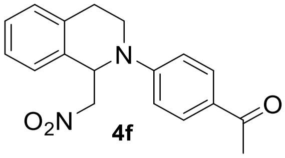

4f

1-(4-(1-(nitromethyl)-3,4-dihydroisoquinolin-2(1H)-yl)phenyl)ethan-1-one (4f).[14] Starting from 0.25 mmol of **3f** (section 2.5). The product was purified by column chromatography on silica gel eluting with hexane/EtOAc (60:40). Yellow oil. **IR (KBr):** ν_{max} 2966, 2922, 1670, 1597, 1550, 1384, 1258, 1103, 1018, 805, 749, 668, 585. **^{1}H NMR** (400 MHz, $CDCl_3$) δ 7.92 (d, *J* = 9.0 Hz, 2H), 7.33 – 7.20 (m, 3H), 7.16 (dd, *J* = 6.3, 2.2 Hz, 1H), 6.98 (d, *J* = 9.0 Hz, 2H), 5.68 (t, *J* = 7.2 Hz, 1H), 4.88 (dd, *J* = 11.9, 7.2 Hz, 1H), 4.59 (dd, *J* = 11.9, 7.2 Hz, 1H), 3.72 (t, *J* = 6.2 Hz, 2H), 3.14 (dt, *J* = 16.1, 6.3 Hz, 1H), 2.92 (dt, *J* = 16.2, 5.9 Hz, 1H), 2.53 (s, 3H). **^{13}C NMR** (101 MHz, $CDCl_3$) δ 196.5, 151.5, 134.8, 132.4, 130.8, 129.1, 128.6, 127.8, 127.10, 127.07, 112.3, 78.4, 57.5, 42.0, 27.0, 26.2. **GC-MS (EI):** *m/z* 311 (M^+ +1, 1), 310 (M^+, 4), 251 (56), 250 (82), 249 (21), 248 (40), 205 (11), 132 (14), 118 (17), 117 (11), 116 (18), 104 (100), 103 (23), 91 (13), 89 (11), 78 (25), 77 (25), 76 (18), 65 (12), 63 (11), 51 (10), 50 (11).

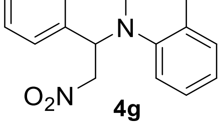

4g

1-(nitromethyl)-2-(*o*-tolyl)-1,2,3,4-tetrahydroisoquinoline (4g).[17] Starting from 0.25 mmol of **3g** (section 2.5). The product was purified by column chromatography on silica gel eluting with hexane/EtOAc (95:5). Yellow oil. **IR (KBr):** ν_{max} 2950, 2923, 2853, 1653, 1589, 1555, 1492, 1460, 1453, 1378, 1262, 1108, 1006, 801, 778, 164, 751, 734, 655. **^{1}H NMR** (400 MHz, $CDCl_3$) δ 7.29 – 7.26 (m, 1H), 7.26 – 7.25 (m, 1H), 7.19 (ddd, *J* = 8.7, 4.7, 2.9 Hz, 3H), 7.03 – 6.96 (m, 2H), 6.75 – 6.69 (m, 1H), 5.14 (dd, *J* = 10.4, 4.5 Hz, 1H), 4.82 (dd, *J* = 12.0, 10.4 Hz, 1H), 4.60 (dd, *J* = 12.0, 4.5 Hz, 1H), 3.50 (ddd, *J* = 14.0, 11.9, 3.8 Hz, 1H), 3.26 – 3.17 (m, 1H), 2.84 (ddd, *J* = 17.2, 11.9, 5.6 Hz, 1H), 2.55 (ddd, *J* = 16.7, 3.9, 2.1 Hz, 1H), 2.27 (s, 3H). **^{13}C NMR** (101 MHz, $CDCl_3$) δ 149.1, 136.3, 134.2, 133.3, 131.3, 129.8, 127.6, 126.7, 126.6, 124.4, 122.9, 79.5, 59.7, 43.1, 24.6, 17.7. **GC-MS (EI):** *m/z* 283 (M^+ +1, 2), 282 (M^+, 5), 223 (16), 222 (100), 221 (15), 220 (12), 217 (12), 147 (11), 118 (11), 115 (11), 103 (16), 96 (18), 91 (33), 77 (12), 65 (36), 51 (10).

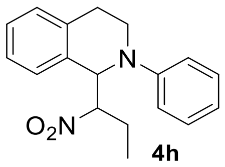

4h

1-(1-nitropropyl)-2-phenyl-1,2,3,4-tetrahydroisoquinoline (4h).[16] Starting from 0.25 mmol of **3a** (section 2.5). The product was purified by column chromatography on silica gel eluting with hexane/EtOAc (80:20). Yellow oil. **IR (KBr):** ν_{max} 2971, 2914, 2360, 1598, 1546, 1493,1370, 1319, 1269, 1212, 933, 809, 749. **^{1}H NMR** (400 MHz, $CDCl_3$) δ diastereomeric mixture at a 1:0.5 ratio (major : minor isomers, diastereomeric ratio determined from the reaction crude) 7.35 – 7.09 (m, 11H, mixture of isomers), 7.05 – 6.89 (m, 4H, mixture of isomers), 6.80 (dtt, *J* = 11.4, 7.3, 1.0 Hz, 3H, minor isomer), 5.24 (d, *J* = 9.3 Hz, 1H, minor isomer), 5.13 (d, *J* = 9.5 Hz, 1H, major isomer), 4.86 (ddd, *J* = 11.5, 9.5, 3.1 Hz, 1H, major isomer), 4.68 (ddd, *J* = 11.4, 9.3, 3.2 Hz, 1H, minor isomer), 3.85 (ddd, *J* = 13.7, 9.3, 5.6 Hz, 1H, major isomer), 3.67 (dddd, *J* = 13.9, 6.2, 4.9, 1.0 Hz, 1H, major isomer), 3.63 – 3.48 (m, 2H, minor isomer), 3.07 (ddd, *J* = 16.2, 9.7, 6.3 Hz, 2H, mixture of isomers), 2.89 (tt, *J* = 16.9, 5.3 Hz, 2H, mixture of isomers), 2.29 – 2.02 (m, 2H, major isomer), 1.83 (dqd, *J* = 14.8, 7.5, 3.1 Hz, 2H, minor isomer), 0.94 (t, *J* = 7.3 Hz, 6H, mixture of isomers). **^{13}C NMR** (101 MHz, $CDCl_3$) δ 149.1, 149.0, 135.6, 134.7, 134.0, 132.6, 129.4, 129.3, 129.2, 128.7, 128.6, 128.3, 128.2, 127.3, 126.7, 125.9, 119.4, 118.6, 115.9, 114.2, 96.2, 93.1, 62.2, 60.7, 43.6, 42.4, 26.8, 25.8, 25.0, 24.6, 10.7 (x 2).

9,10-dimethyl-9,10-dihydro-9,10-epidioxyanthracene (**DMA-O_2**). **^{1}H NMR** (400 MHz, $CDCl_3$) δ 7.39 (dd, *J* = 5.5, 3.2 Hz, 4H), 7.28 (dd, *J* = 5.5, 3.2 Hz, 4H), 2.15 (s, 6H). **^{13}C NMR** (101 MHz, $CDCl_3$) δ 141.0, 127.5, 120.8, 79.7, 13.9.

11. NMR spectra of compounds 2a-h, 3a-g, 4a-h and DMA-O_2

^{1}H NMR (400 MHz, $CDCl_3$). Benzonitrile (**2a**).

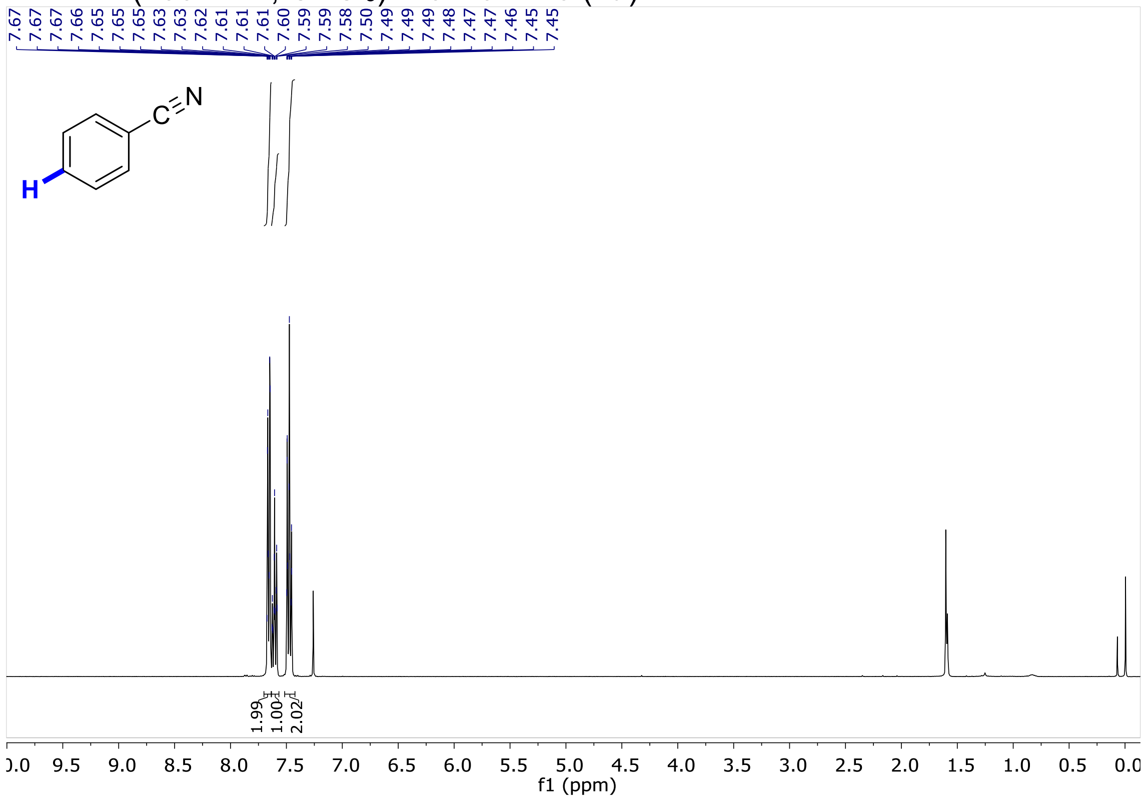

^{13}C NMR {^{1}H} (101 MHz, $CDCl_3$). Benzonitrile (**2a**).

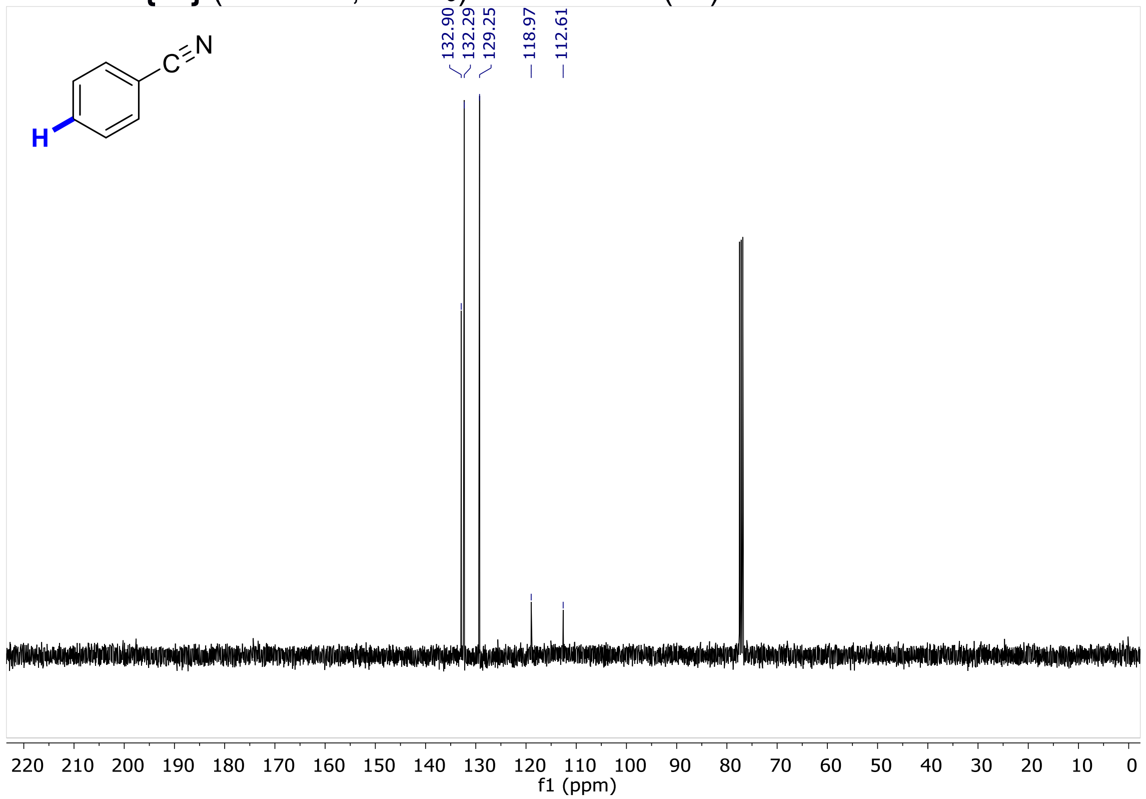

^{1}H NMR (400 MHz, $CDCl_3$). Acetophenone (**2b**).

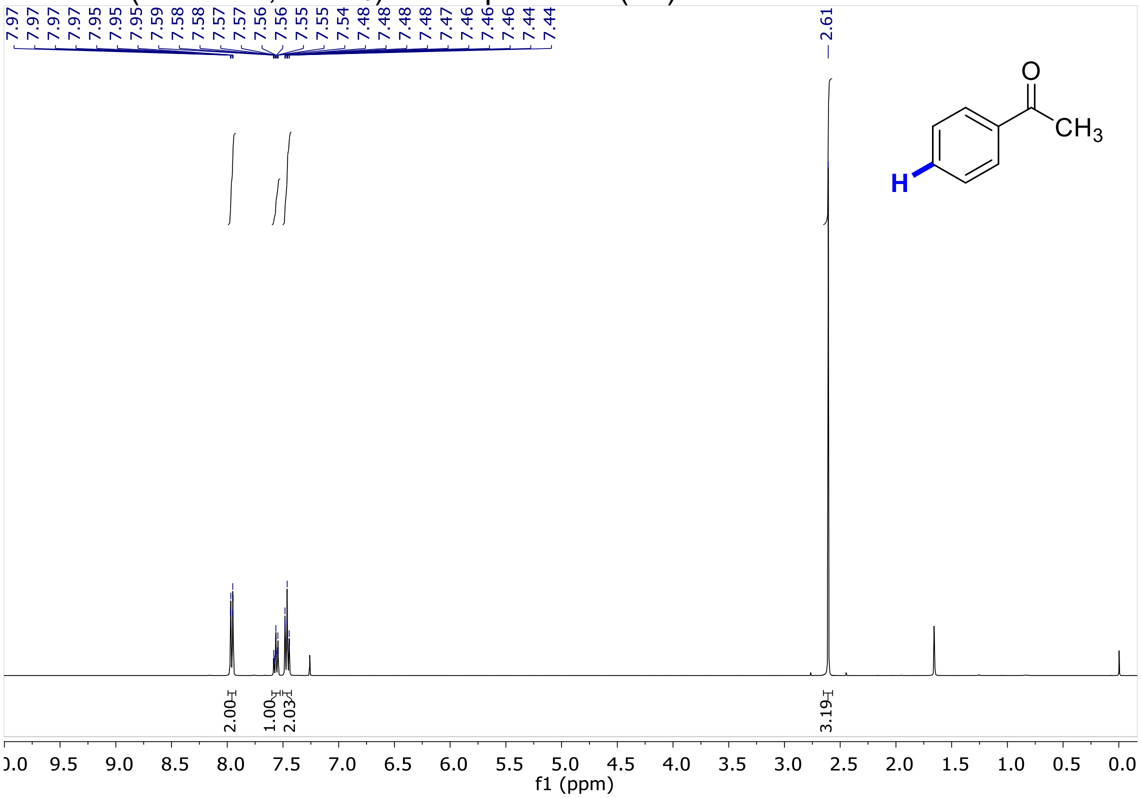

^{13}C NMR {^{1}H} (101 MHz, $CDCl_3$). Acetophenone (**2b**).

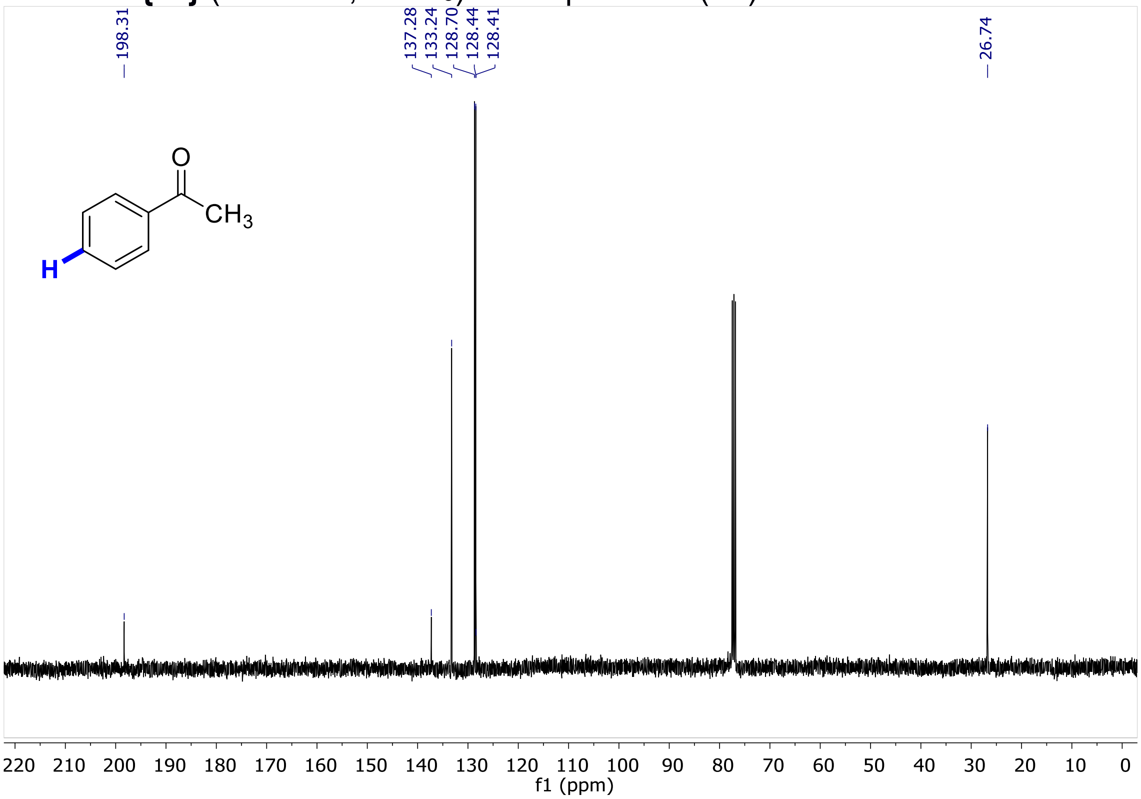

^{1}H NMR (400 MHz, $CDCl_3$). Naphtalene (**2c**)

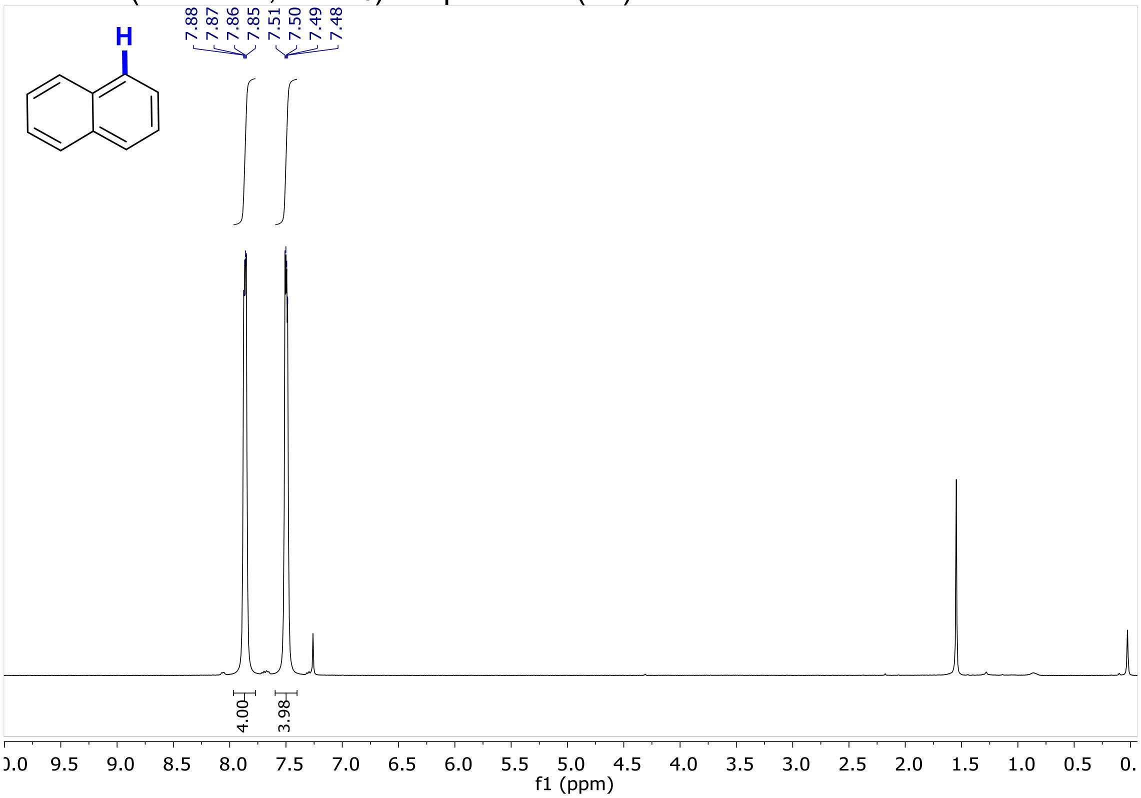

^{13}C NMR {^{1}H} (101 MHz, $CDCl_3$). Naphtalene (**2c**)

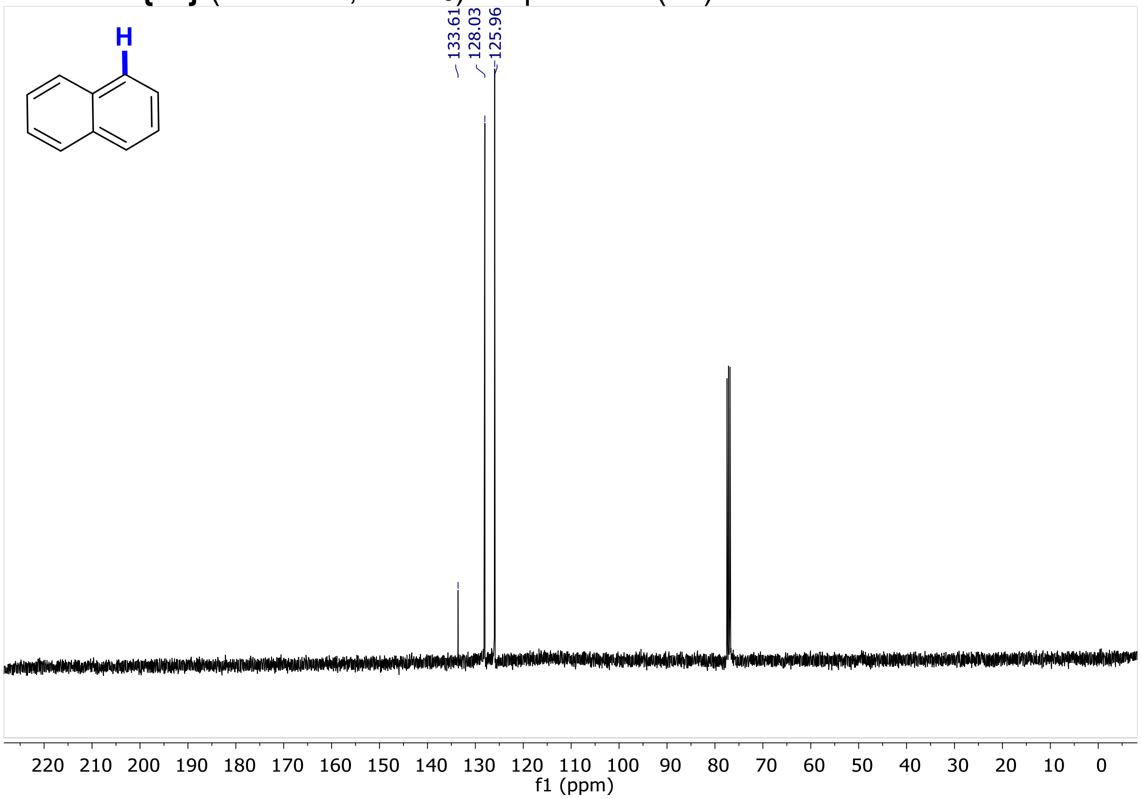

^{1}H NMR (400 MHz, $CDCl_3$). Pyridine (**2d**)

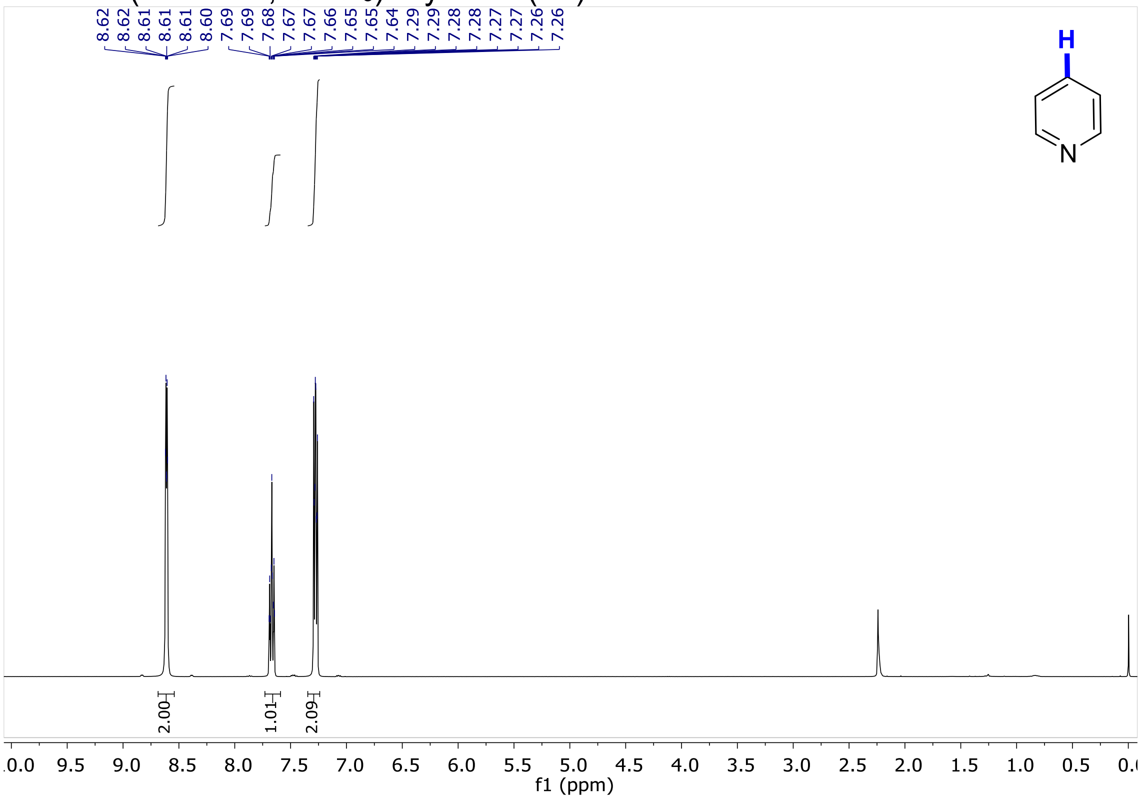

^{13}C NMR {^{1}H} (101 MHz, $CDCl_3$). Pyridine (**2d**)

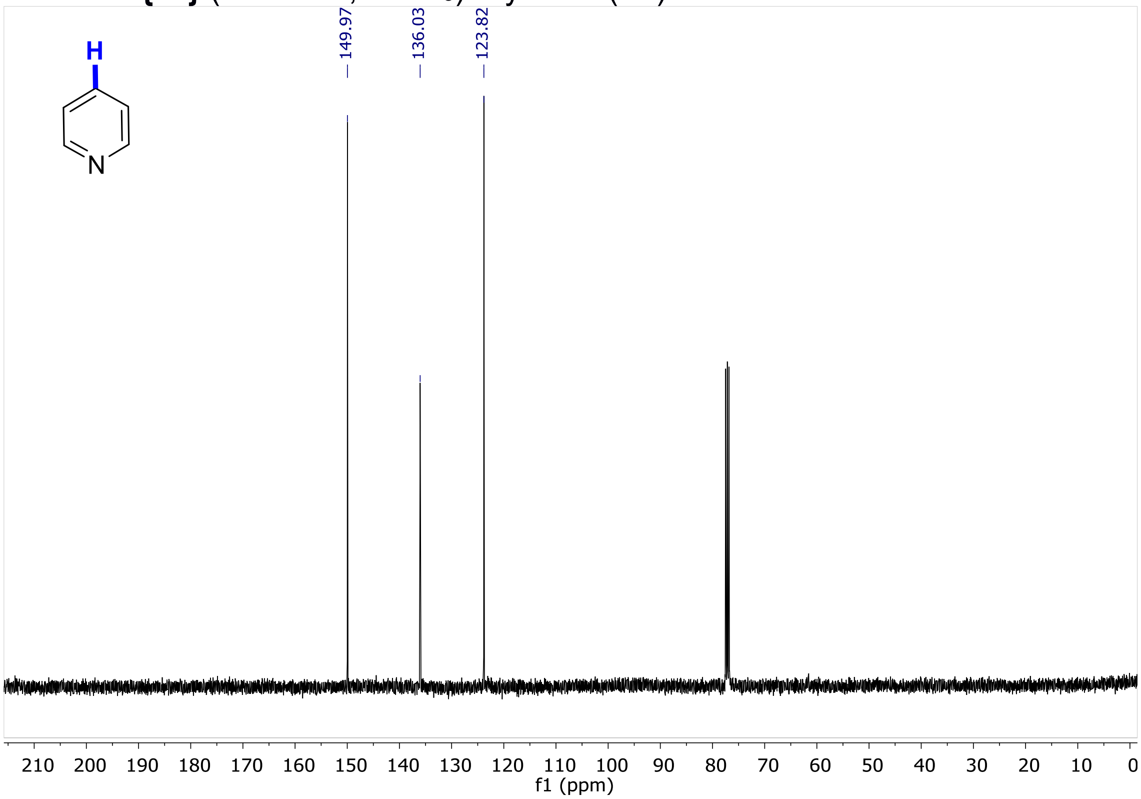

^{1}H NMR (400 MHz, $CDCl_3$). Toluene (**2e**)

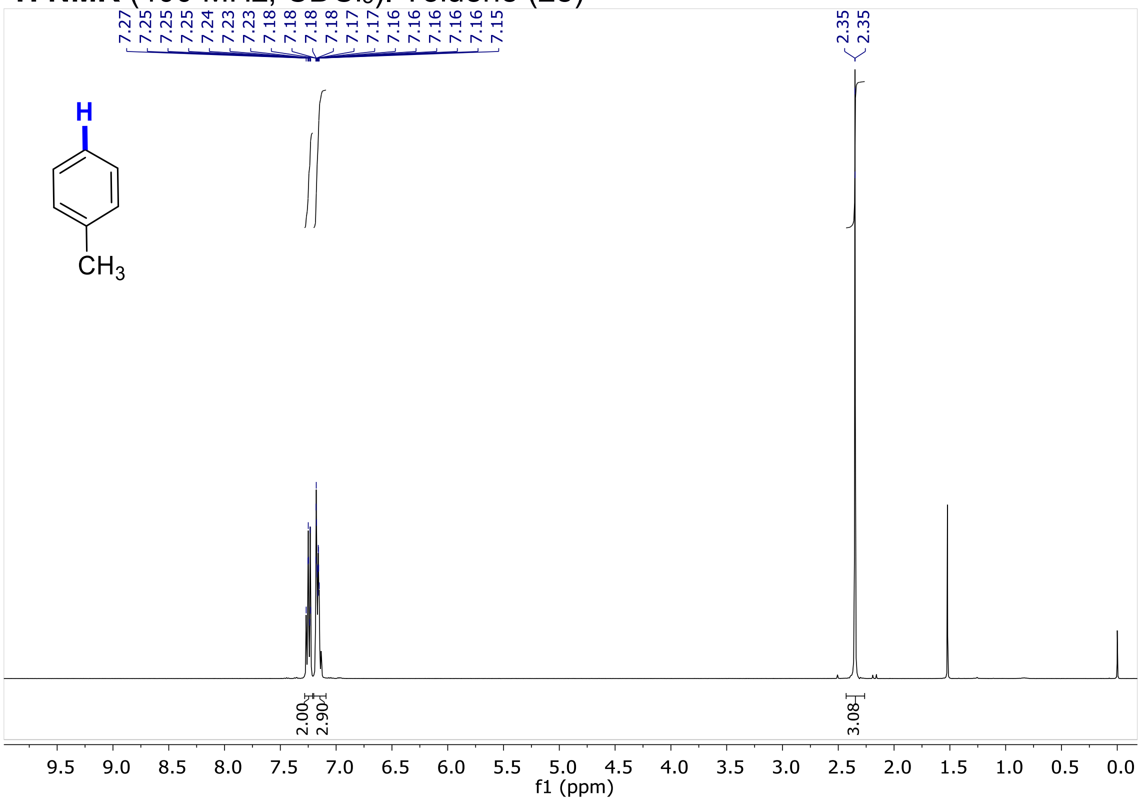

^{13}C NMR {^{1}H} (101 MHz, $CDCl_3$). Toluene (**2e**)

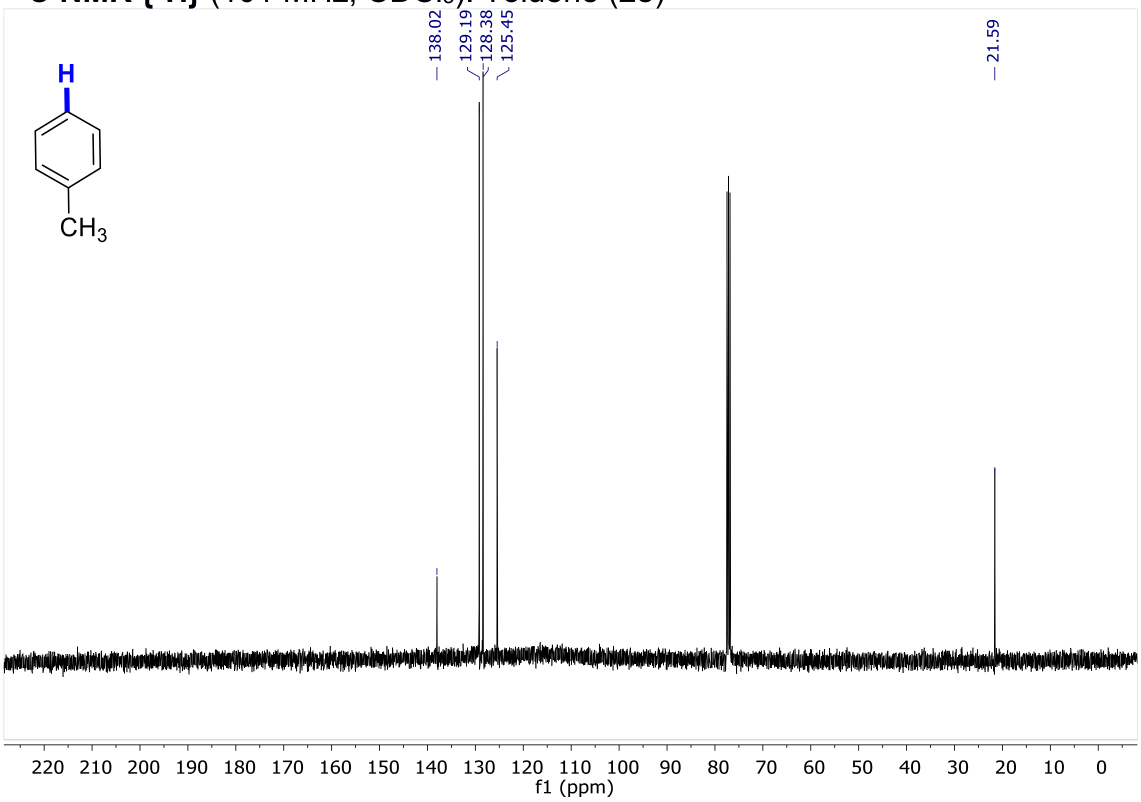

^{1}H NMR (400 MHz, $CDCl_3$). Anisole (**2h**)

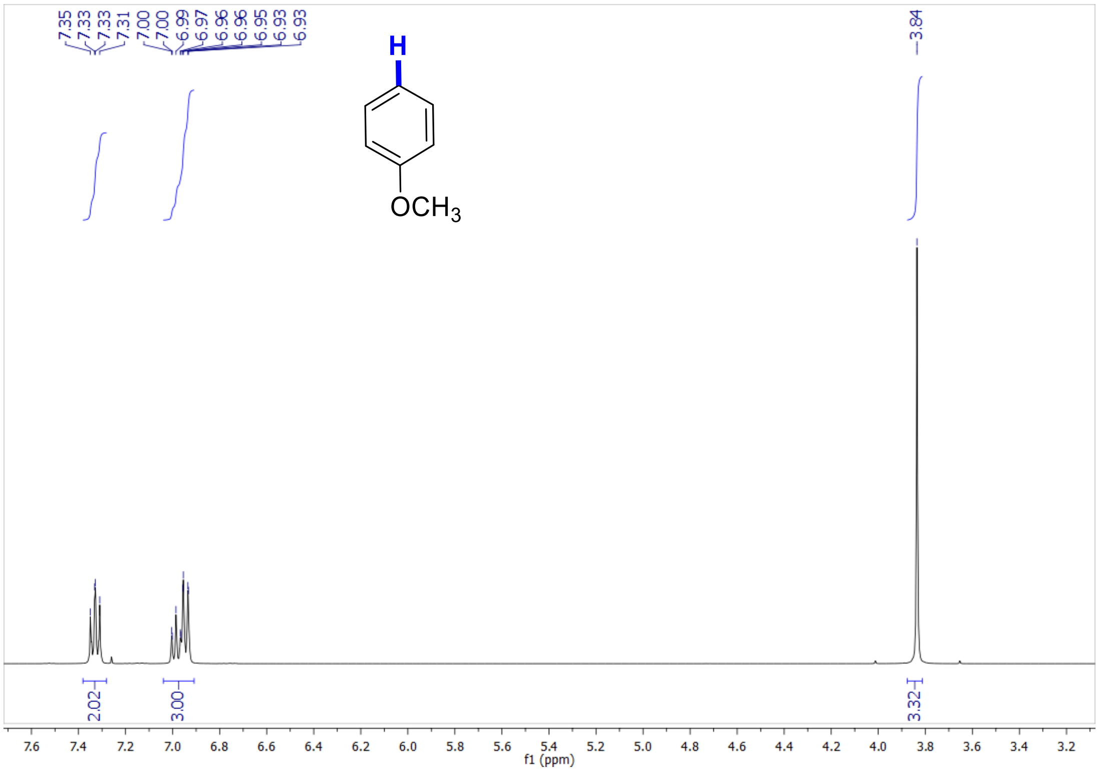

^{13}C NMR {^{1}H} (101 MHz, $CDCl_3$). Anisole (**2h**)

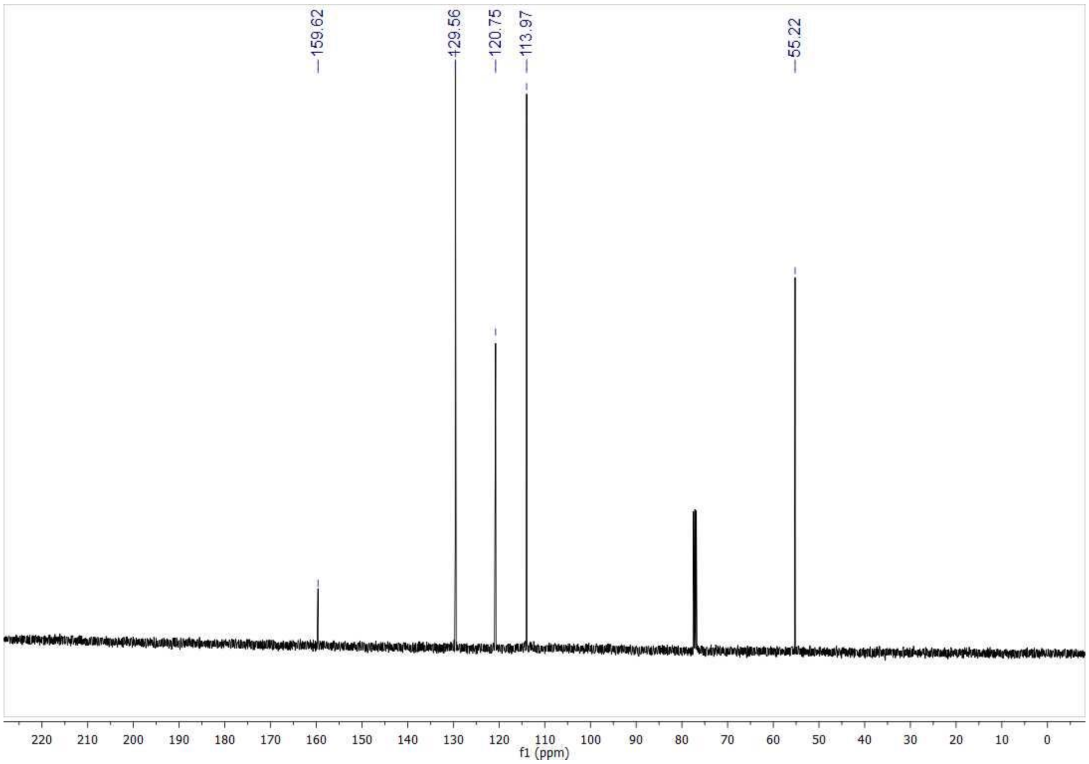

^{1}H NMR (400 MHz, $CDCl_3$). 2-phenyl-1,2,3,4-tetrahydroisoquinoline (**3a**)

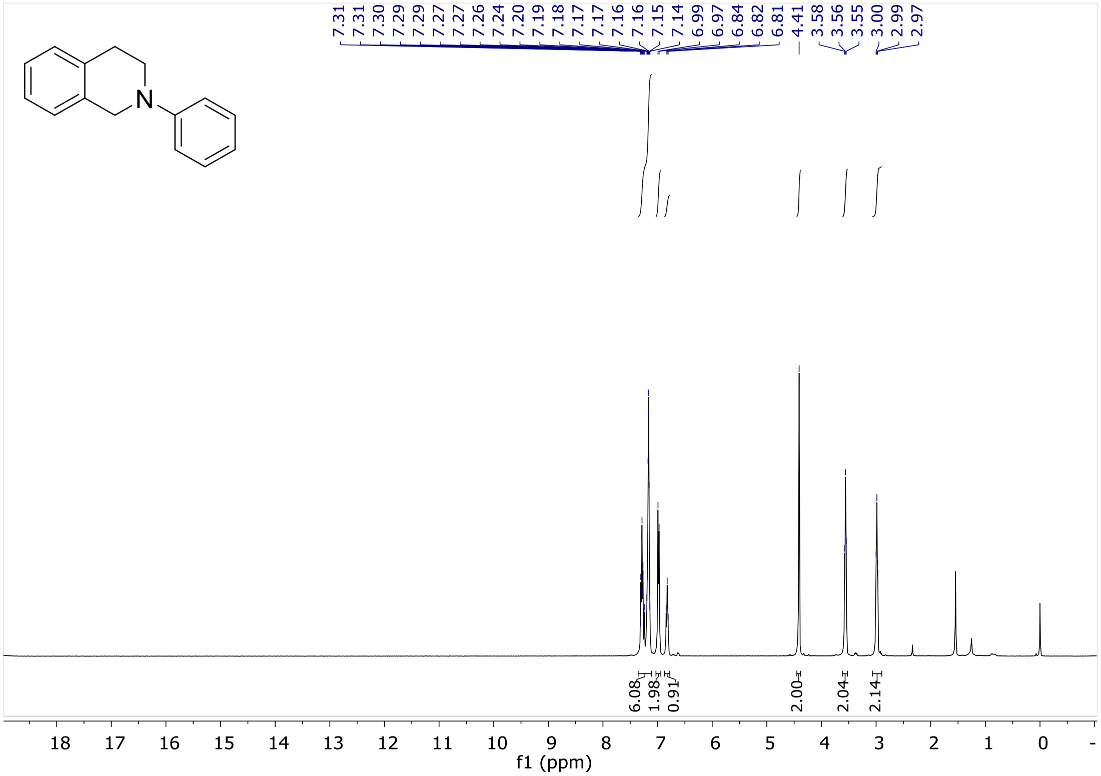

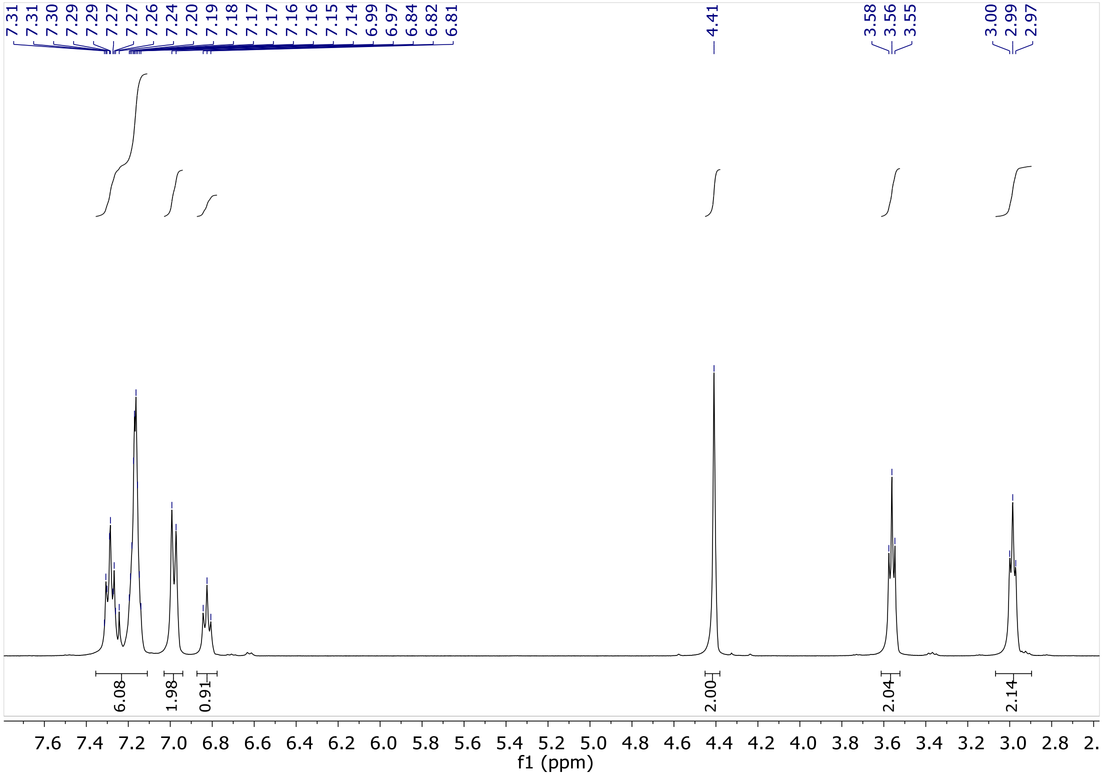

^{13}C NMR {^{1}H} (101 MHz, $CDCl_3$). 2-phenyl-1,2,3,4-tetrahydroisoquinoline (**3a**)

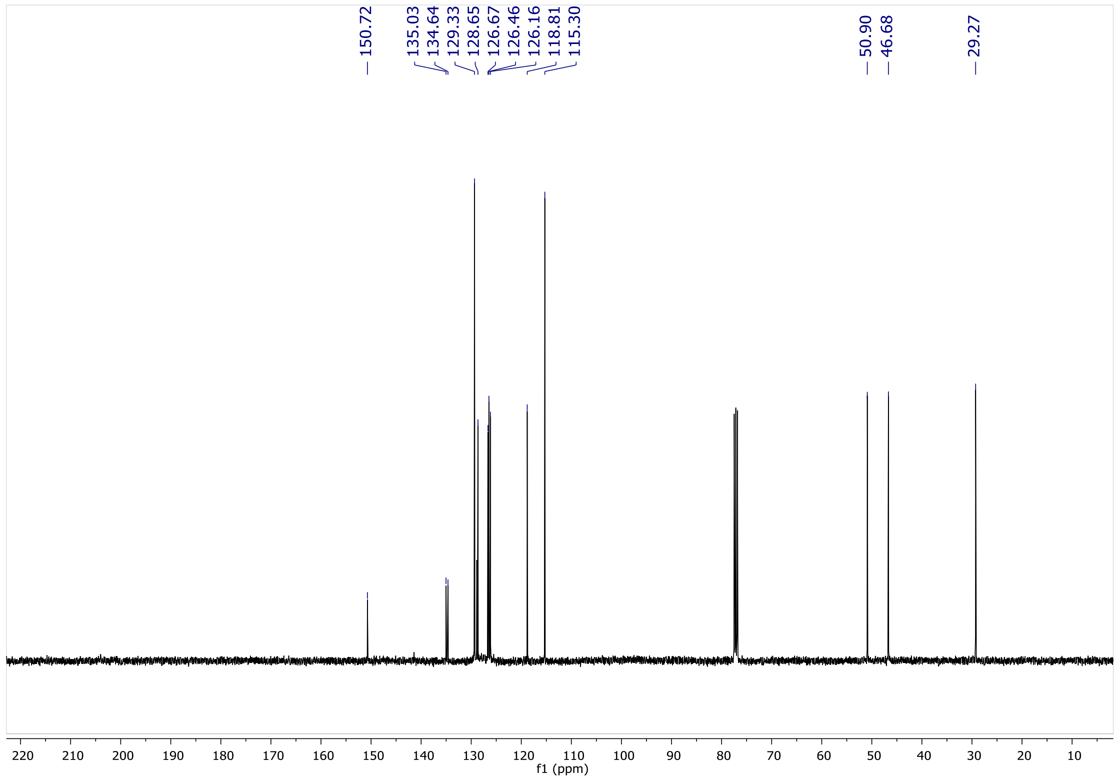

^{1}H NMR (400 MHz, $CDCl_3$).2-(4-methoxyphenyl)-1,2,3,4-tetrahydroisoquinoline (**3b**)

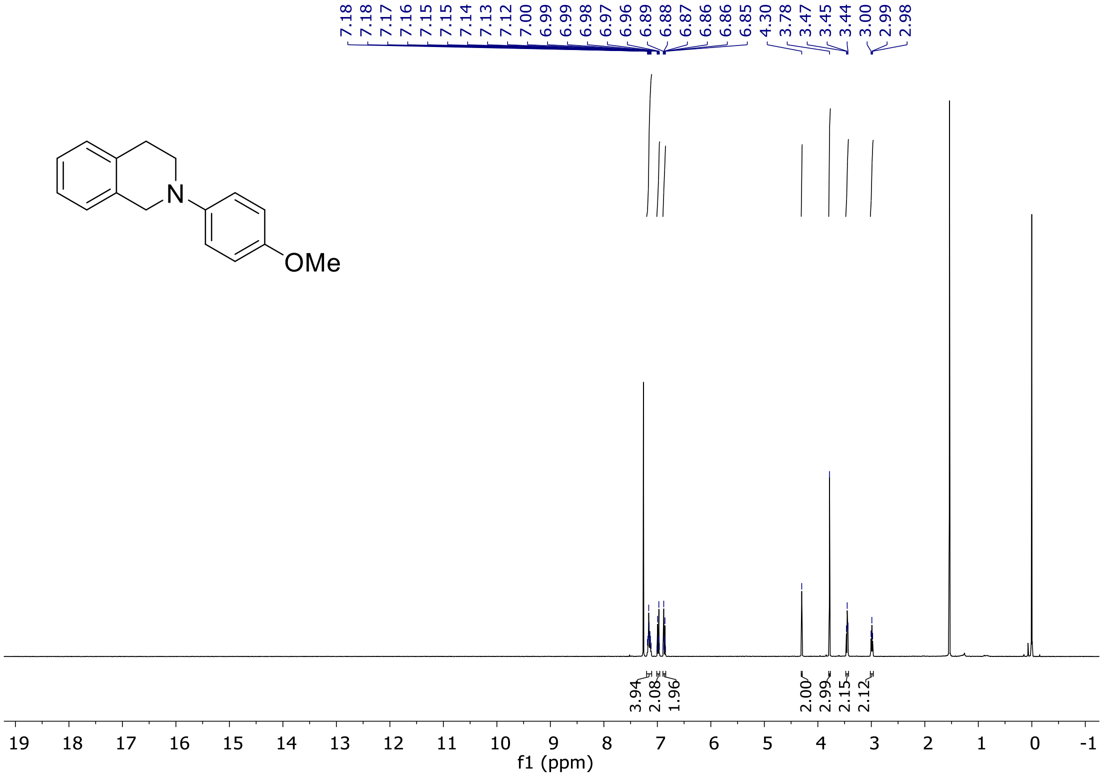

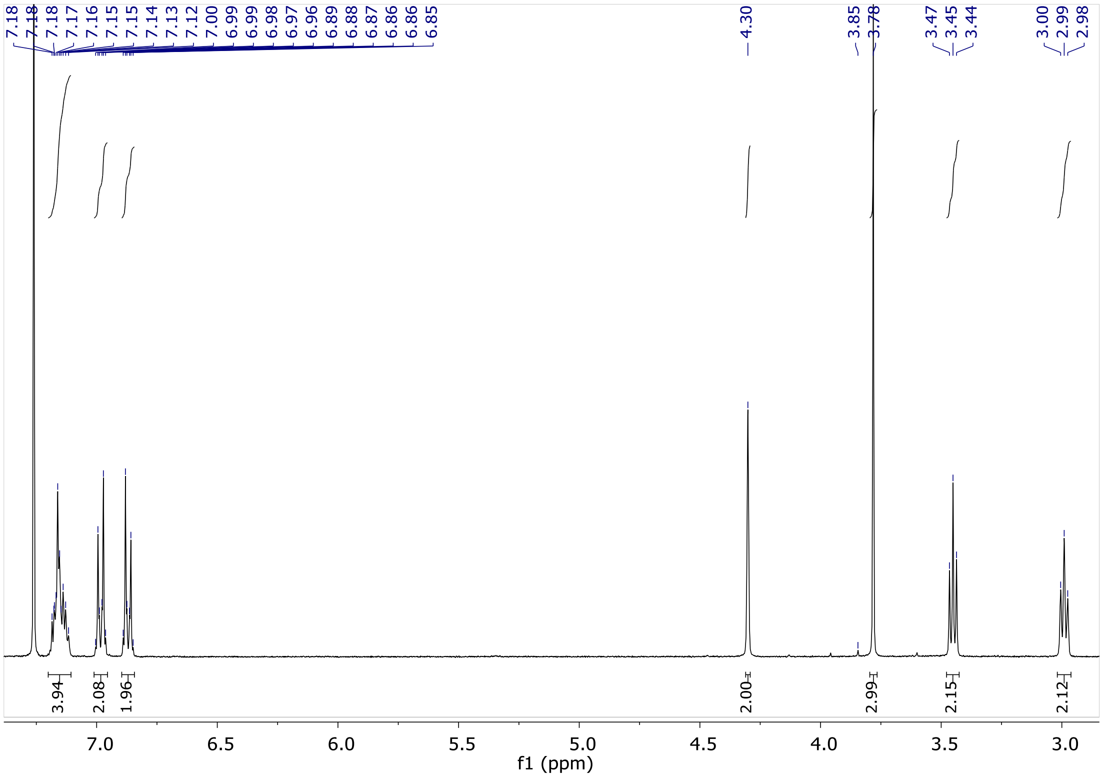

^{13}**C NMR {^{1}H}** (101 MHz, $CDCl_3$). 2-(4-methoxyphenyl)-1,2,3,4-tetrahydroisoquinoline (**3b**)

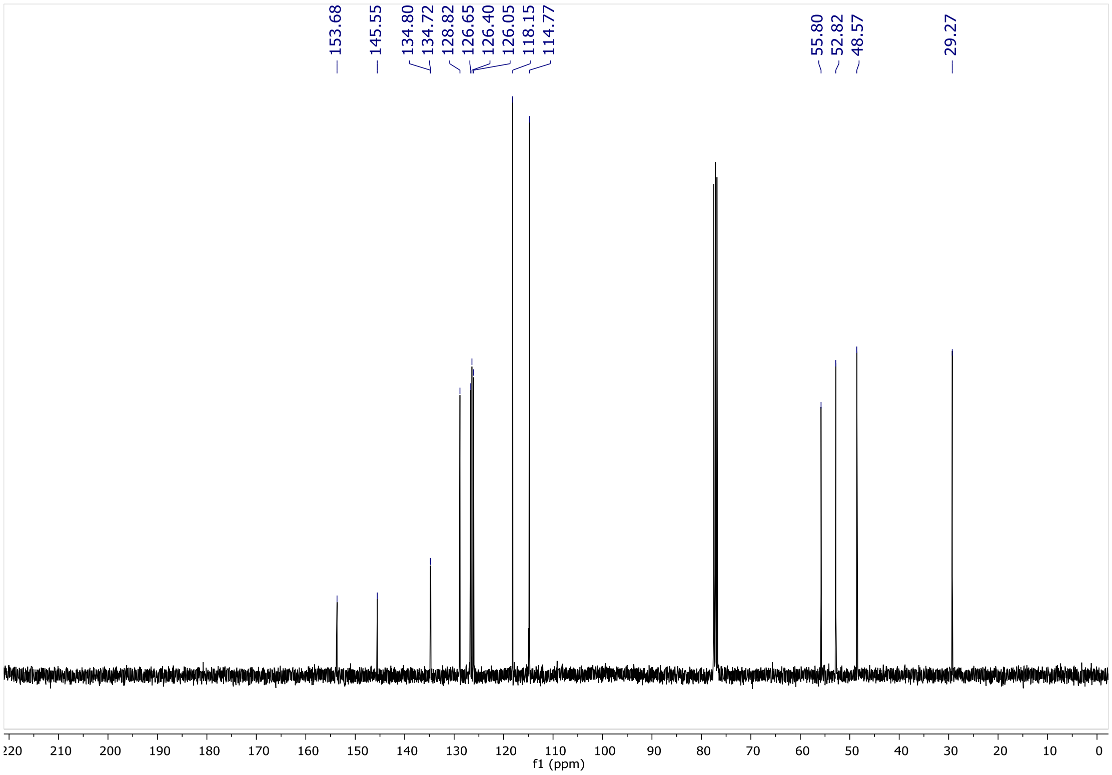

^{1}H NMR (400 MHz, $CDCl_3$). 2-(4-(trifluoromethyl) phenyl)-1,2,3,4-tetrahydroisoquinoline (**3c**)

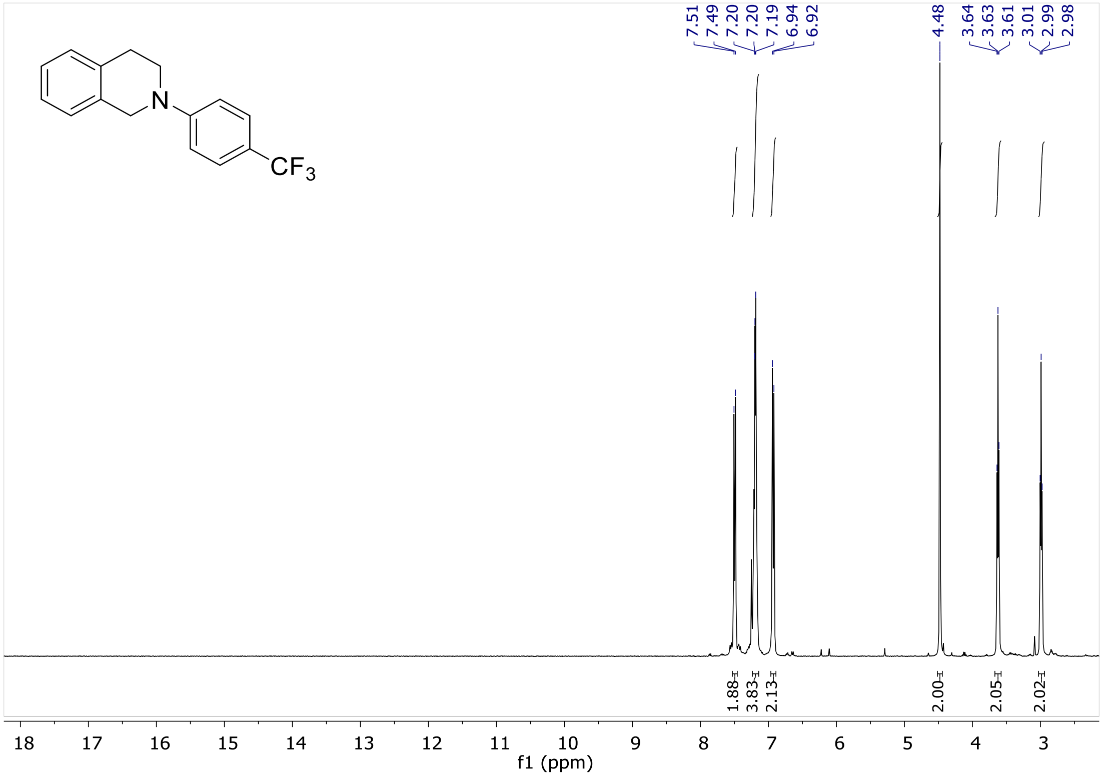

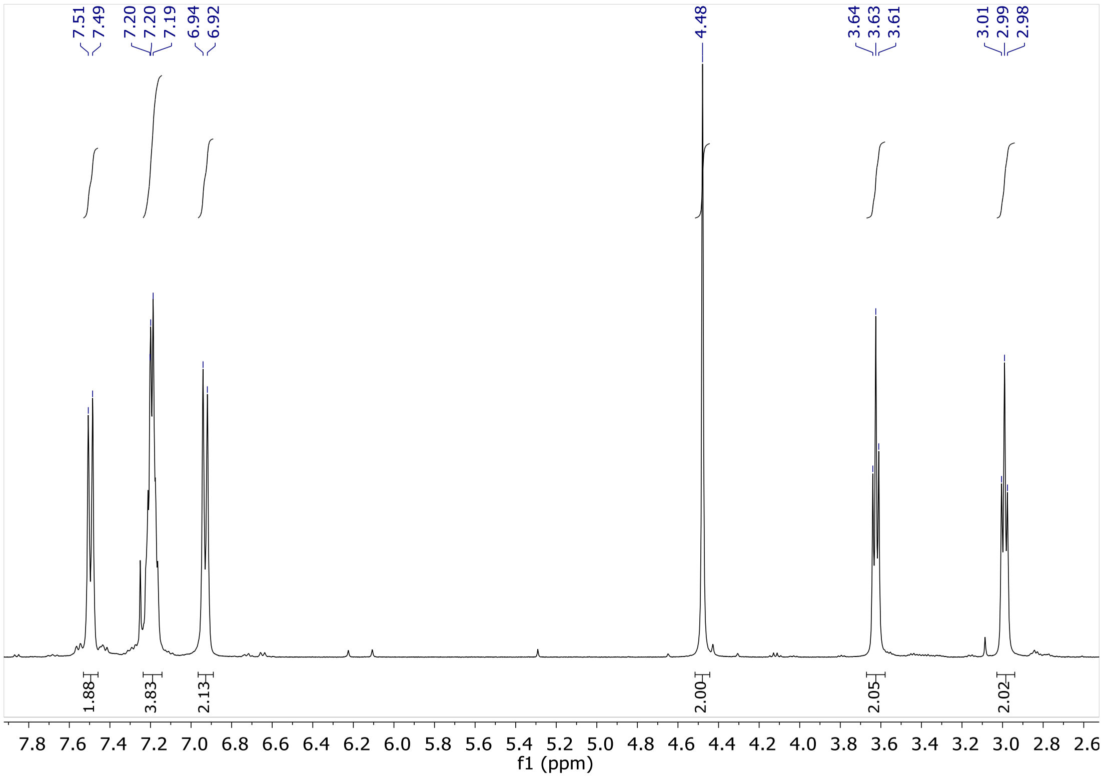

^{13}C NMR {^{1}H} (101 MHz, $CDCl_3$). 2-(4-(trifluoromethyl) phenyl)-1,2,3,4-tetrahydroisoquinoline (**3c**)

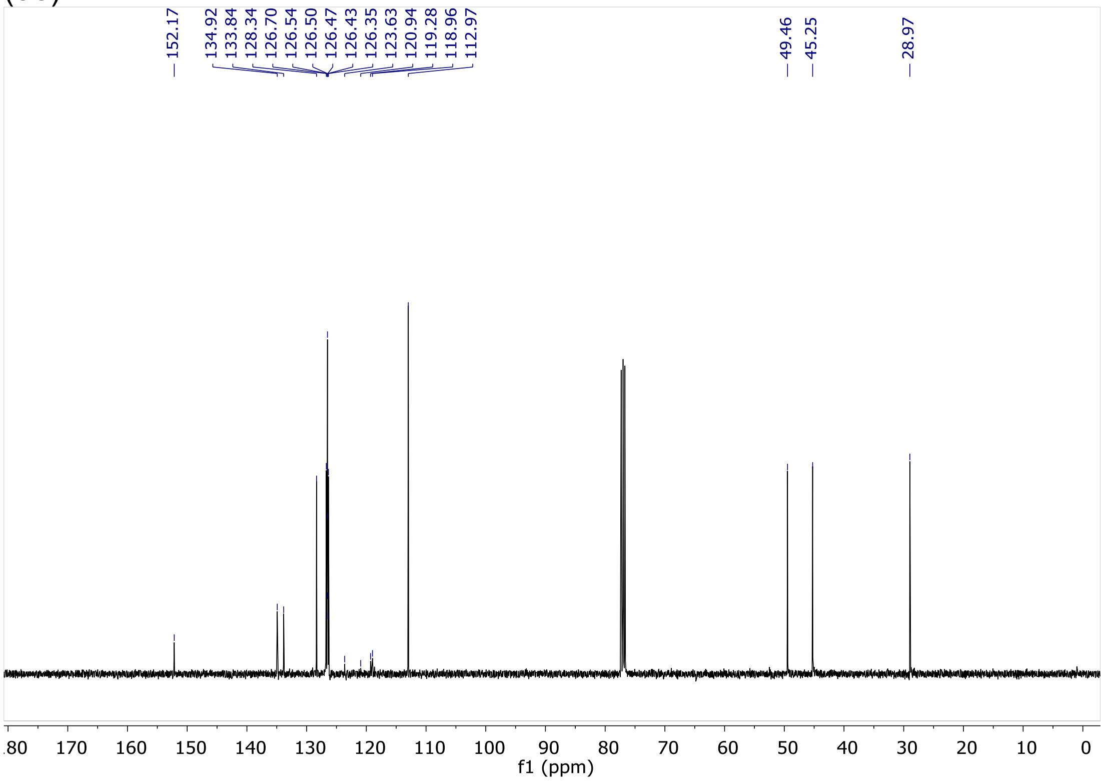

^{1}H NMR (400 MHz, $CDCl_3$). 2-(4-fluorophenyl)-1,2,3,4-tetrahydroisoquinoline (**3d**)

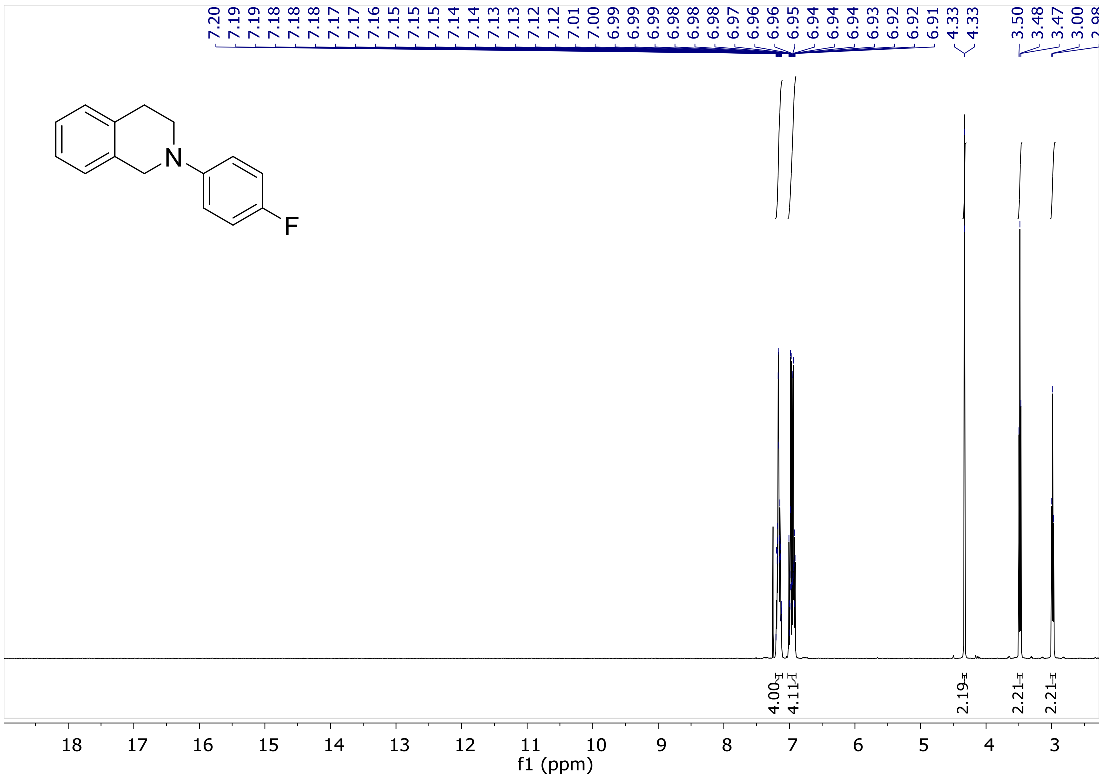

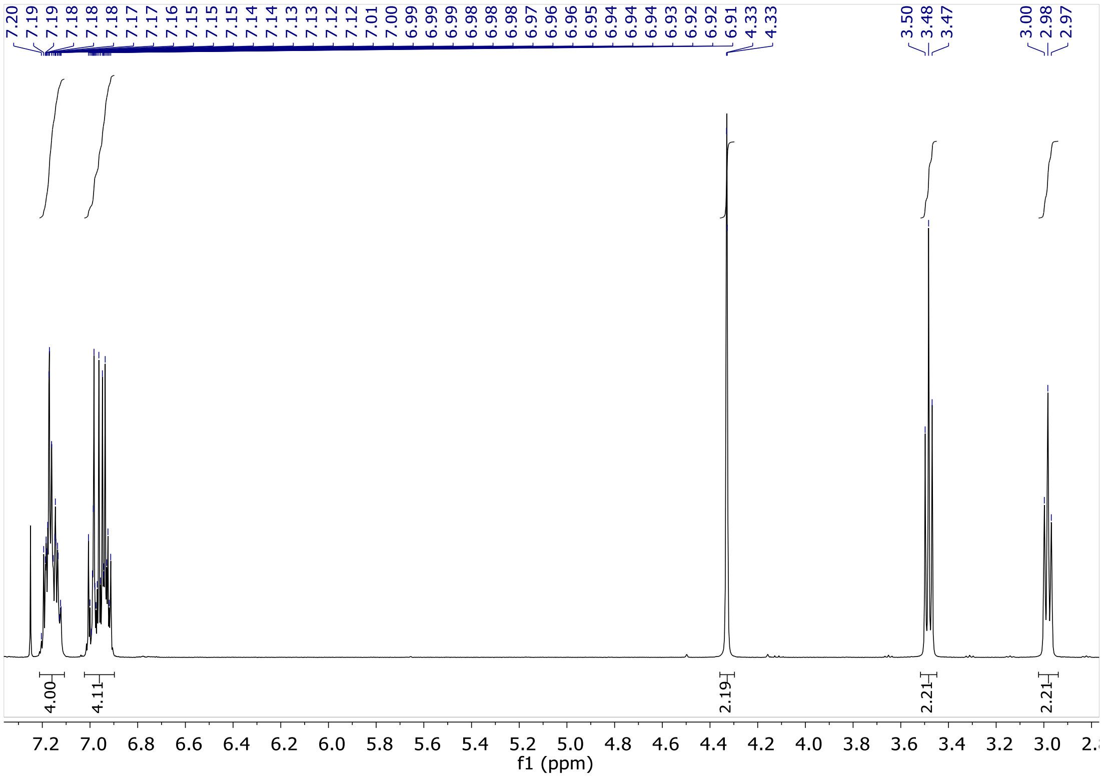

^{13}C NMR {^{1}H} (101 MHz, $CDCl_3$). 2-(4-fluorophenyl)-1,2,3,4-tetrahydroisoquinoline (**3d**)

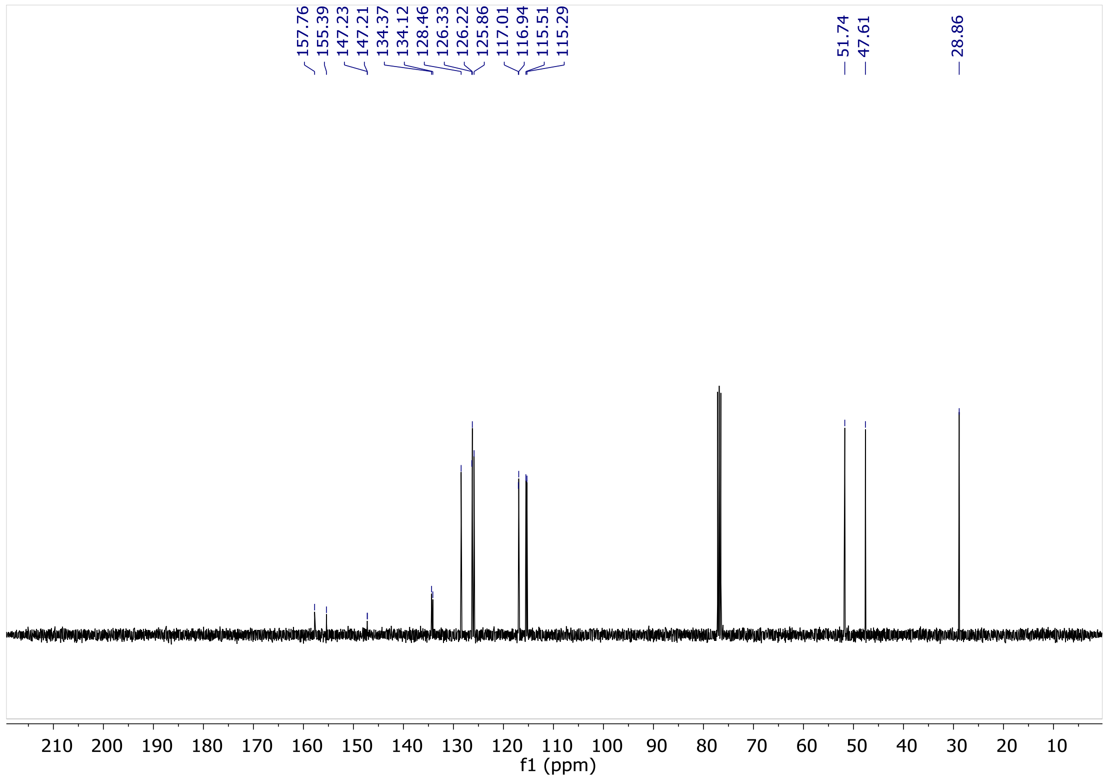

^{1}H NMR (400 MHz, $CDCl_3$). 2-(*p*-tolyl)-1,2,3,4-tetrahydroisoquinoline (**3e**)

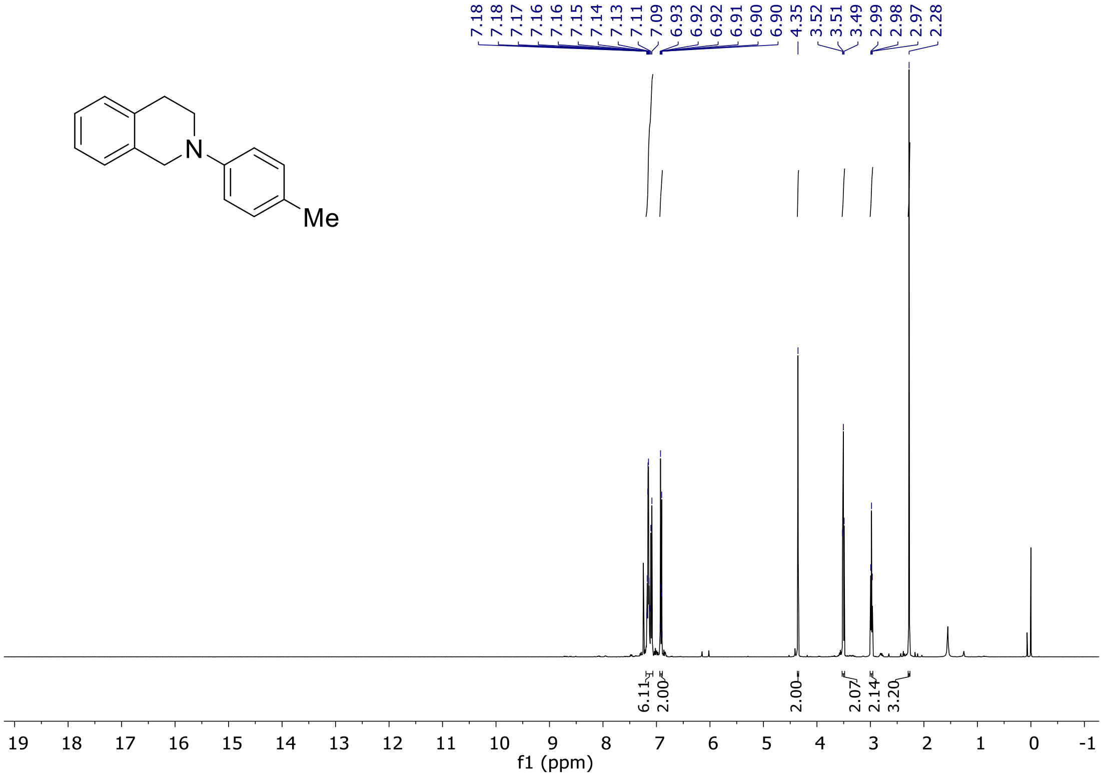

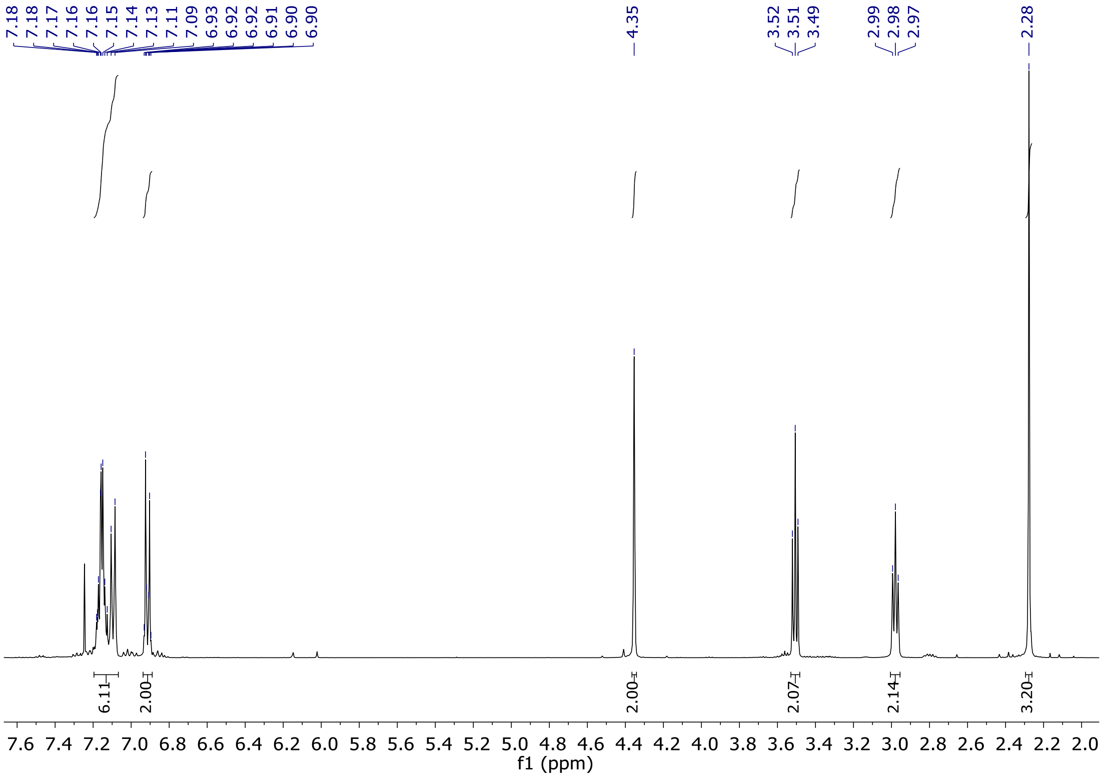

^{13}C NMR {^{1}H} (101 MHz, $CDCl_3$). 2-(p-tolyl)-1,2,3,4-tetrahydroisoquinoline (**3e**)

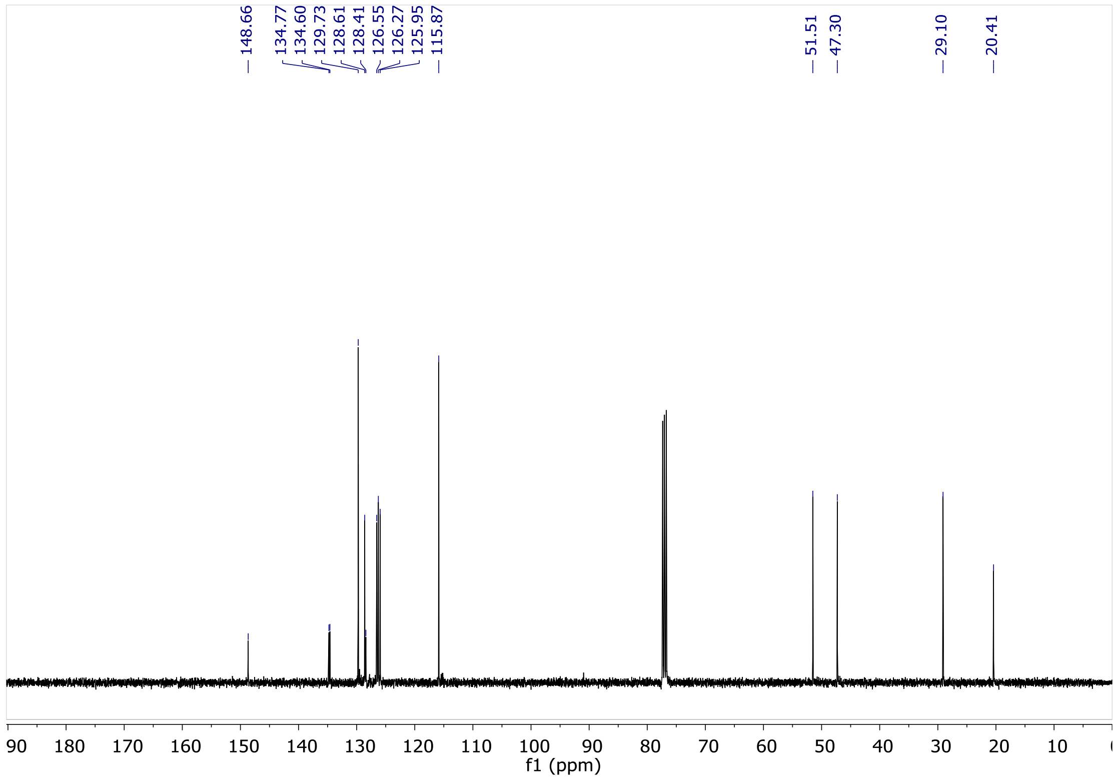

^{1}H NMR (400 MHz, $CDCl_3$). 1-(4-(3,4-dihydroisoquinolin-2(1H)-yl)phenyl)ethan-1-one (**3f**)

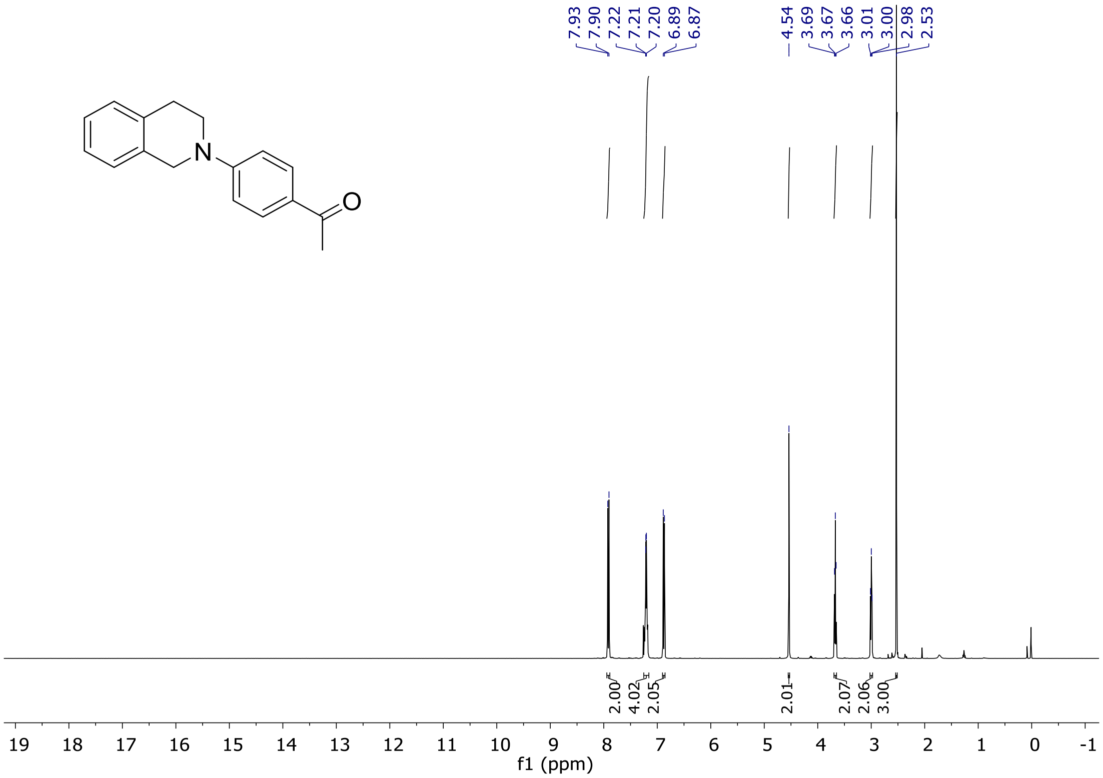

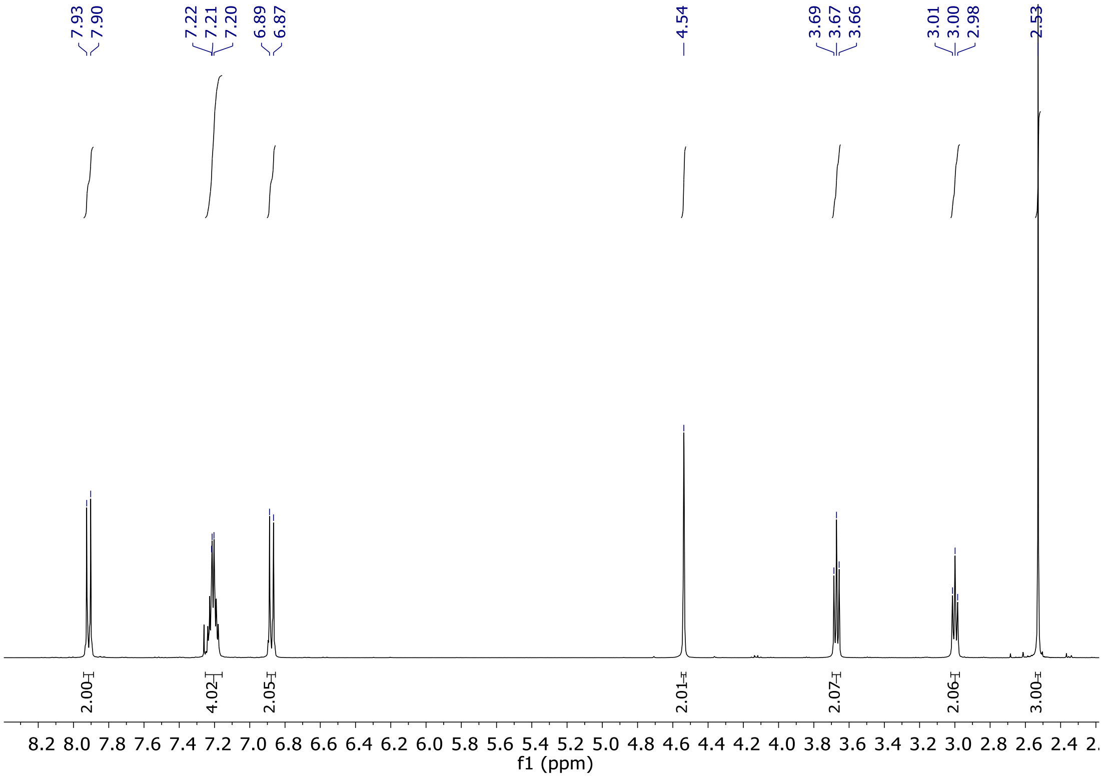

^{13}C NMR {^{1}H} (101 MHz, $CDCl_3$). 1-(4-(3,4-dihydroisoquinolin-2(1H)-yl)phenyl)ethan-1-one (**3f**)

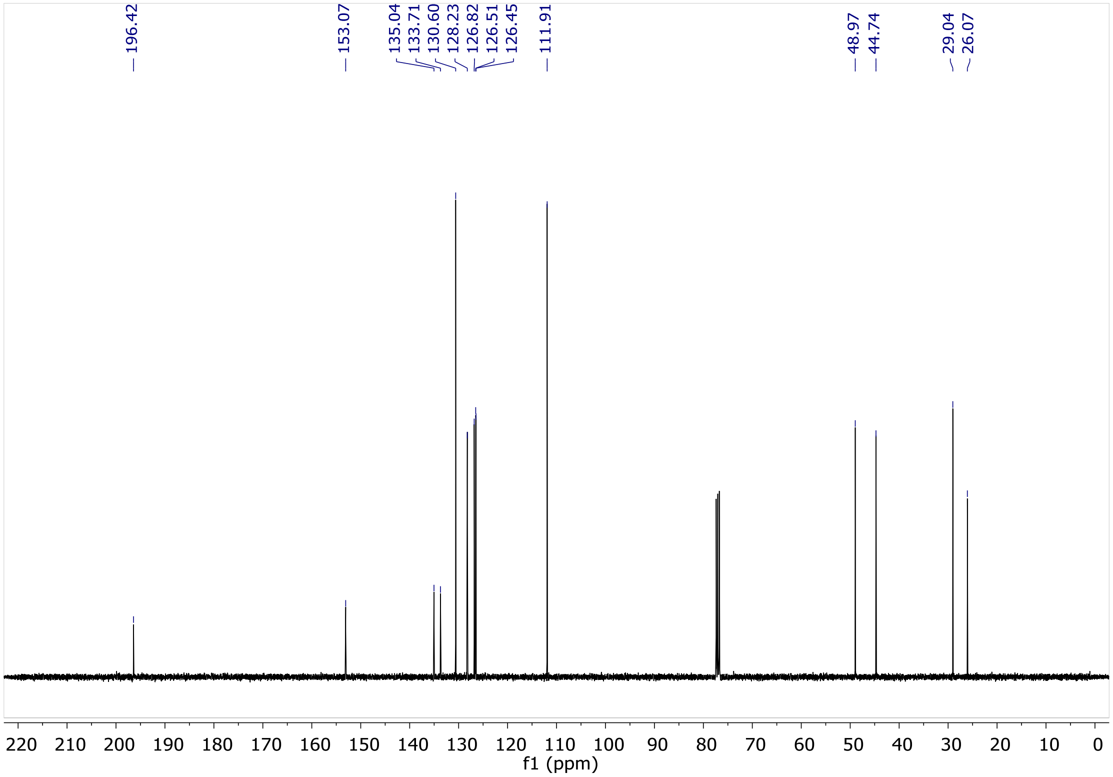

^{1}H NMR (400 MHz, $CDCl_3$). 2-(*o*-tolyl)-1,2,3,4-tetrahydroisoquinoline (**3g**)

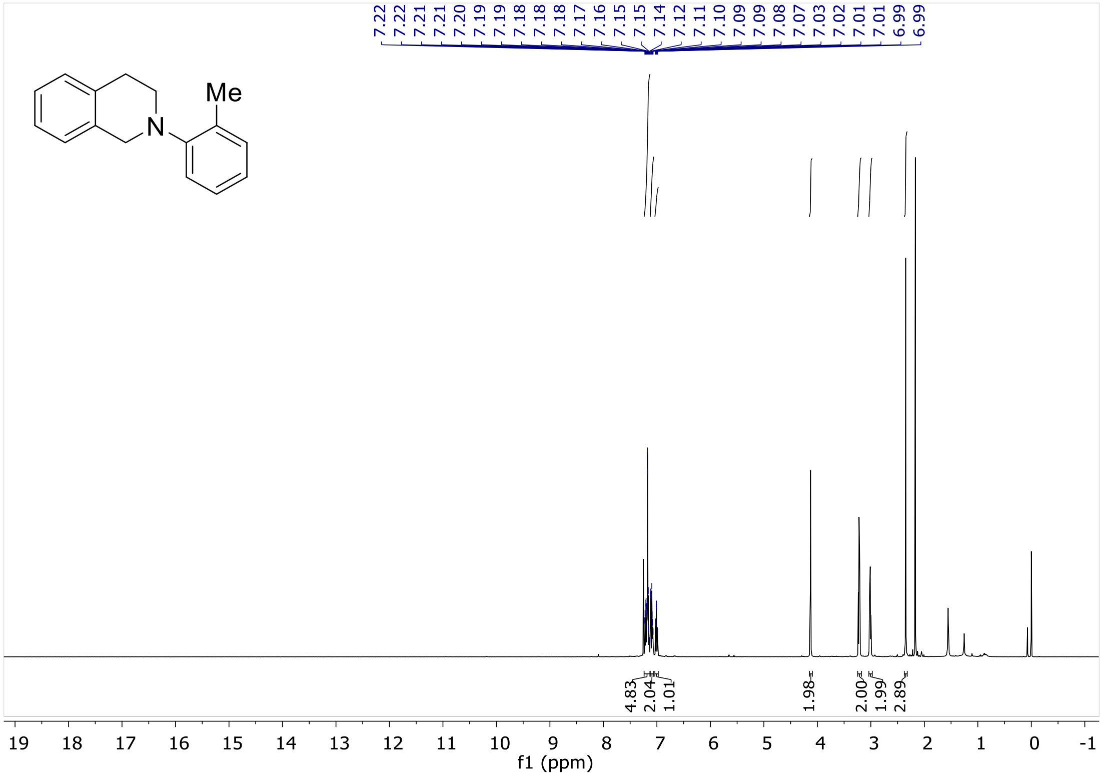

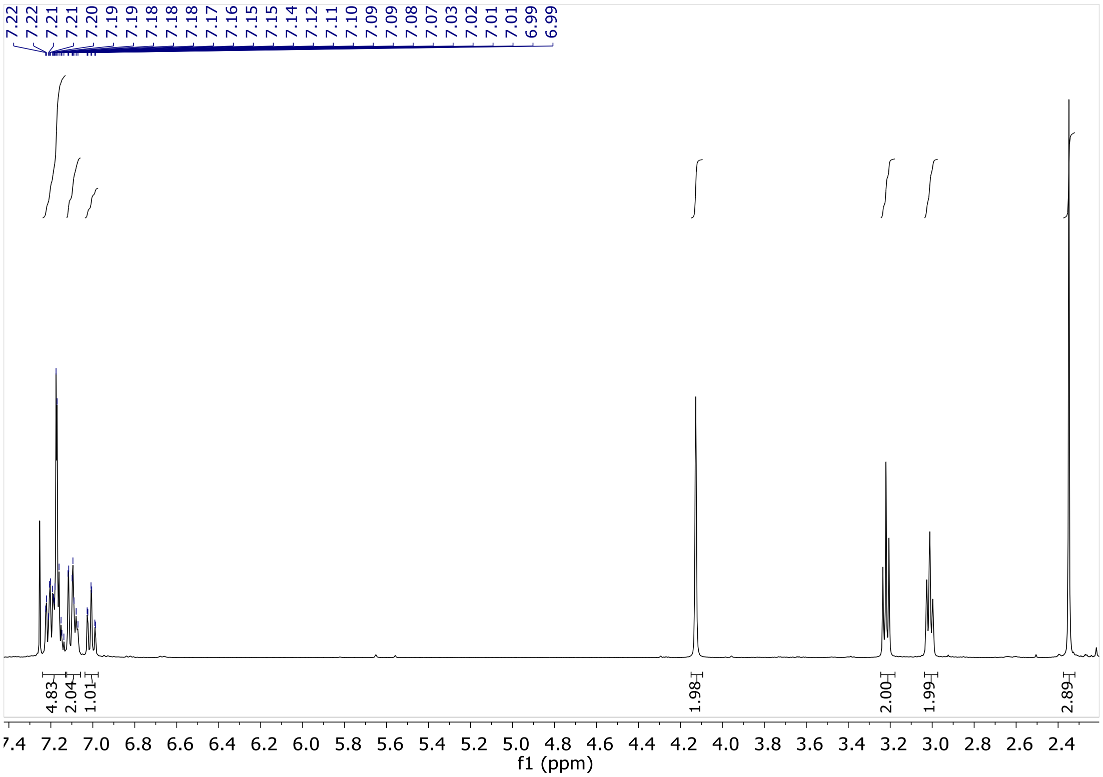

^{13}C NMR {^{1}H} (101 MHz, $CDCl_3$). 2-(*o*-tolyl)-1,2,3,4-tetrahydroisoquinoline (**3g**)

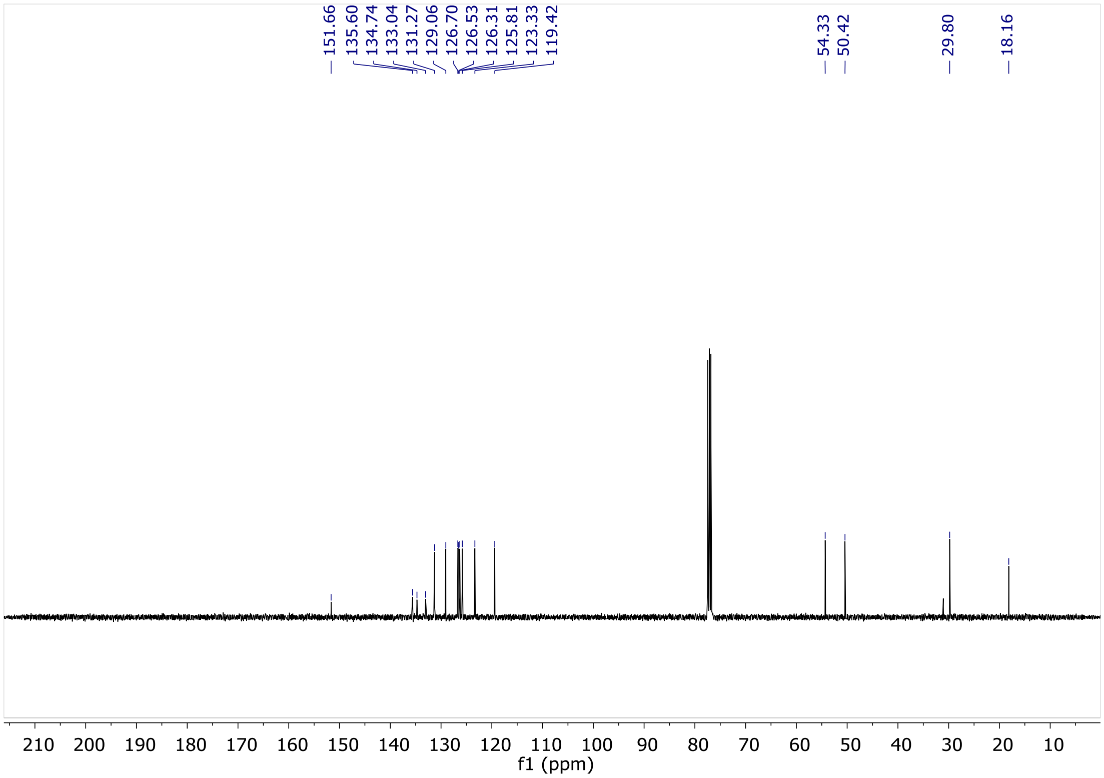

^{1}H NMR (400 MHz, $CDCl_3$). 1-(nitromethyl)-2-phenyl-1,2,3,4-tetrahydroisoquinoline (**4a**)

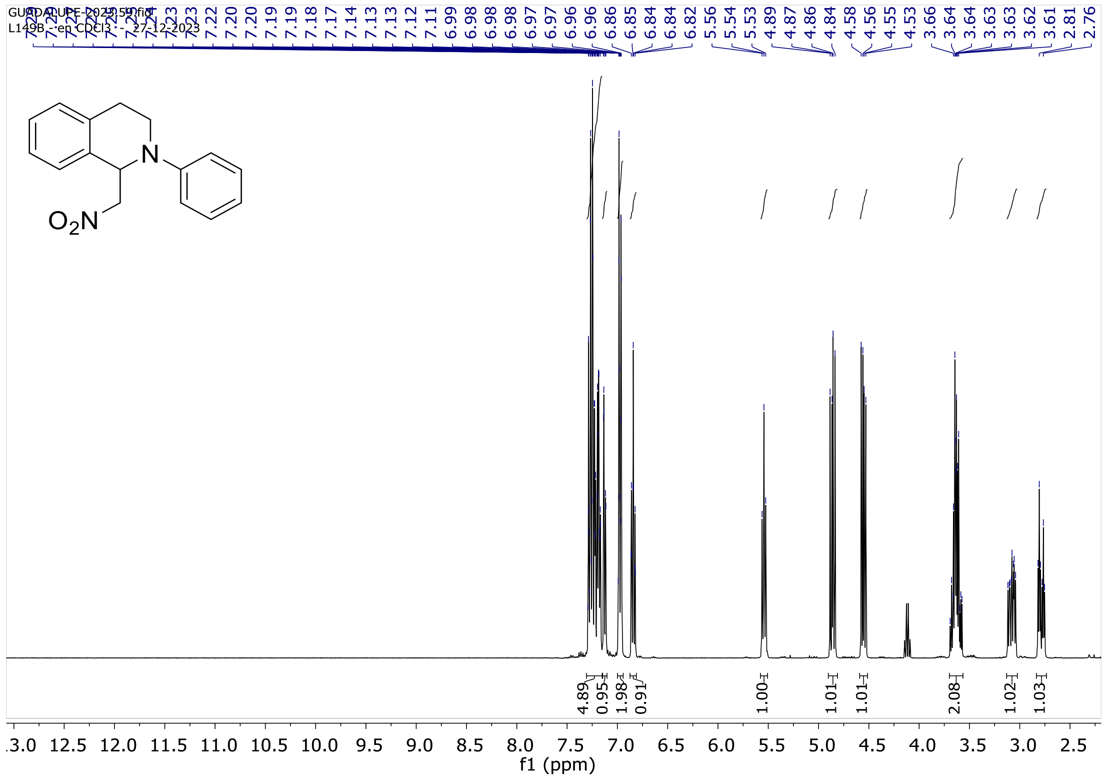

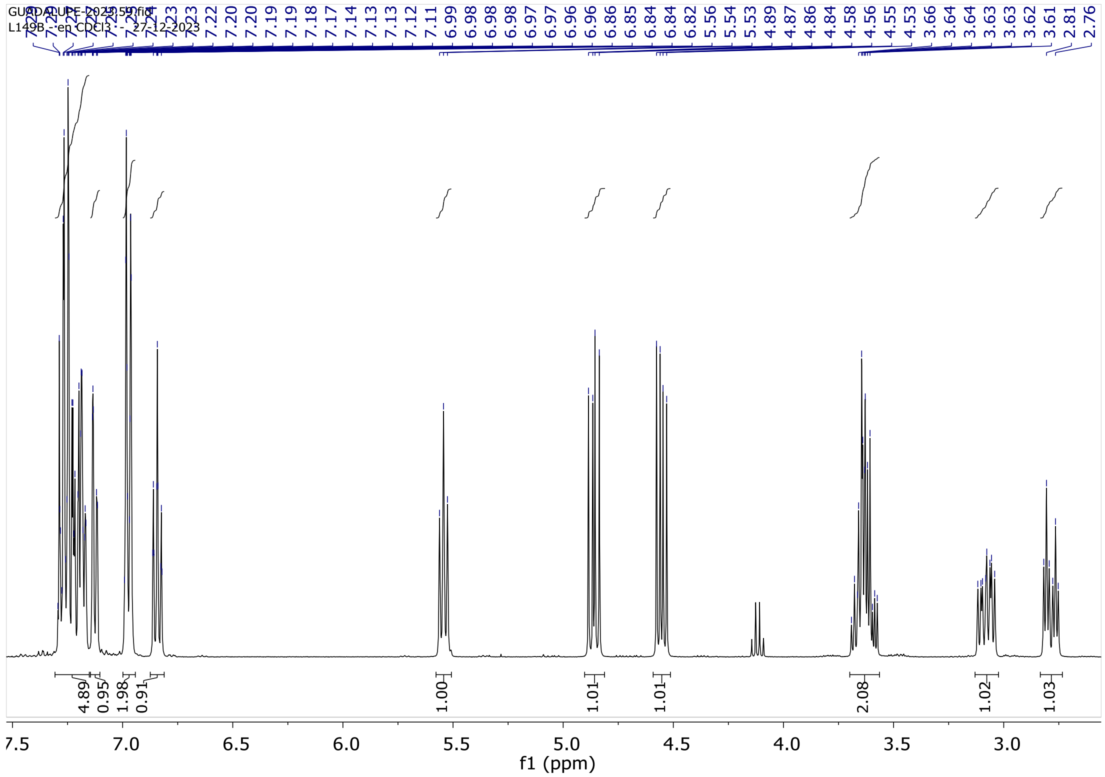

^{13}C NMR {^{1}H} (101 MHz, $CDCl_3$). 1-(nitromethyl)-2-phenyl-1,2,3,4-tetrahydroisoquinoline (**4a**)

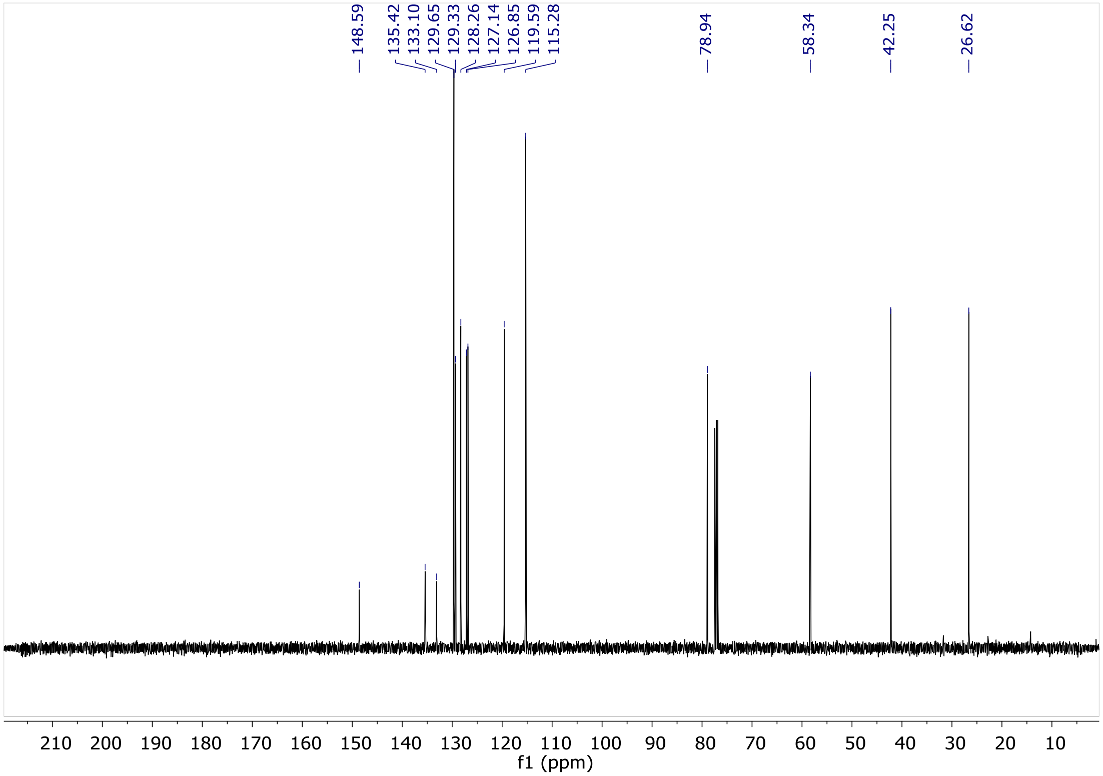

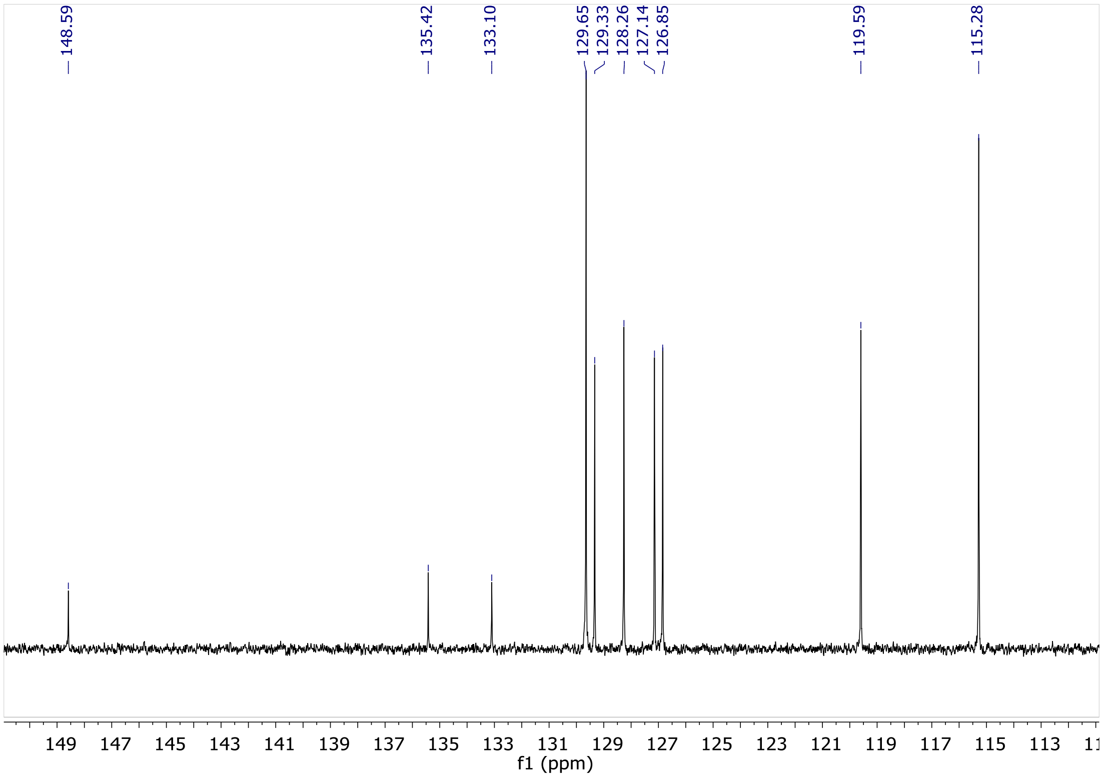

^{1}H NMR (400 MHz, $CDCl_3$). 2-(4-methoxyphenyl)-1-(nitromethyl)-1,2,3,4-tetrahydro isoquinoline (**4b**)

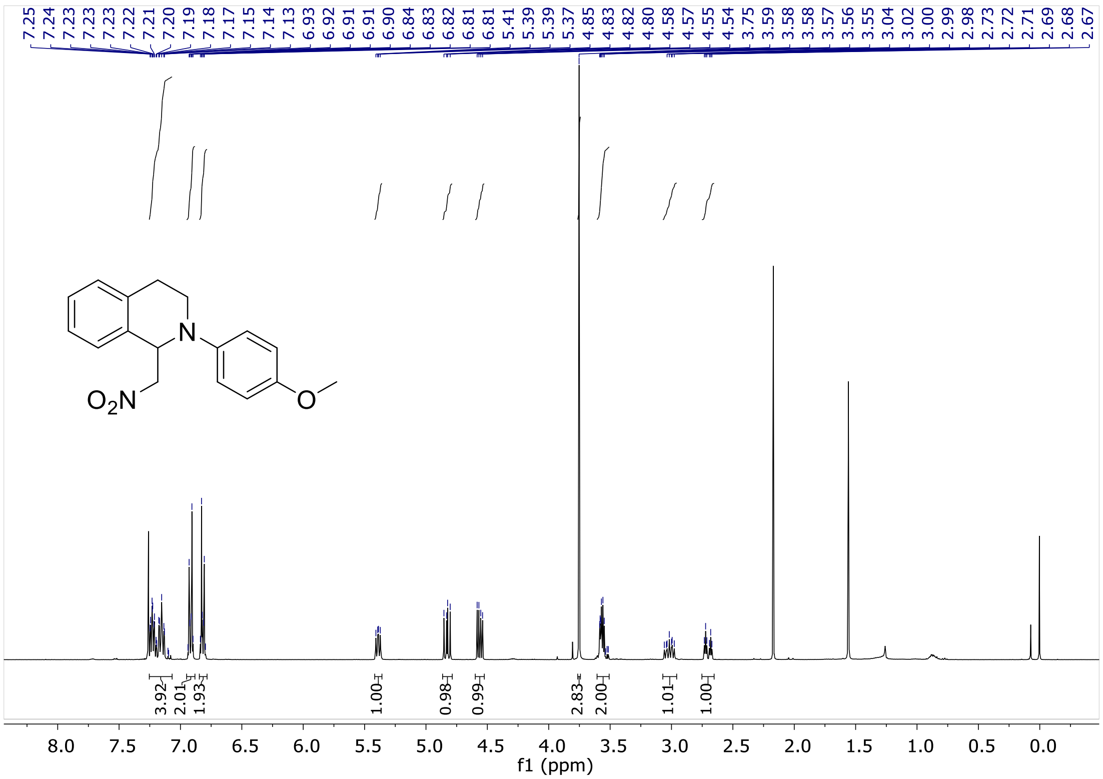

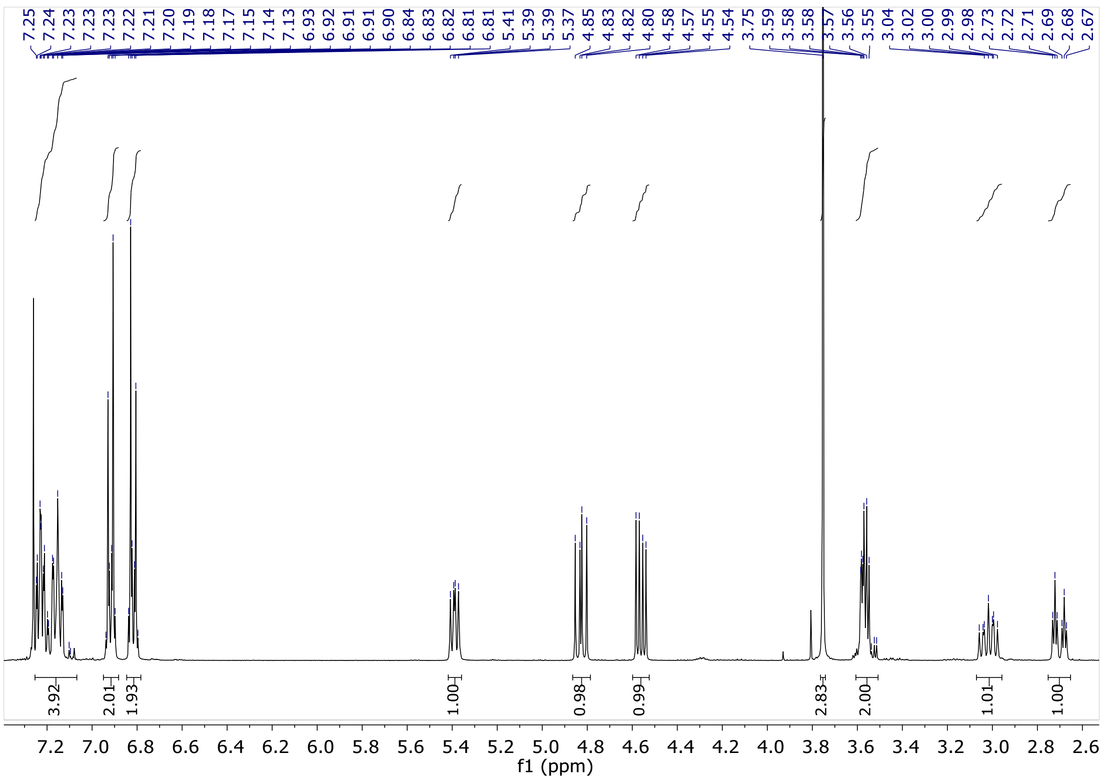

^{13}C NMR {^{1}H} (101 MHz, $CDCl_3$). 2-(4-methoxyphenyl)-1-(nitromethyl)-1,2,3,4-tetrahydro isoquinoline (**4b**)

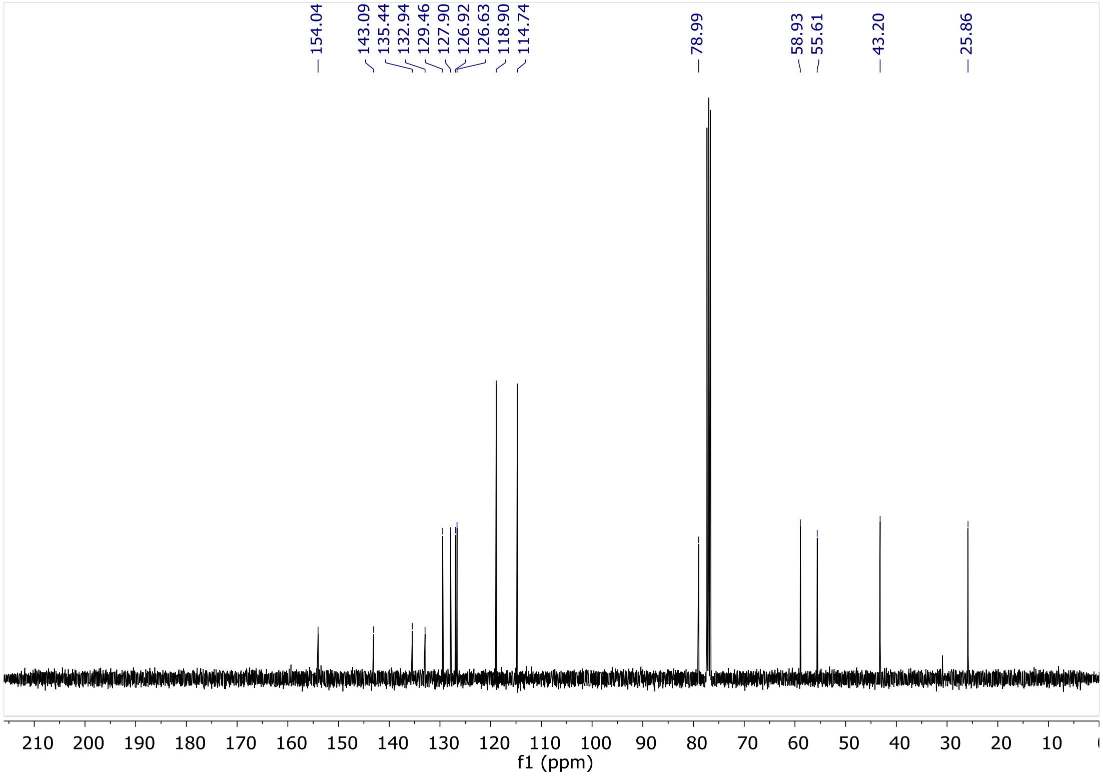

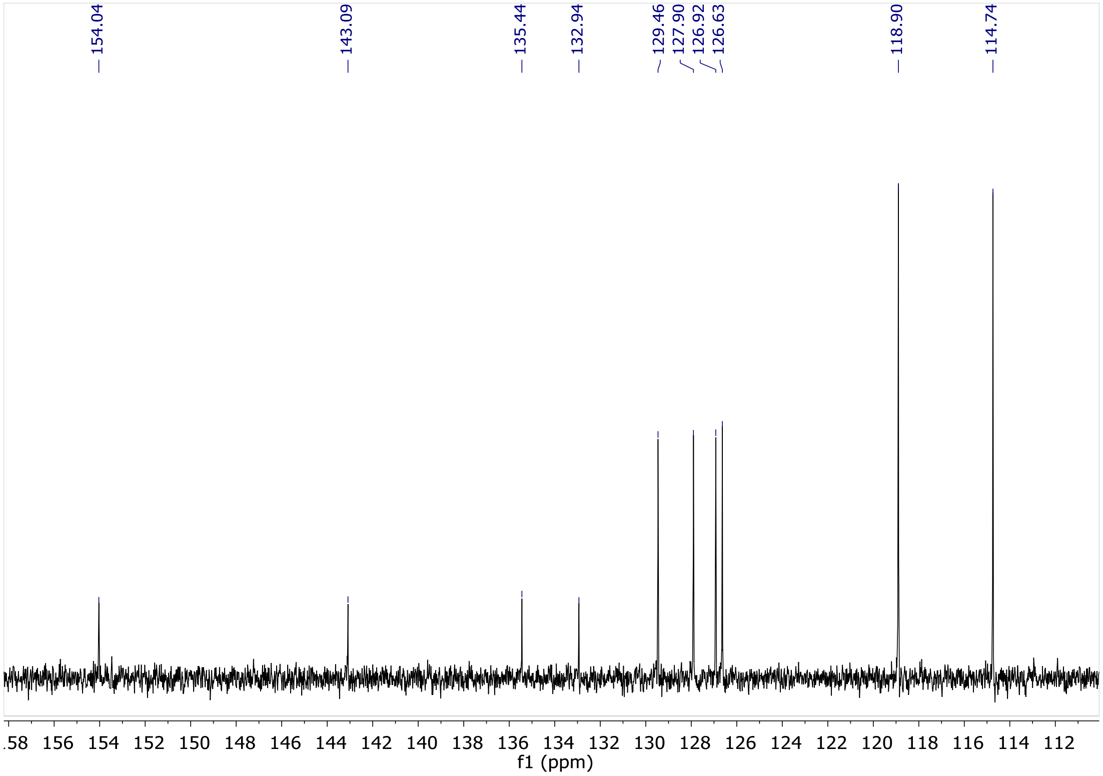

^{1}H NMR (400 MHz, $CDCl_3$). 1-(nitromethyl)-2-(4-(trifluoromethyl)phenyl)-1,2,3,4-tetrahydroisoquinoline (**4c**)

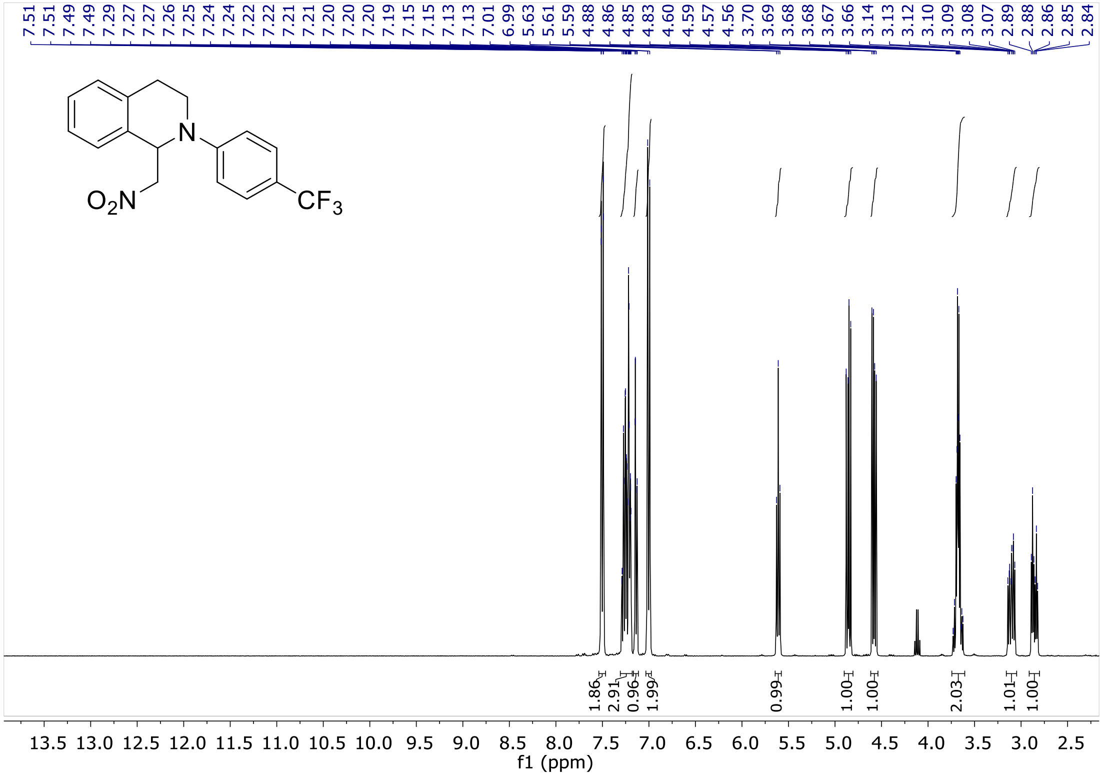

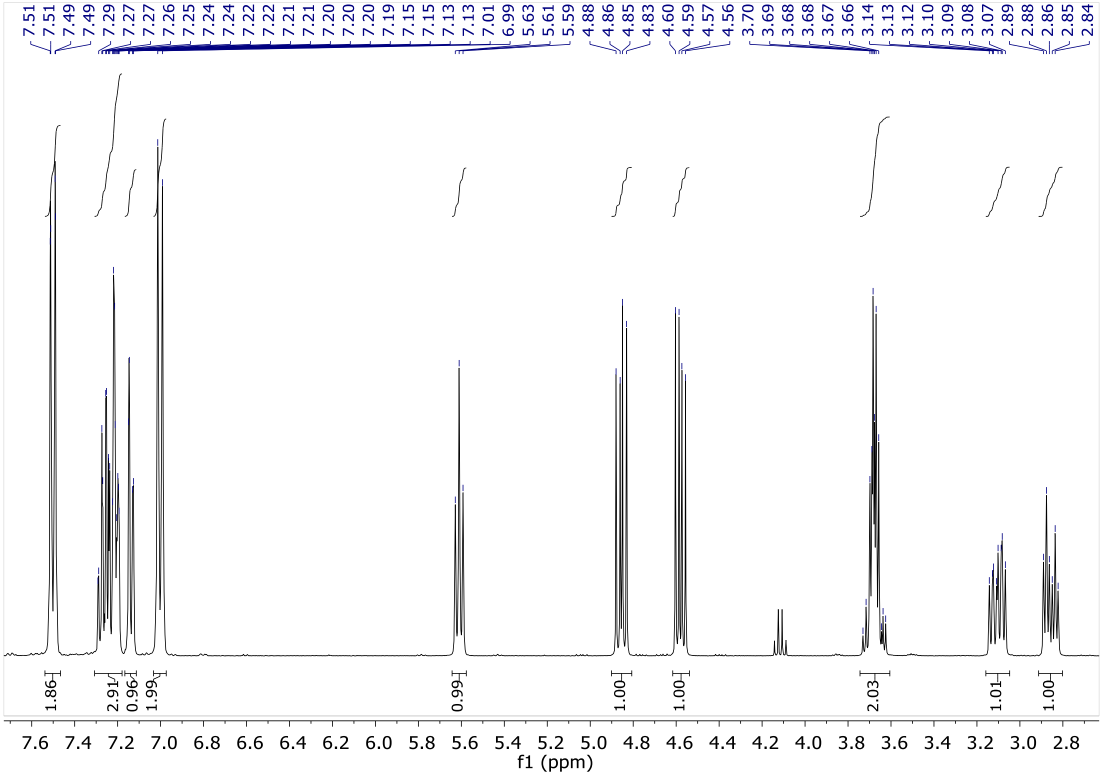

^{13}C NMR {^{1}H} (101 MHz, $CDCl_3$). 1-(nitromethyl)-2-(4-(trifluoromethyl)phenyl)-1,2,3,4-tetrahydroisoquinoline (**4c**)

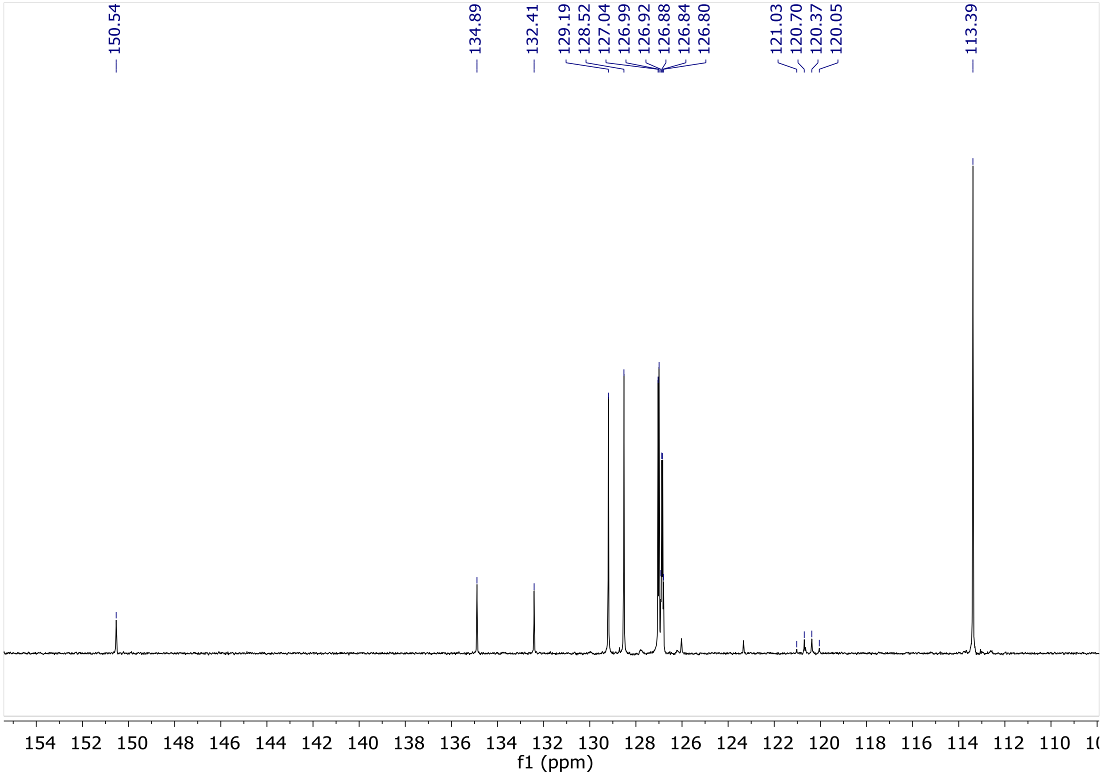

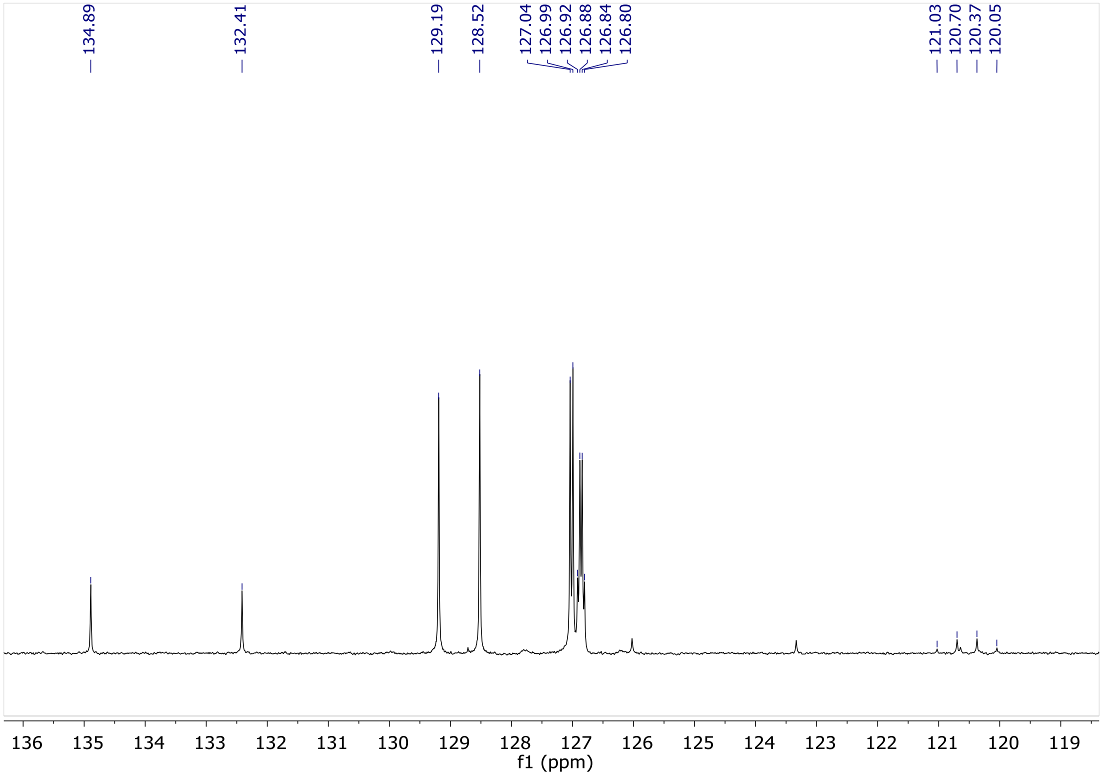

^{19}F NMR (376 MHz, $CDCl_3$). 1-(nitromethyl)-2-(4-(trifluoromethyl)phenyl)-1,2,3,4-tetrahydroisoquinoline (**4c**)

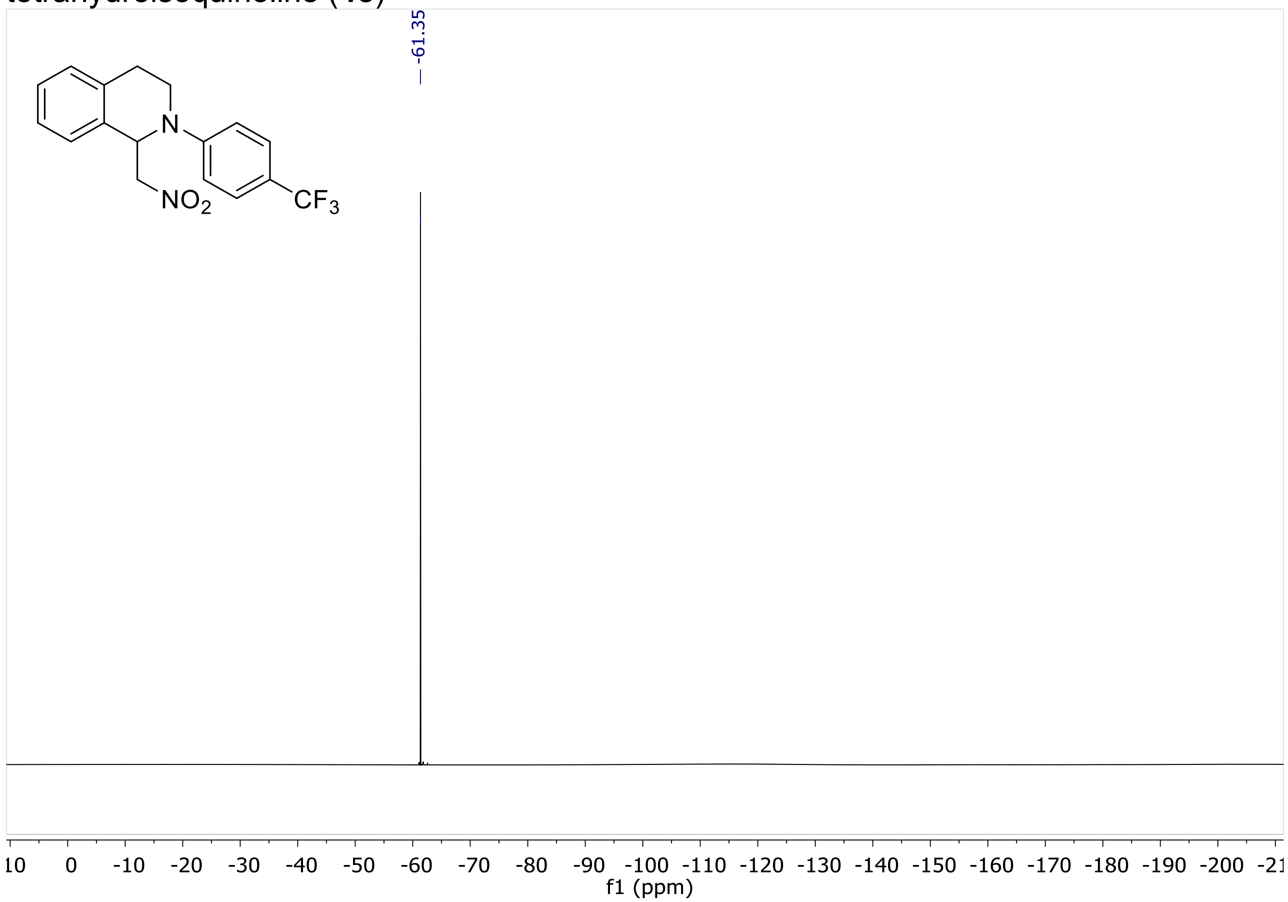

^{1}H NMR (400 MHz, $CDCl_3$). 2-(4-fluorophenyl)-1-(nitromethyl)-1,2,3,4-tetrahydroisoquinoline (**4d**)

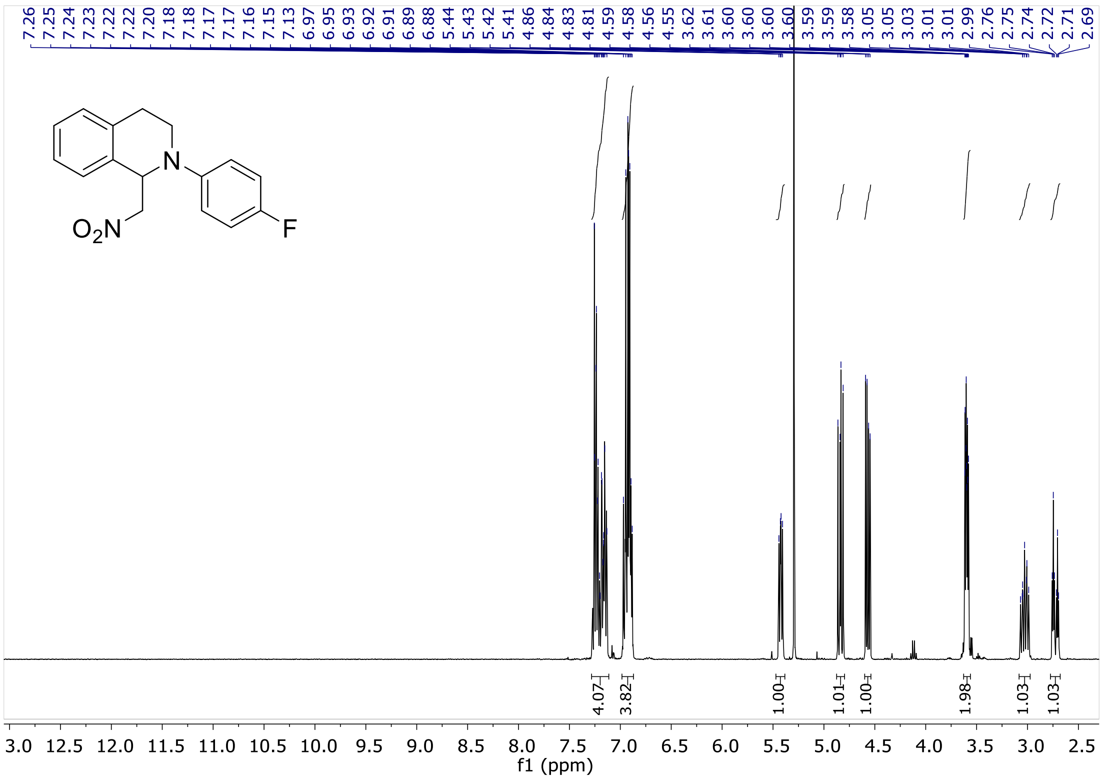

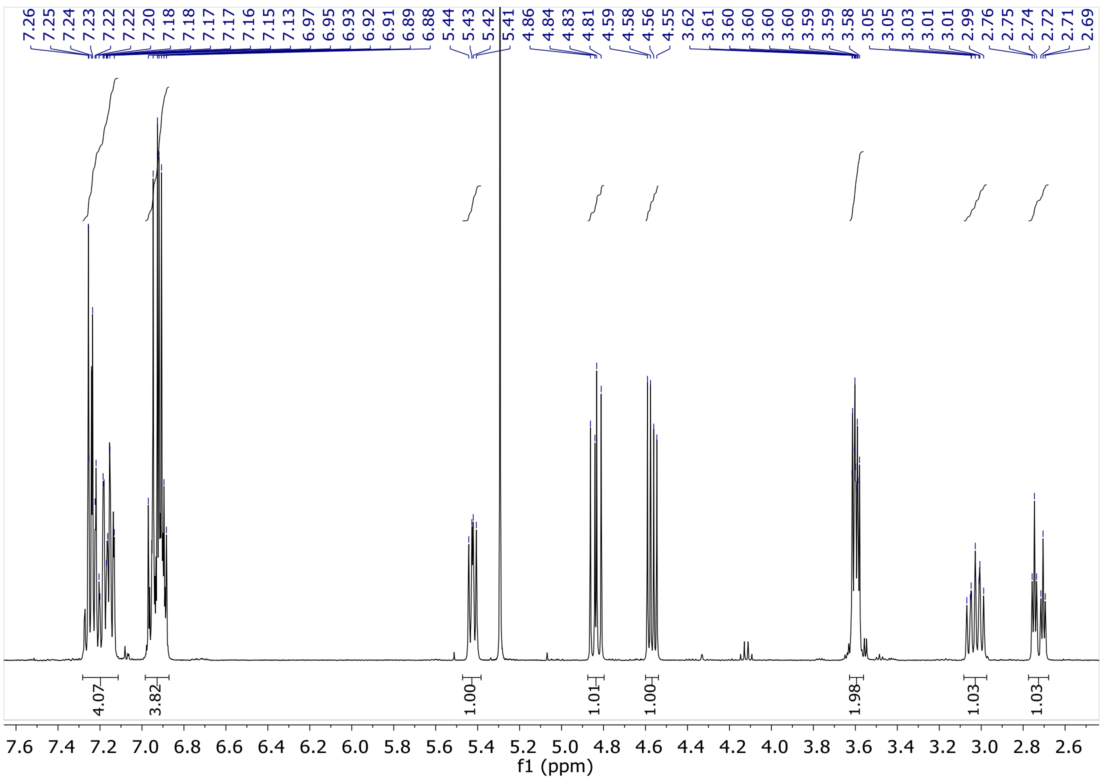

^{13}C NMR {^{1}H} (101 MHz, $CDCl_3$). 2-(4-fluorophenyl)-1-(nitromethyl)-1,2,3,4-tetrahydro isoquinoline (**4d**)

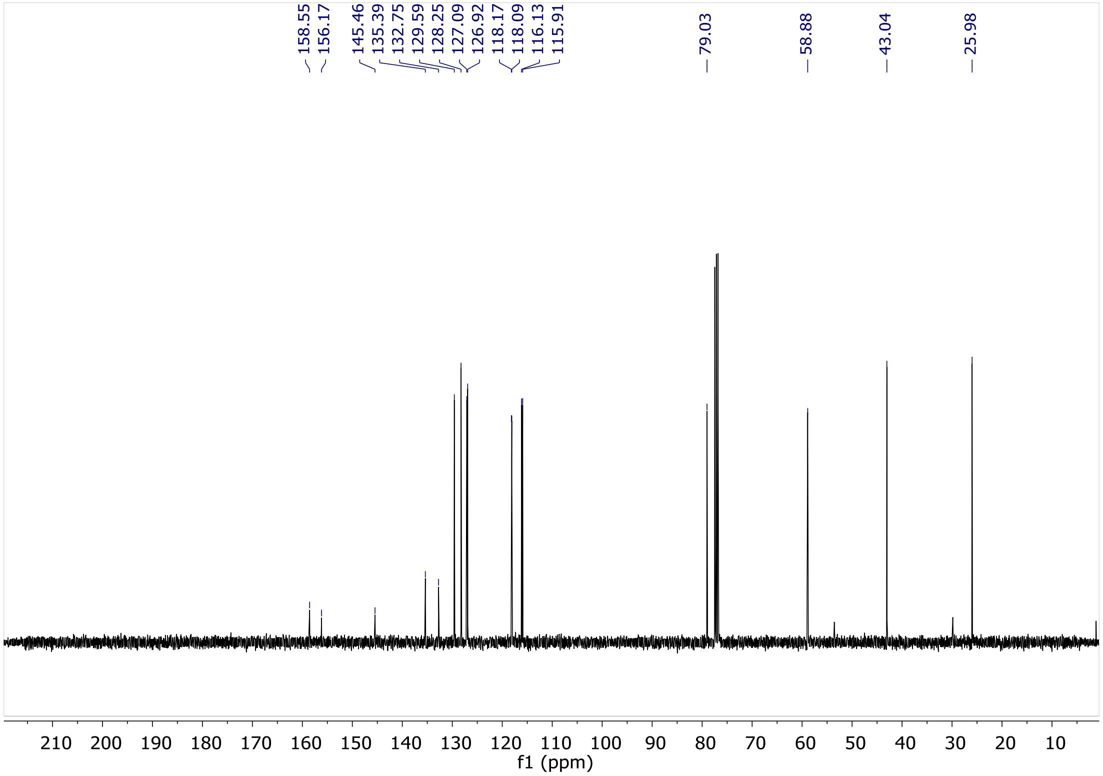

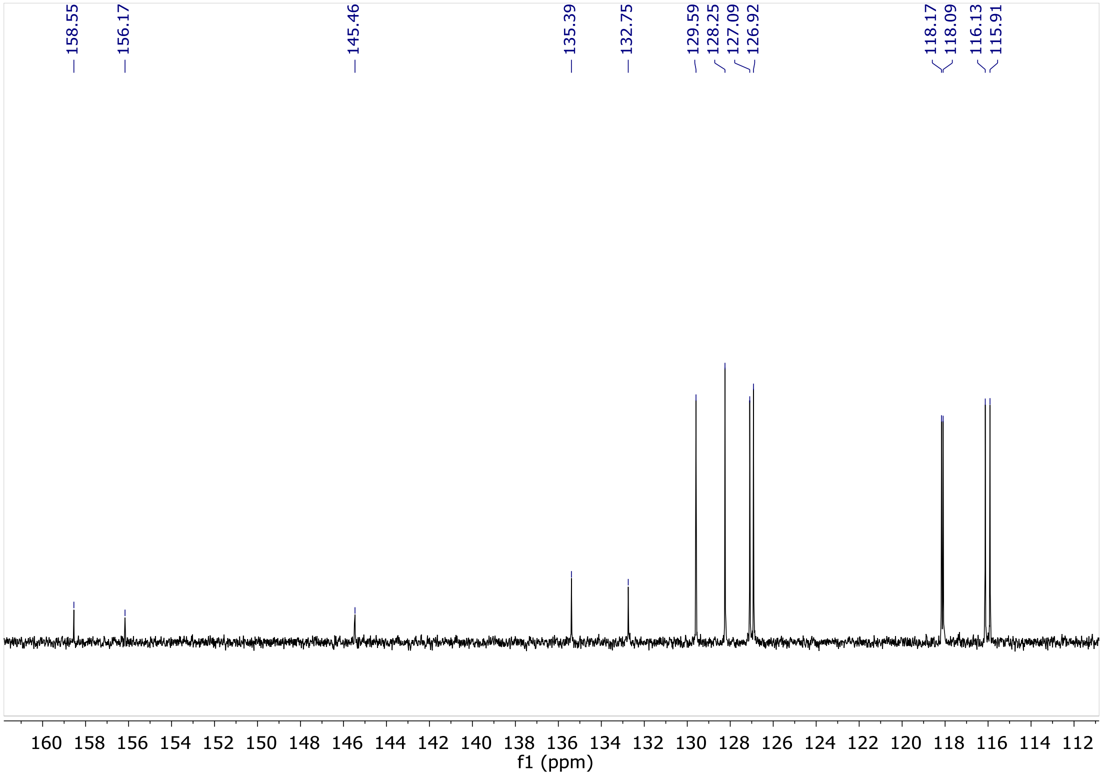

^{19}F NMR (376 MHz, $CDCl_3$). 2-(4-fluorophenyl)-1-(nitromethyl)-1,2,3,4-tetrahydroisoquinoline (**4d**)

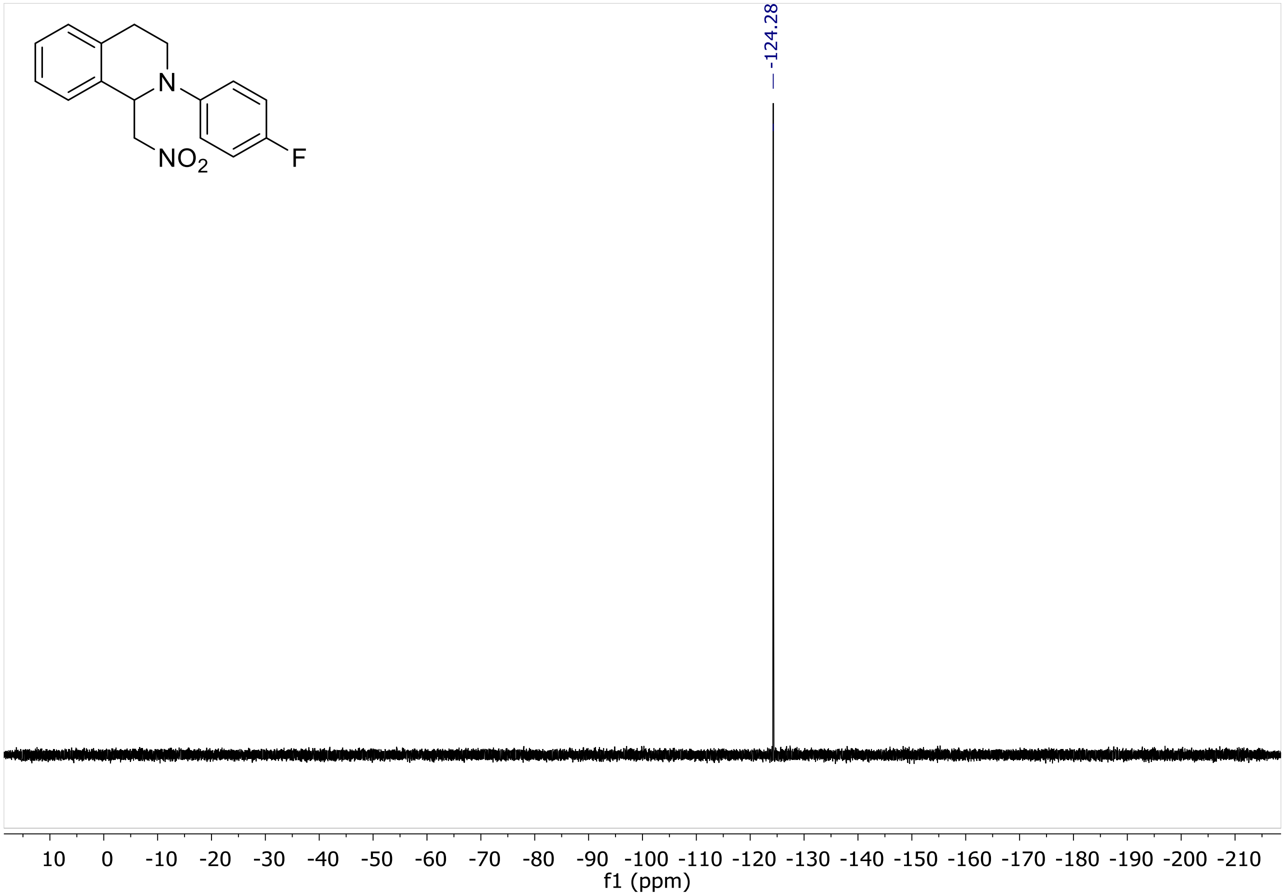

¹H NMR (400 MHz, $CDCl_3$). 1-(nitromethyl)-2-(*p*-tolyl)-1,2,3,4-tetrahydroisoquinoline (**4e**)

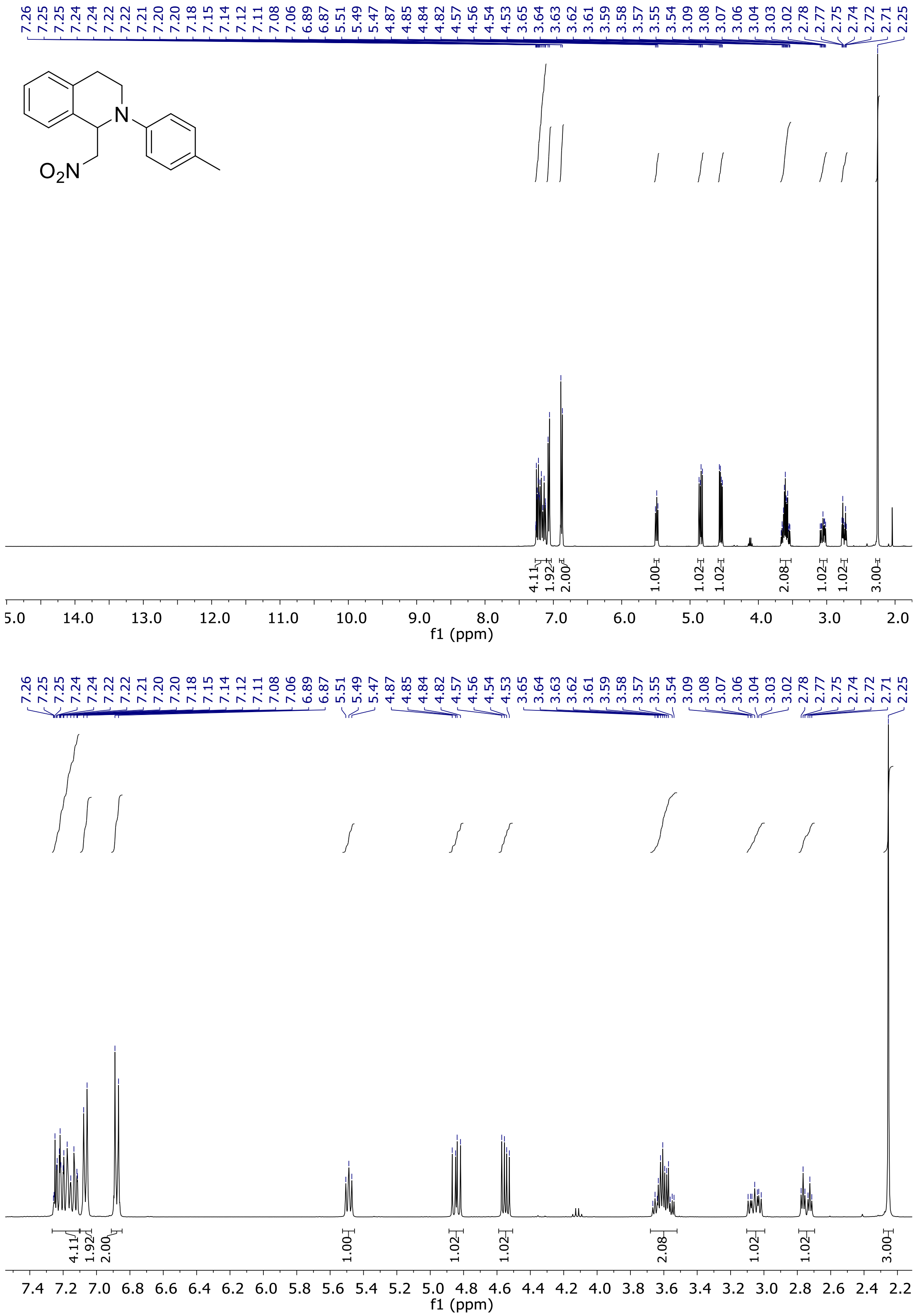

^{13}C NMR {^{1}H} (101 MHz, $CDCl_3$). 1-(nitromethyl)-2-(*p*-tolyl)-1,2,3,4-tetrahydroisoquinoline (**4e**)

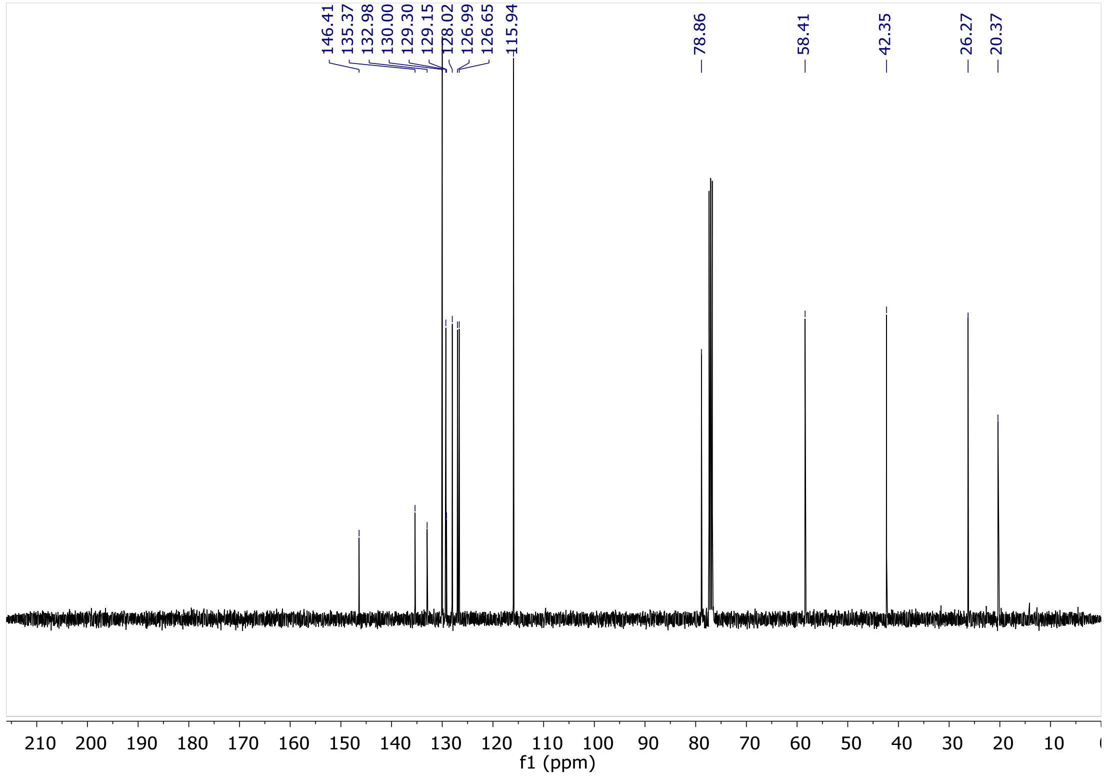

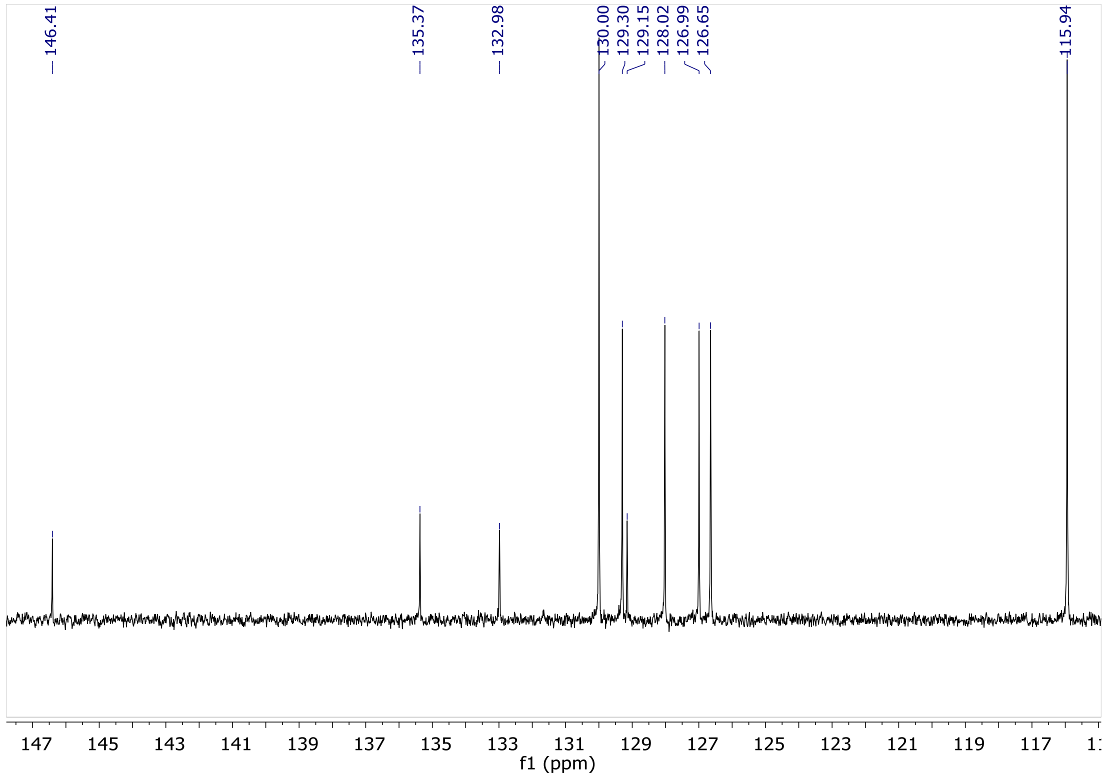

^{1}H NMR (400 MHz, $CDCl_3$). 1-(4-(1-(nitromethyl)-3,4-dihydroisoquinolin-2(1H)-yl) phenyl) ethan-1-one (**4f**)

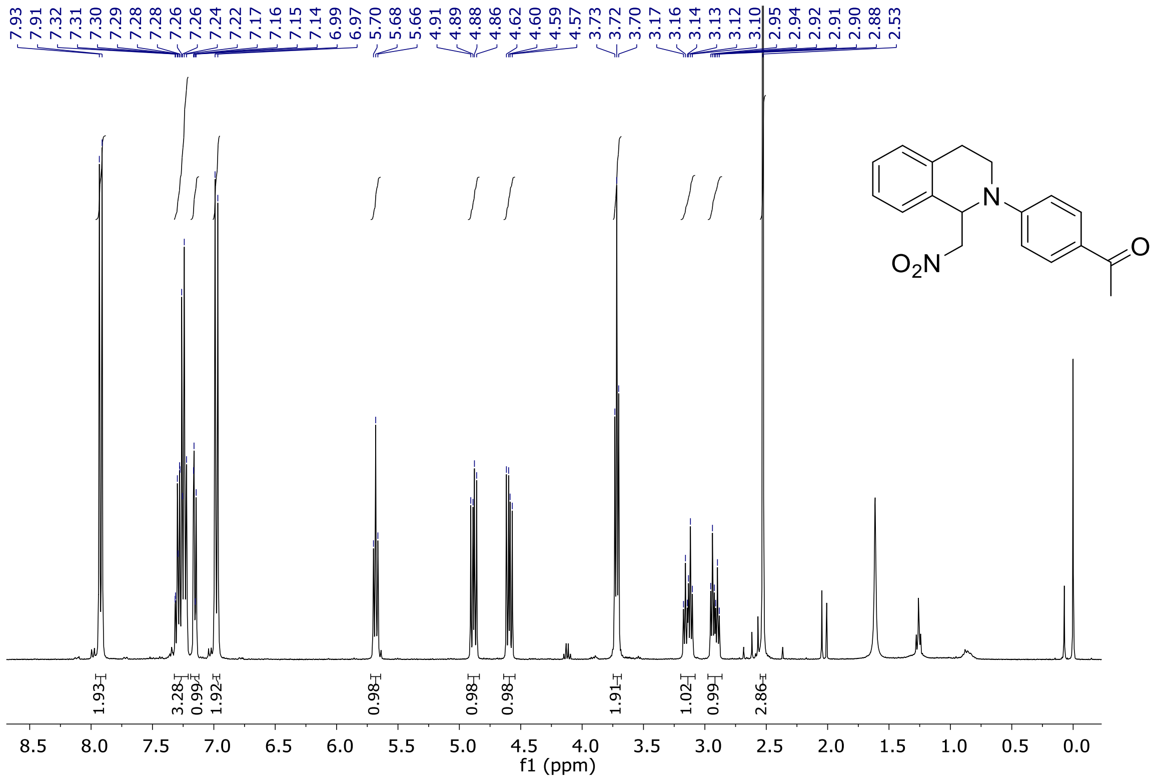

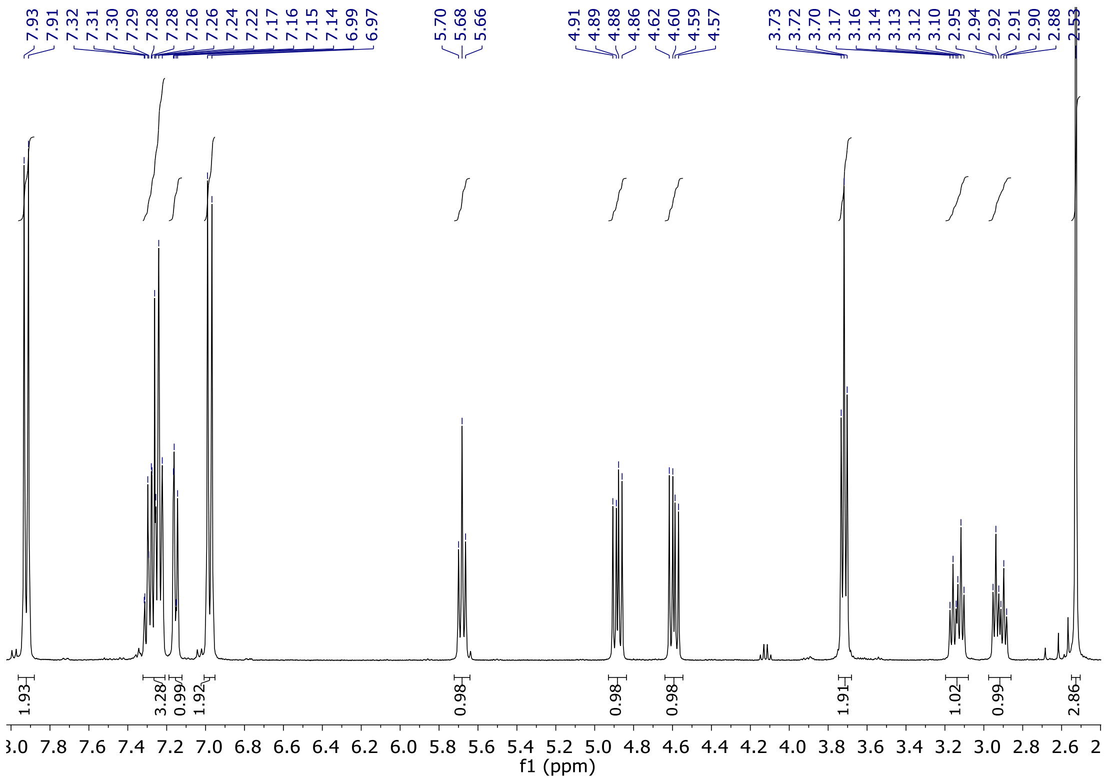

^{13}C NMR {^{1}H} (101 MHz, $CDCl_3$). 1-(4-(1-(nitromethyl)-3,4-dihydroisoquinolin-2(1H)-yl) phenyl) ethan-1-one (**4f**)

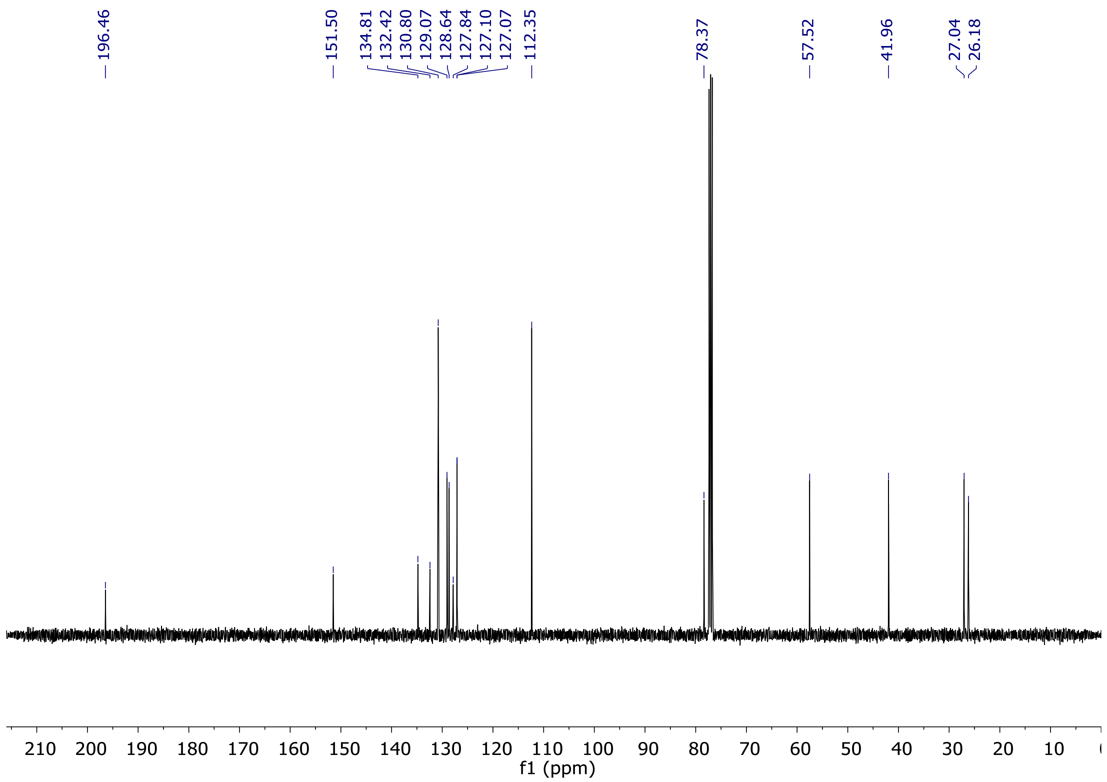

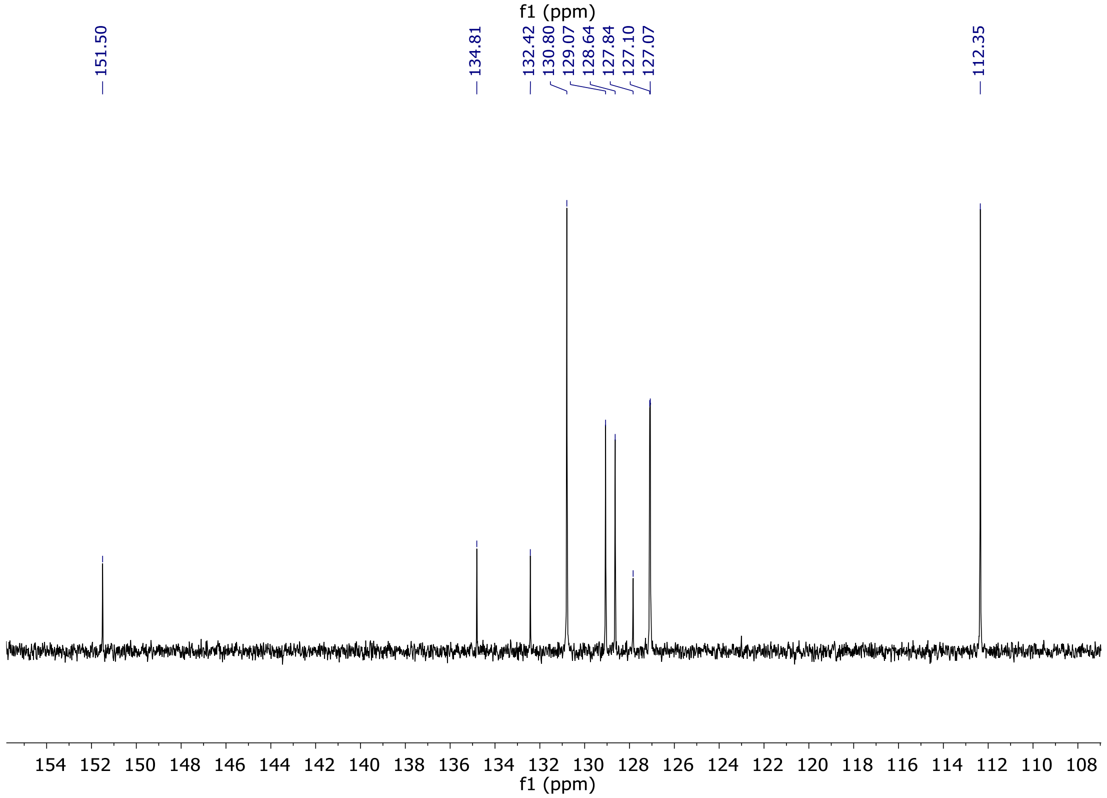

^{1}H NMR (400 MHz, $CDCl_3$). 1-(nitromethyl)-2-(*o*-tolyl)-1,2,3,4-tetrahydroisoquinoline (**4g**)

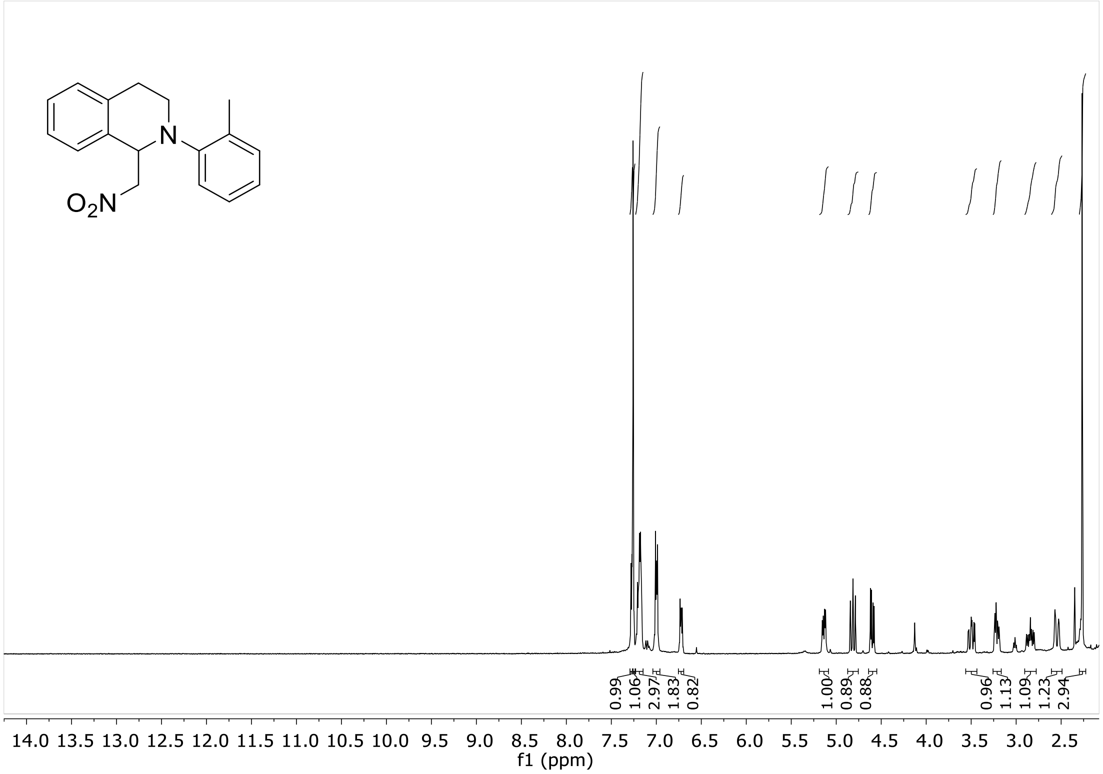

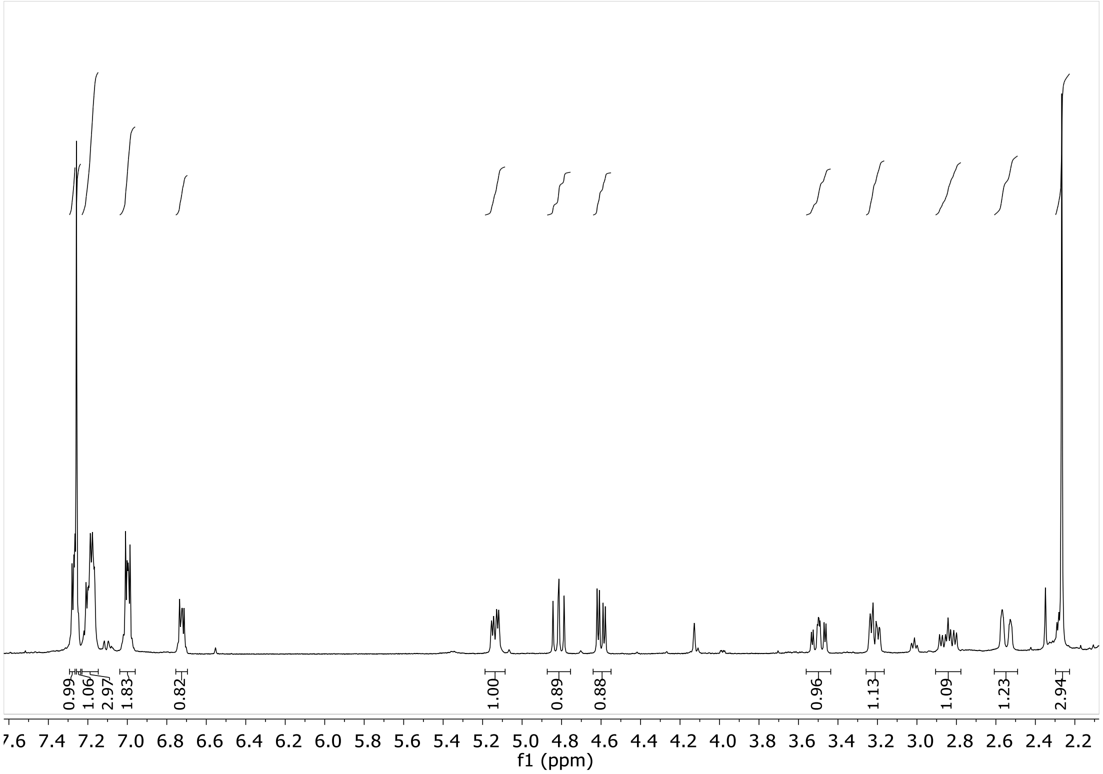

^{13}C NMR {^{1}H} (101 MHz, $CDCl_3$). 1-(nitromethyl)-2-(*o*-tolyl)-1,2,3,4-tetrahydroisoquinoline (**4g**)

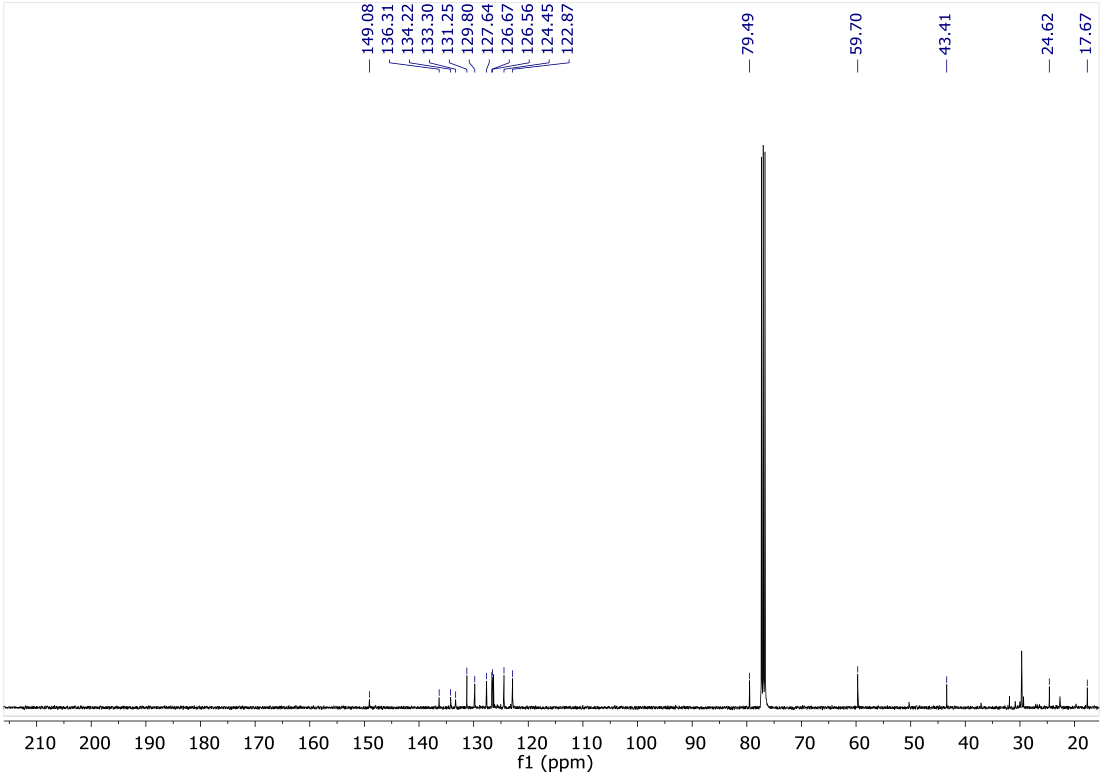

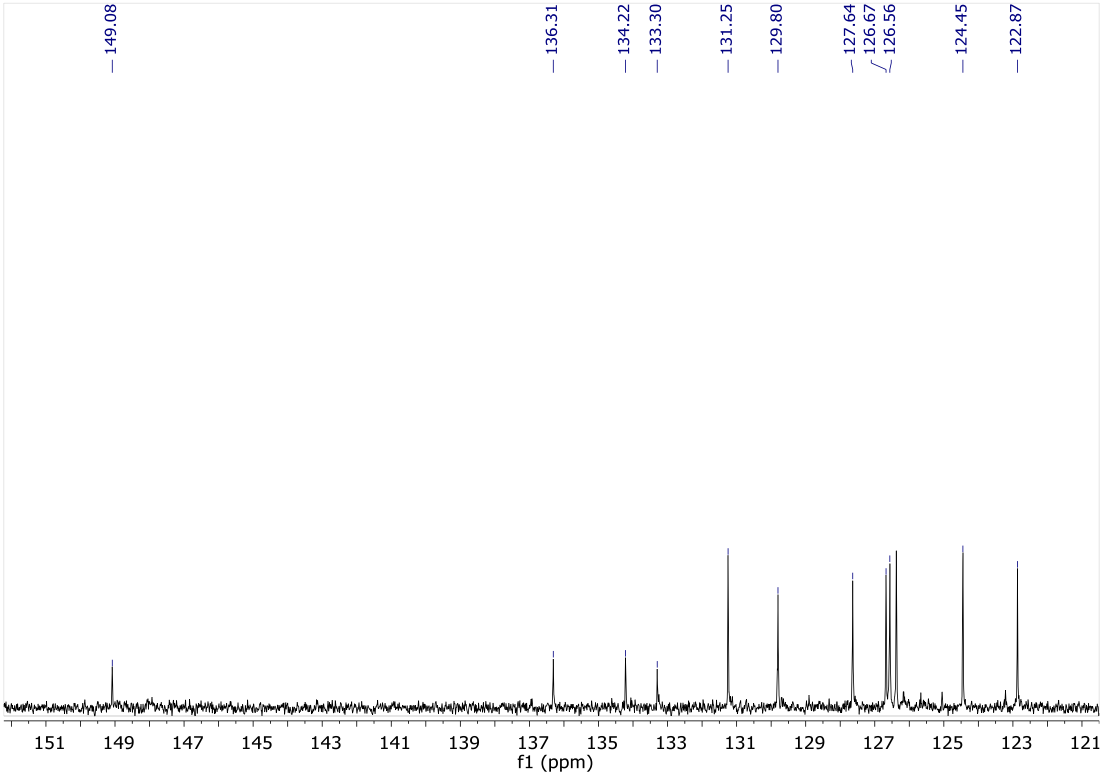

^{1}H NMR (400 MHz, $CDCl_3$). 1-(1-nitropropyl)-2-phenyl-1,2,3,4-tetrahydroisoquinoline (**4h**)

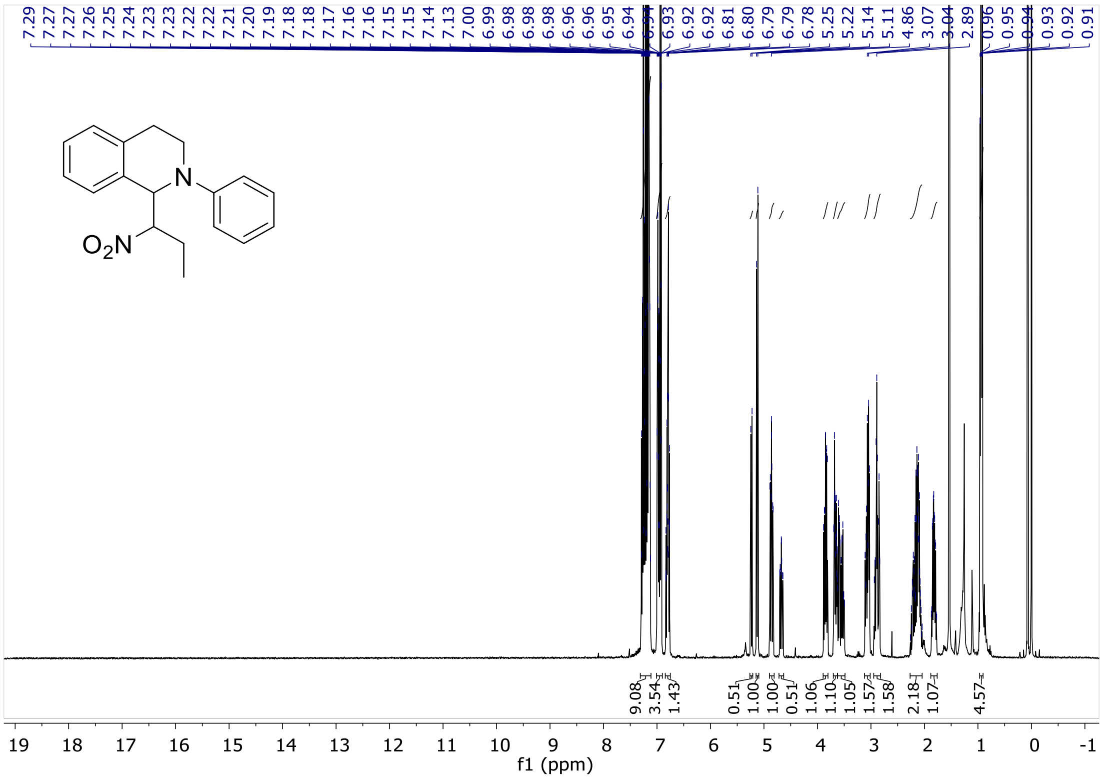

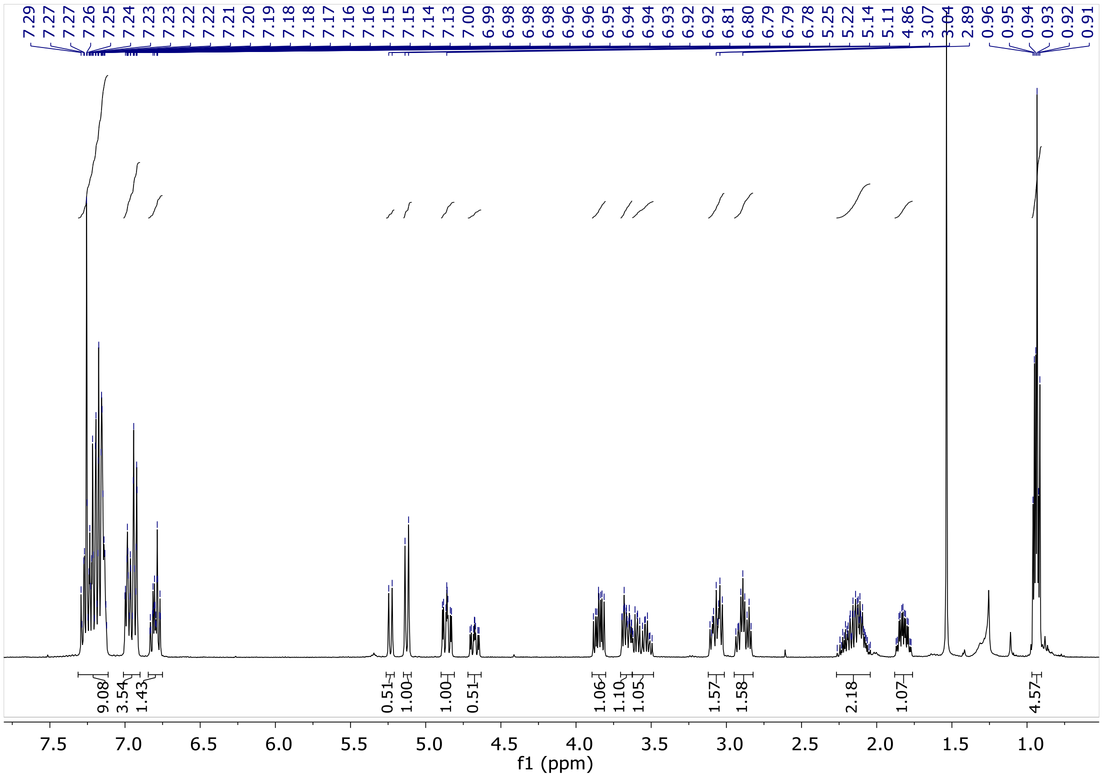

^{13}C NMR {^{1}H} (101 MHz, $CDCl_3$). 1-(1-nitropropyl)-2-phenyl-1,2,3,4-tetrahydroisoquinoline (**4h**)

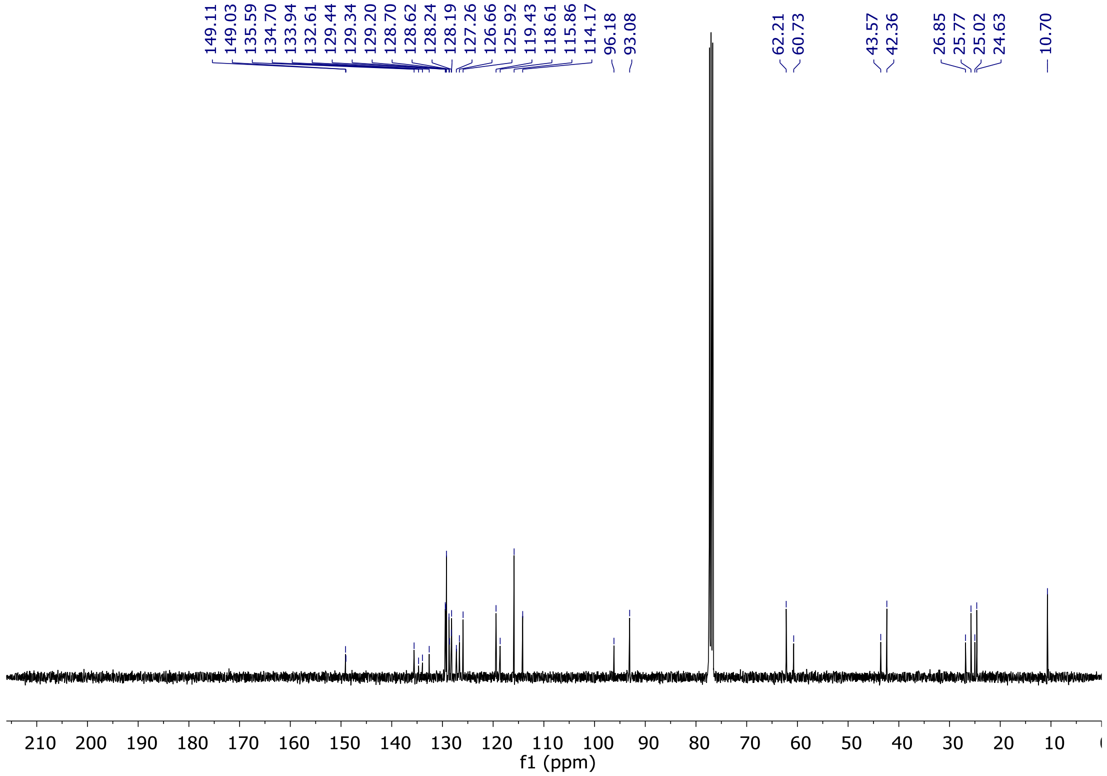

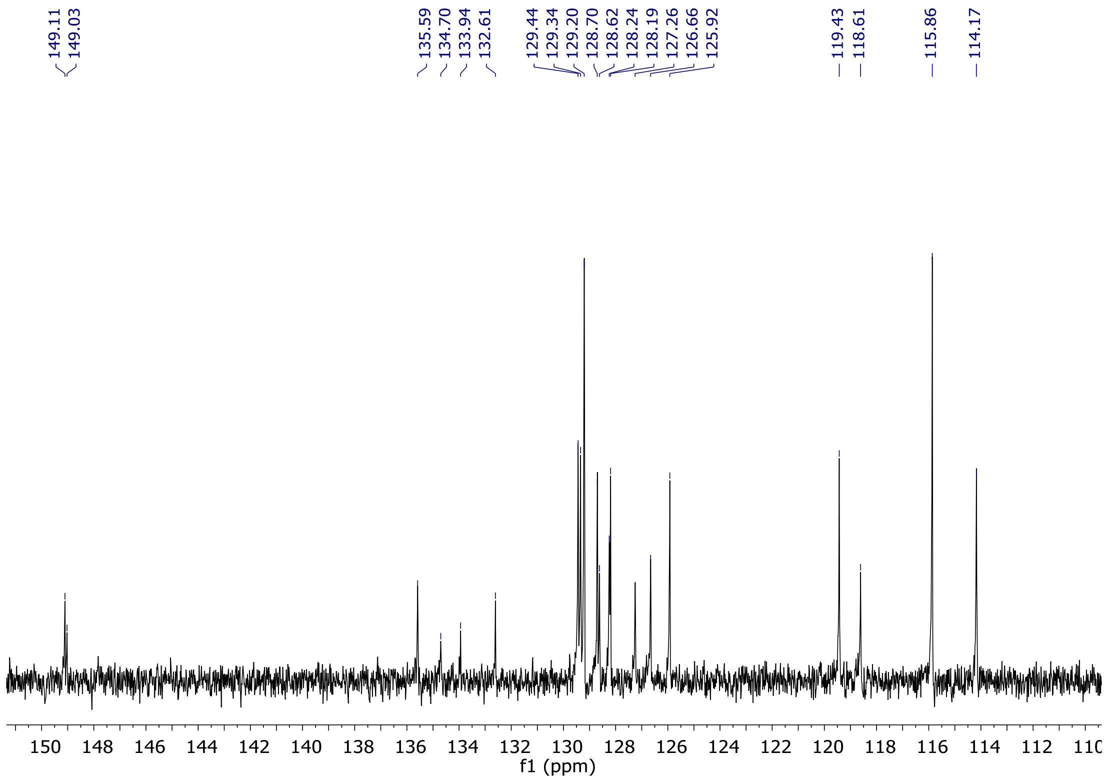

^{1}H NMR (400 MHz, $CDCl_3$). 9,10-dimethyl-9,10-dihydro-9,10-epidioxyanthracene (**DMA-O$_2$**)

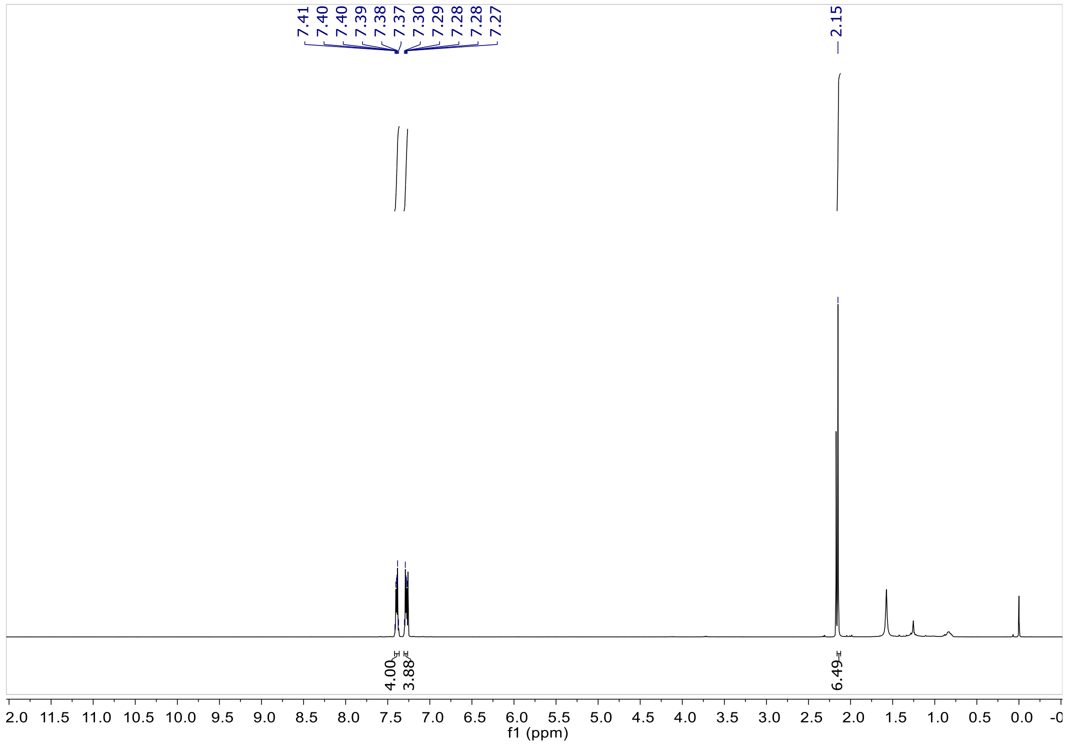

^{13}C NMR {^{1}H} (101 MHz, $CDCl_3$). 9,10-dimethyl-9,10-dihydro-9,10-epidioxyanthracene (**DMA-O$_2$**)

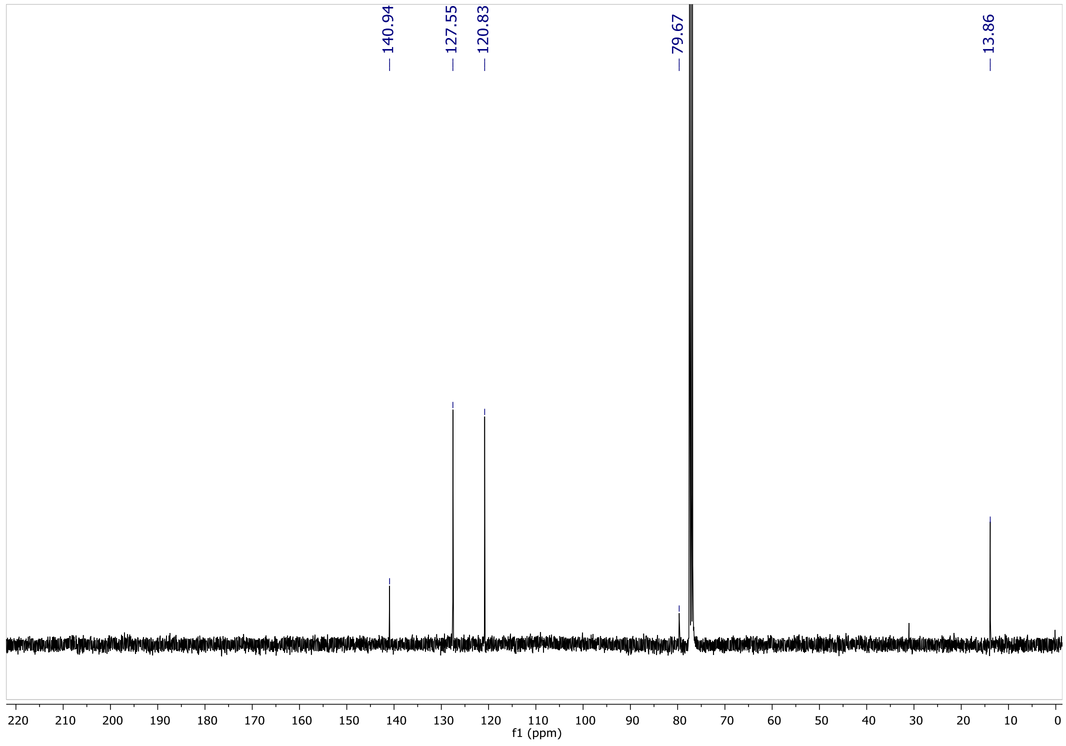

12. References

[1] S. R. Hartmann, E. L. Hahn, *Physical Review* **1962**, *128*, 2042.

[2] B. M. Fung, A. K. Khitrin, K. Ermolaev, *Journal of Magnetic Resonance* **2000**, *142*, 97–101.

[3] M. M. Elsutohy, A. Selo, V. M. Chauhan, S. J. B. Tendler, J. W. Aylott, *RSC Adv* **2018**, *8*, 35840–35848.

[4] S. R. Manne, O. Luna, G. A. Acosta, M. Royo, A. El-Faham, G. Orosz, B. G. De La Torre, F. Albericio, *Org Lett* **2021**, *23*, 6900–6904.

[5] E. E. Bjerg, J. Marchán-García, E. Buxaderas, Y. Moglie, G. Radivoy, *J Org Chem* **2022**, *87*, 13480–13493.

[6] J. I. Bardagi, I. Ghosh, M. Schmalzbauer, T. Ghosh, B. König, *European J Org Chem* **2018**, *2018*, 34–40.

[7] T. M. Alam, H. Fan, *Macromol Chem Phys* **2003**, *204*, 2023–2030.

[8] J. W. Wiench, J. Trébosc, V. S.-Y. Lin, M. Pruski, S. Huh, *J Am Chem Soc* **2005**, *127*, 3057–3068.

[9] M. D. Morales, A. Infantes-Molina, J. M. Lázaro-Martínez, G. P. Romanelli, L. R. Pizzio, E. Rodríguez-castellón, *Molecular Catalysis* **2020**, *485*, 110842.

[10] A. F. Peixoto, S. M. Silva, P. Costa, A. C. Santos, B. Valentim, J. M. Lázaro-Martínez, C. Freire, *Catal Today* **2020**, *357*, 74–83.

[11] Z. Zhao, M. Liu, K. Zhou, Y. Shen, L. Hong, Z. Bao, Q. Yang, Q. Ren, Z. Zhang, *Catal Sci Technol* **2023**, *13*, 4207–4212.

[12] A. Pal, I. Ghosh, S. Sapra, B. König, *Chemistry of Materials* **2017**, *29*, 5225–5231.

[13] A. López-Magano, N. Salaverri, L. Marzo, R. Mas-Ballesté, J. Alemán, *Appl Catal B* **2022**, *317*, 121791.

[14] S. L. Zhu, S. Ou, M. Zhao, H. Shen, C. De Wu, *Dalton Transactions* **2015**, *44*, 2038–2041.

[15] H. Naghili, H. Tajik, K. Mardani, S. M. Razavi Rouhani, A. Ehsani, P. Zare, *Vet Res Forum* **2013**, *4*, 179–83.

[16] S. M. Soria-Castro, B. Lebeau, M. Cormier, S. Neunlist, T. J. Daou, J. P. Goddard, *European J Org Chem* **2020**, *2020*, 1572–1578.

[17] Y. Katsurayama, Y. Ikabata, H. Maeda, M. Segi, H. Nakai, T. Furuyama, *Chemistry - A European Journal* **2022**, *28*, DOI 10.1002/chem.202103223.
